# Quiescence preconditioned nucleus pulposus stem cells alleviate intervertebral disc degeneration by enhancing cell survival *via* adaptive metabolism pattern in rats

**DOI:** 10.3389/fbioe.2023.1073238

**Published:** 2023-02-10

**Authors:** Qi Chen, Qu Yang, Chongzhi Pan, Rui Ding, Tianlong Wu, Jian Cao, Hui Wu, Xiaokun Zhao, Bin Li, Xigao Cheng

**Affiliations:** ^1^ Department of Orthopedics, The Second Affiliated Hospital of Nanchang University, Nanchang, Jiangxi, China; ^2^ Second Clinical Medical College, Nanchang University, Nanchang, Jiangxi, China; ^3^ Institute of Orthopedics of Jiangxi Province, Nanchang, Jiangxi, China; ^4^ Institute of Minimally Invasive Orthopedics, Nanchang University, Nanchang, Jiangxi, China

**Keywords:** intervertebral disc degeneration, nucleus pulposus stem cells, quiescence, cell survival, cell metabolism

## Abstract

Quiescence is a cellular state of reversible growth arrest required to maintain homeostasis and self-renewal. Entering quiescence allows the cells to remain in the non-dividing stage for an extended period of time and enact mechanisms to protect themselves from damage. Due to the extreme nutrient-deficient microenvironment in the intervertebral disc (IVD), the therapeutic effect of cell transplantation is limited. In this study, nucleus pulposus stem cells (NPSCs) were preconditioned into quiescence through serum starvation *in vitro* and transplanted to repair intervertebral disc degeneration (IDD). *In vitro*, we investigated apoptosis and survival of quiescent NPSCs in a glucose-free medium without fetal bovine serum. Non-preconditioned proliferating NPSCs served as controls. *In vivo*, the cells were transplanted into a rat model of IDD induced by acupuncture, and the intervertebral disc height, histological changes, and extracellular matrix synthesis were observed. Finally, to elucidate the mechanisms underlying the quiescent state of NPSCs, the metabolic patterns of the cells were investigated through metabolomics. The results revealed that quiescent NPSCs decreased apoptosis and increased cell survival when compared to proliferating NPSCs both *in vitro* and *in vivo*, as well as maintained the disc height and histological structure significantly better than that by proliferating NPSCs. Furthermore, quiescent NPSCs have generally downregulated metabolism and reduced energy requirements in response to a switch to a nutrient-deficient environment. These findings support that quiescence preconditioning maintains the proliferation and biological function potential of NPSCs, increases cell survival under the extreme environment of IVD, and further alleviates IDD *via* adaptive metabolic patterns.

## 1 Introduction

Intervertebral disc degeneration (IDD) is a primary cause of lower back pain and disability, which directly affects the quality of life and work efficiency of the individual, causing a serious social burden (Jin et al., 2020; Chou, 2021; Costăchescu et al., 2022). Serious disc degeneration can lead to a series of degenerative clinical syndromes, such as lumbar disc herniation, spinal stenosis, and spinal instability, which are predominantly treated by surgical methods (Zhao et al., 2019; Wu et al., 2020). Nevertheless, traditional surgery can only decrease the clinical symptoms of IDD but cannot fundamentally correct the degeneration of the disc. Therefore, cell biotherapy for intervertebral disc (IVD) repair has become a research focus in the past decade.

To the best of our knowledge, only few active cells are present in the disc, and the number and function of cells are further decreased during disc degeneration (Roberts et al., 2006; Ma et al., 2019). Therefore, replenishing the functional cells is the most direct approach to restoring disc homeostasis (Sakai and Grad, 2015; Tong et al., 2017). Presently, multiple cell types have been evaluated *in vitro* and *in vivo* and are being used for intradiscal transplantation. Cells used for transplantation include nucleus pulposus cells (NPCs), nucleus pulposus stem cells (NPSCs), and stem cells from other sources (Chen et al., 2016; Kumar et al., 2017; Wang et al., 2018; Ekram et al., 2021; Li et al., 2021). NPSCs are one of the most ideal seed cells because they are derived from NP, which have a phenotype similar to the IVD cells with good proliferative ability (Hu et al., 2018; Du et al., 2021). Nevertheless, how the cells maintain efficient survival after transplanting into the disc is unknown (Sakai and Grad, 2015; Tong et al., 2017). Because the IVD is an avascular organ, the microenvironment of its inner cells is characterized by poor blood supply and nutrient deficiency (ND), and the blood supply is further diminished during the process of IDD (Urban et al., 2004; Roberts et al., 2006; Ma et al., 2019). Therefore, transplanted cells are difficult to keep alive. Consequently, the transplanted cells must first survive in the extreme environment of IVD with poor nutrition and blood supply for cell therapy (Sakai and Grad, 2015; Tong et al., 2017; Yuan et al., 2020; Binch et al., 2021).​ Moreover, these transplanted cells should maintain favorable bioactivity to produce an extracellular matrix rich in proteoglycan and collagen under the harsh disc environment (Zhang et al., 2019). To conclude, maintaining the survival and biological function of transplanted cells in the harsh degenerated disc microenvironment is important.

Many previous studies have shown that adult stem cells are present in a quiescent state *in vivo*. Quiescence is a state of reversible growth arrest wherein the cells exit the cell cycle and enter the G0 phase, albeit retaining their capacity to divide (Cho et al., 2019; Marescal and Cheeseman, 2020). Once quiescent cells receive activation signals, they can promptly re-enter the cell cycle to start the S-phase and resume proliferation, thereby re-performing biological functions (Rodgers et al., 2014; Goel et al., 2017; Urban and Cheung, 2021). These properties make quiescence play an important role in maintaining stem cell stemness and tissue homeostasis. Research has indicated that stem cells can be induced into quiescence by serum starvation or contact inhibition *in vitro*. Quiescent stem cells can strengthen stemness, resist stress reactions, and improve the tissue repair effect (Wong et al., 2017; Alekseenko et al., 2018; Rumman et al., 2018). Moreover, basal metabolic activity, biosynthesis, and energy requirements are significantly decreased in quiescent stem cells, which favors the adaptive survival of transplanted cells in adverse environments (Ho et al., 2017; Moya et al., 2017; Ferro et al., 2019). Accordingly, we hypothesized that quiescence preconditioning of NPSCs can increase the tolerance of the cells in response to the extreme environment of the degenerated disc after transplantation. We have confirmed the existence of quiescence in NPSCs by serum starvation previously *in vitro* and showed that quiescent NPSCs (Q-NPSCs) can maintain regenerative capacity (Li et al., 2019). ​Nevertheless, it is unknown whether Q-NPSCs can maintain survival and function under the ND environment for disc repair. ​

In the present study, we investigated the characteristics of Q-NPSCs and the survival of the cells under the ND condition *in vitro*, and then determined the therapeutic effect of the cells in a rat model of IDD. We also studied the metabolic characteristics of Q-NPSCs through metabolomic analysis and elucidated the potential mechanism of the cells in maintaining homeostasis.

## 2 Materials and methods

### 2.1 Isolation and culture of primary rat NPSCs

All animal experiments in this study were approved by the Animal Care and Use Committee of Nanchang University (China) and followed by the Guide for the Care and Use of Laboratory Animals by the National Institutes of Health (NIH). Animals were bred in a natural ventilated room with a dark/light cycle at 20°C–25°C, at *ad libitum* access to water and food.

Primary rat NPSCs were isolated using a low-density culture method, as reported elsewhere (Zhao et al., 2017; Wang et al., 2019; Li et al., 2020). In brief, SD rats were sacrificed by cervical dislocation after inhaling an overdose of isoflurane, and then caudal disc specimens were collected. The NP tissues were separated from the rat coccygeal IVD and washed twice with PBS under sterile conditions. Then, the tissues were digested with 0.2% collagenase type Ⅱ (Sigma, United States) solution at 37°C for 2 h. After centrifugation at 1000 rpm for 5  min, the cell pellets were cultured in Dulbecco’s Modified Eagle Medium/Nutrient Mixture F-12 (DMEM/F12, Gibco, United States) medium supplemented with 10% fetal bovine serum (FBS) and 1% penicillin–streptomycin combination at 37°C under a 5% CO_2_ atmosphere. The medium was changed twice a week until sufficient cell colonies were observed, and the cells were digested with 0.25% trypsin-EDTA solution (Gibco) for subculturing. Finally, NPSCs up to the third passage were applied for subsequent experiments.

### 2.2 Identification of the differentiation capacity of rat NPSCs

The osteogenic, chondrogenic, and adipogenic differentiation capacities of NPSCs were determined by Alizarin red staining, Alcian blue staining, and Oil Red O staining, following the manufacturer’s instructions (Cyagen, Guangzhou, China) and the methods of the previous study (Li et al., 2019). The osteogenic differentiation of NPSCs was induced with osteogenic differentiation medium (RASMX-90021, Cyagen) containing 10% FBS, 1% penicillin-streptomycin, 1% glutamine, 1% *β*-glycerophosphate, 0.2% ascorbate and 0.01% dexamethasone. After 3 weeks, Alizarin red staining was used to identify mineral deposits. The cells were fixed at room temperature with 4% paraformaldehyde (PFA) for 30 min, and then stained with an Alizarin Red solution for 10 min at room temperature. Then, the cells were washed with PBS and observed microscopically.

Chondrogenic differentiation was induced by a high-density method (Iezaki et al., 2019). NPSCs were suspended to a cell density of 1 × 10^7^ cells per ml. 10 μl were dripped into a the 6-well plate and placed in the incubator for 1.5 h. After that, chondrogenic medium (RASMX-90042, Cyagen) was added for culture, taking care not to destroy the droplet. The medium was changed every 2 days until the sixth day. The cells were fixed with 4% PFA for 10 min, washed twice using PBS, and incubated in Alcian Blue solution for 2 h. Then, the cultures were washed again with PBS and observed by optical microscope.

Adipogenic differentiation was induced with adipogenic medium (RASMX-90031, Cyagen), including induction medium A (containing 10% FBS, 1% penicillin-streptomycin, 1% L-glutamine, 0.2% insulin, 0.1% 3-Isobutyl-1-methylxanthine, 0.1% rosiglitazone and 0.1% dexamethasone) and maintenance medium B (containing 10% FBS, 1% penicillin-streptomycin, 1% L-glutamine and 0.2% insulin). The NPSCs were induced with solution A for 3 days and solution B for 1 day, alternating until lipid-rich droplets appeared. The cells were fixed and then stained with Oil Red O solution for 30 min. After washing with PBS, the culture plates were placed under a microscope for observation.

### 2.3 Identification of stem cell surface markers

The stem cell specific markers CD73, CD90, CD105, CD34, CD45, and HLA-DR were analyzed by flow cytometry. Briefly, rat NPSCs were digested with 0.25% trypsin and collected. The cells were centrifuged at 300 *g* for 5 min, washed twice with PBS, and finally resuspended in 0.1 ml of PBS. ​The samples were incubated at room temperature with primary antibodies CD73-PE (Bs-4834R, Bioss, Beijing, China), CD90-PE (Bs-0778R, Bioss), CD105-APC (67075-1-lg, Proteintech, Wuhan, China), CD34-FITC (Bs-0646R, Bioss), CD45-FITC (Bs-4819, Bioss), and HLA-DR-APC (Bs-1198R, Bioss) for 30 min, washed twice with PBS, and then incubated in the dark with secondary antibodies for 30 min​ After being washed again twice with PBS, they were detected by flow cytometry.

### 2.4 Induction of quiescence and reactivation in NPSCs

NPSCs (2 × 10^5^) were seeded in a 25-cm^2^ cell-culture flask and cultured in a standard medium (DMEM/F12 supplemented with 10% FBS). After 24 h, the cells were used as proliferating NPSCs (P-NPSCs). At this time point, another group of NPSCs were rinsed with PBS and cultured in a low-serum medium (DMEM/F12 supplemented with 0.1% FBS) for 48 h to induce quiescence, as quiescent NPSCs (Q-NPSCs). Next, the medium was changed every 3 days with a low-serum medium, and, on day 7, with the standard medium in order to stimulate the cells. After 24 h, the cells were used as reactivated NPSCs (Re-NPSCs).

### 2.5 Nutrient-deficient culture conditions

*In vitro*, the degenerative disc microenvironment was simulated by culturing cells in a glucose-free DMEM without FBS, which was defined as the nutrient deficiency (ND) condition (Liu et al., 2017; Wang et al., 2020). The cell experiments were divided into four groups based on the participating cell types: P-NPSCs (proliferating NPSCs), P-ND-NPSCs (proliferating NPSCs cultured under ND), Q-NPSCs (quiescent NPSCs), and Q-ND-NPSCs (quiescent NPSCs cultured under ND). Quiescent cells were prepared 2 days in advance (Day-2), and the proliferating cells were prepared 1 day in advance (Day-1). On Day 0, the medium for both P-ND and Q-ND was replaced with a glucose-free DMEM without FBS and subsequently cultured without any medium change.

### 2.6 Cell cycle analysis

The cell cycle of NPSCs was detected by flow cytometry. Briefly, the samples were digested with 0.25% trypsin, collected, and then washed twice with PBS. Then, the cells were fixed in precooled 75% ethanol and stored at −20°C until further use. To analyze the cell cycle, the fixed cells were centrifuged and the ethanol was discarded. Then, the cells were hydrated with PBS for 15 min, and the supernatant was discarded after centrifugation. Then, 1 mL of the DNA staining solution (Multi Sciences, Hangzhou, China) was added and mixed well. The stained cells were incubated in the dark at room temperature for 30 min, followed by flow cytometry analysis.

### 2.7 Immunofluorescence

The cell slides prepared earlier were fixed in 4% paraformaldehyde for 15 min and then permeated with 0.5% Triton X-100 for another 15 min at room temperature. The cells were then blocked with 10% goat serum for 30 min and incubated with a primary antibody at 4°C overnight. Then, the cells were treated with secondary antibodies in the dark at room temperature for 1 h and counter-stained with DAPI for 5 min. After sealing the slices, the images were observed by fluorescence microscopy and subjected to semi-quantitative analysis using ImageJ.

### 2.8 Cell proliferation, apoptosis, and survival analysis

The Cell Counting Kit-8 (CCK-8; Solarbio, Beijing) was used to detect the cell viability of NPSCs. In accordance with the above-mentioned methods, NPSCs were seeded into a 96-well plate at the density of 5000 cells/well, and 100 μl of the medium was added to each well. For detection purpose, 10 μl of the CCK-8 solution was added to each well and incubated for 3 h. The optical density (OD) at 450 nm was measured using an enzyme-labeled instrument.

The apoptosis of NPSCs was detected using the FITC-Annexin V/PI Kit (UE, Suzhou, China). First, the cells were digested with EDTA-free trypsin solution and then centrifuged, followed by washing twice with pre-cooled PBS. The resultant cells were collected and resuspended with 100 μl of 1× binding buffer, followed by the addition of 5 μl FITC-Annexin V (AV) and 5 μl PI working solution. In addition, AV and PI single-stained cells were prepared for flow compensation regulation. After incubation at room temperature for 15 min, the samples were analyzed by flow cytometry.

The Hoechst 33342/PI Staining Kit (Solarbio) was used to label the living and dead cells. According to the manufacturer’s instruction, the cells were treated with a staining buffer and dyed at 4°C for 30 min in the dark. Finally, the images were observed using a fluorescence microscope. Hoechst 33342-positive and PI-negative cells were designated as viable cells, whereas Hoechst 33342-positive and PI-positive cells were designated as dead cells.

Cell survival was determined by performing the Trypan Blue Exclusion Dye assay. Briefly, the cells were digested with trypsin and centrifuged at 1000 rpm for 1 min, and the supernatant was discarded and resuspended in 900 μl of PBS. Then, 100 μl of 0.4% trypan blue (Gibco) solution was added, mixed, and stained for 3 min. A small amount of the cell suspension was absorbed and counted in a blood cell-counting plate. The dead cells were stained blue, while the living cells were unstained. The cell survival rate was expressed as the percentage of viable cells relative to total cells at each time point. In addition, the NPSCs were stained with hematoxylin eosin (HE) dye and observed under a microscope.

### 2.9 Cell retention study in isolated intervertebral discs

To investigate the survival of NPSCs after implantation into the disc. We constructed a rat IVD organ culture model (Wu et al., 2019; Liao et al., 2021). The tail disc with an intact endplate was isolated from 3-month-old SD rats (300 g) and cultured in DMEM/F12 medium supplemented with 15% FBS and 1% penicillin/streptomycin at 37°C. A solution of 1.5 M NaCl and 0.4 M KCl was added to adjust the osmotic pressure of the medium to 400 mOsm, which is similar to the physiological conditions. NPSCs with a positive GFP expression from GFP transgenic SD rats (donated by Xinqiao Hospital) were employed for tracing. The cell suspension (2 μl) containing 1 × 10^4^ P-NPSCs(GFP+) or Q-NPSCs(GFP+) cells was slowly injected into the disc using a 31G needle, and the injection was kept inserted at the site for about 5 min to prevent any leakage. The culture medium was replaced every 3 days. At each time point, the cultured discs were observed using an animal imaging system (AniView600, BLT, Guangzhou, China), and the fluorescence intensity of each membrane was subsequently quantified.

### 2.10 Rat IDD model induction and NPSCs implantation

Following anesthesia, the tail was disinfected with iodophor, and a 21G-puncture needle was used to puncture different levels of the tail IVD at a depth of 5 mm. The needle was inserted into the center of the nucleus pulposus through the annulus fibrosus and rotated by 360° and maintained for 30 s. After 2 weeks, the caudal discs of different levels were divided into four groups: Control (not punctured), P-NPSCs (punctured and injected with P-NPSCs(GFP+)), Q-NPSCs (punctured and injected with Q-NPSCs(GFP+), and PBS (punctured and injected with PBS) groups. The injection protocol was performed as described in Section 2.9. The segments were injected with 2 μl cell suspension (containing 1 × 10^4^ cells) or 2 µl PBS.

### 2.11 Radiographic analysis

X-ray were taken to assess the disc height at 0, 2 and 4 weeks after cell injection. Briefly, the animal was placed in the prone position with the tail straight and the ray perpendicular to the tail. The disc height index (DHI) was calculated according to the method described by Han et al. (Han et al., 2008) to evaluate the disc height. Changes in DHI were normalized to the preoperatively measured DHI and expressed as %DHI (= measured DHI/before acupuncture DHI × 100). All images were measured by three independent observers who were blinded to the experimental design.

### 2.12 Histological analysis

Two weeks after the cell transplantation, the rats were sacrificed and their caudal disc tissues were assessed. These tissues were fixed in 4% paraformaldehyde, decalcified, and embedded in paraffin. For the immunohistochemistry analysis, the sections were incubated with Collagen-Ⅱ (1:200, 28459-1-AP, Proteintech) or Aggrecan (1:200, 13880-1-AP, Proteintech) antibodies. On the other hand, the sections were stained with hematoxylin–eosin (HE), saffron O-fast green (SO), and toluidine blue (TB). The images were then observed under the microscope (Olympus, Tokyo, Japan). The degree of disc degeneration was assessed based on the historical grading of the disc degeneration (Table 1) (Zhou et al., 2018). The control, Q-NPCSs, P-NPSCs and PBS groups each contained six samples and were scored by three independent observers.

**TABLE 1:** The histological grading of the disc degeneration.

Cellularity and morphology	Grade
Cellularity of the annulus fibrosus	1. Fibroblasts comprise more than 75% of the cells
	2. Neither fibroblasts nor chondrocytes comprise more than 75% of the cells
	3. Chondrocytes comprise more than 75% of the cells
Morphology of the annulus fibrosus	1. Well-organized collagen lamellae without ruptured or serpentine fibers
	2. Inward bulging, ruptured or serpentine fibers in less than one-third of the annulus
	3. Inward bulging, ruptured or serpentine fibers in more than one-third of the annulus
Border between the annulus fibrosus and nucleus pulposus	1. Normal, without any interruption
	2. Minimal interruption
	3. Moderate or severe interruption
Cellularity of the nucleus pulposus	1. Normal cellularity with stellar-shaped nuclear cells evenly distributed throughout the nucleus
	2. Slight decrease in the number of cells with some clustering
	3. Moderate or severe decrease (>50%) in the number of cells with all the remaining cells clustered and separated by dense areas of proteoglycans
Morphology of the nucleus pulposus	1. Round, comprising at least half of the disc area in mid-sagittal sections
	2. Rounded or irregularly shaped, comprising one-quarter to half of the disc area in mid-sagittal sections
	3. Irregularly shaped, comprising less than one-quarter of the disc area in mid-sagittal sections

### 2.13 Real-time quantitative PCR

Total RNA was extracted from the cells with TRIzol reagent (Invitrogen, United States) and transcribed into cDNA using a reverse transcription kit (Takara, Japan), which was amplified by using the SYBR Green Master Mix (Takara). Then, the mRNA expression levels of PCNA, Ki67, Sox9, Acan, and Col2a1 were detected by using the Real-time ABI 7900 HT System (Applied Biosystems, CA, United States), using GAPDH as the internal reference. The results of the experiment were calculated using the 2^−ΔΔCT^ method. The primers are listed in Table 2.

**TABLE 2:** Primes sequences.

Gene	Forward primer	Reverse primer	Id
PCNA	GCC​CTC​AAA​GAC​CTC​ATC​AAT	ATC​AGC​ATT​ATC​TTC​AGC​CCT​TA	NM_022381.3
Ki67	CCA​TTA​ACA​AGA​GTG​AGG​GAG​TG	TGA​GTG​GAG​TAT​TAG​GAG​GCA​AG	XM_006230453.2
Sox9	CAC​ATC​TCT​CCT​AAC​GCC​ATC​T	GCG​GCA​GGT​ATT​GGT​CAA​ACT​C	NM_080403.2
Acan	AGT​GAC​CCA​TCT​GCT​TAC​CCT​G	CTG​CAT​CTA​TGT​CGG​AGG​TAG​TG	XM_039101034.1
Col2a1	GTG​TCA​AGG​GTC​ACA​GAG​GTT​AC	CGC​TCT​CAC​CCT​TCA​CAC​CT	NM_012929.1
GAPDH	CTG​GAG​AAA​CCT​GCC​AAG​TAT​G	GGT​GGA​AGA​ATG​GGA​GTT​GCT	NM_017008.4

### 2.14 Metabolomics analysis

To detect the differences in the metabolic patterns between P-NPSCs and Q-NPSCs, the cells were collected (four samples from each group), and it was ensured that the number of cells in each sample was not less than 1 × 10^6^. The cells were centrifuged and the supernatant was discarded. Then, the wet pellets were flash-frozen under liquid nitrogen for 30 s and then transferred to −80°C for storage until use. Widely targeted/non-target metabolomics experiment was performed at the Biomarker Technologies (Beijing, China) using the following process: after further treatment of the sample with methanol and acetonitrile, metabolomic analysis was performed by using an LC/MS system consisting of Waters Acquity I-class PLUS ultra-high-performance liquid chromatography tandem equipped with the Waters Xevo G2-XS QTOF high-resolution mass spectrometer. The raw data collected using MassLynx V4.2 was processed by the Progenesis QI software for peak extraction, peak alignment, and other data processing operations. The identified compounds were searched for classification and pathway information in the KEGG, HMDB, and lipidmaps databases. According to the grouping information, the difference multiples were calculated and compared, and *t*-test was applied to calculate the difference significance *p*-value of each compound. The R language package ropls was used to perform the orthogonal partial least squares discriminant analysis (OPLS-DA) modeling, and 200-times permutation tests was performed to verify the reliability of the model. The VIP (Variable Importance in Projection) value of the model was calculated through multiple cross-validation to measure the effect of each metabolite expression pattern on the classification of each sample, thus assisting the filtrated metabolites. The method of combining the difference multiple, the *p*-value, and the VIP value of the OPLS-DA model was adopted to screen the differential metabolites. The screening criteria were FC > *p*-value <0.05 and VIP >1. The difference metabolites of the KEGG pathway enrichment significance were calculated using the hypergeometric distribution test.

### 2.15 Statistical analysis

All experiments were performed in triplicate. The mean ± standard deviation (SD) was applied to present the data. Graphics and statistical analyses were conducted by GraphPad Prism eight software. Student’s t-test for two groups and one-way ANOVA with Tukey *post hoc* test for three or more groups were used for group comparisons. *p* < 0.05 was considered to indicate statistical significance.

## 3 Results

### 3.1 Characterization of NPSCs

**FIGURE 1:**
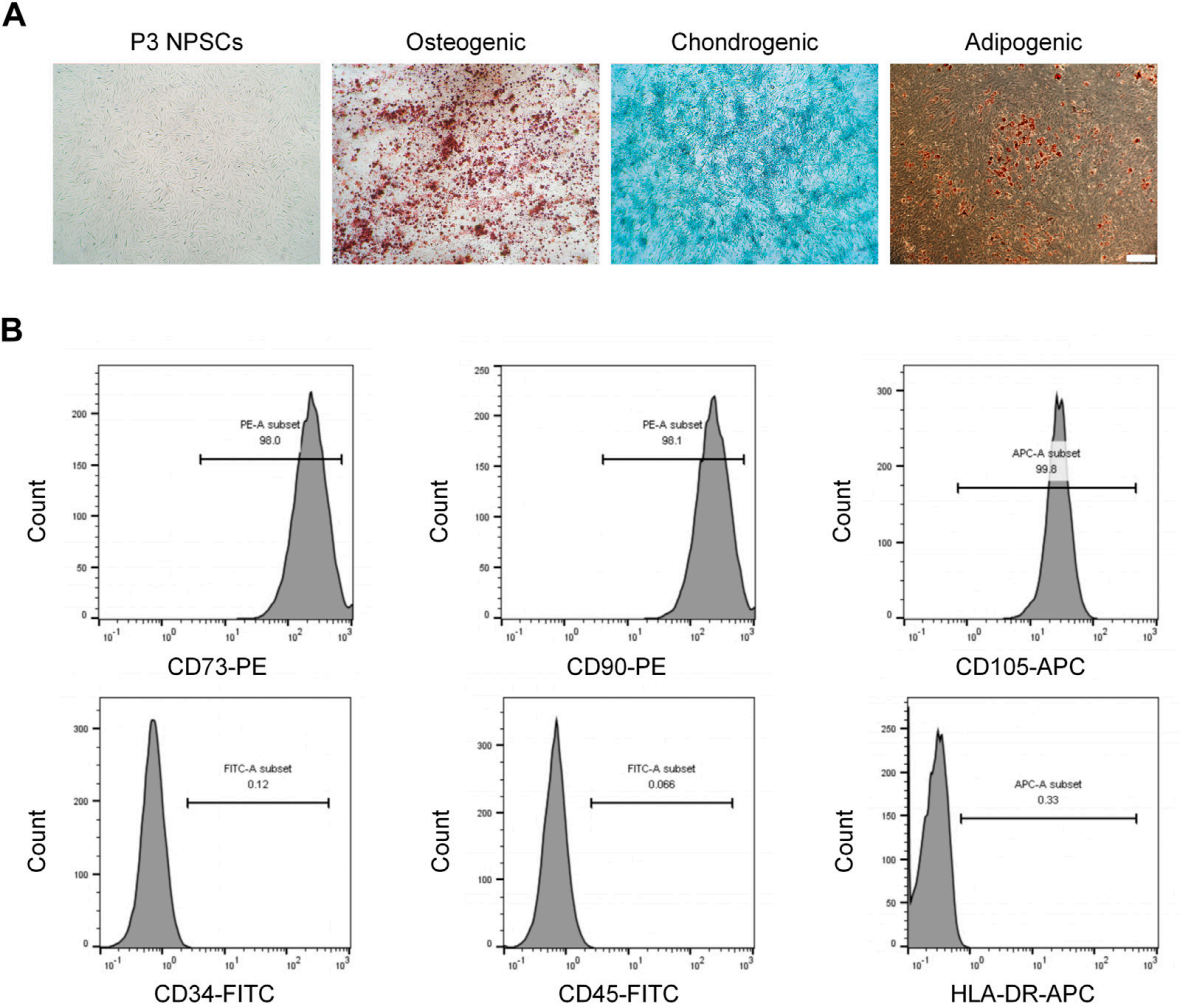
Morphological characteristics and identification of nucleus pulposus stem cells (NPSCs). **(A)** P3 NPSCs exhibited a long spindle shape with a uniform morphology and whirlpool arrangement. Osteogenic, chondrogenic, and adipogenic differentiation capacities of NPSCs were identified by Alizarin Red staining, Alcian blue staining, and Oil Red O staining, respectively. **(B)** Cell surface markers of NPSCs were detected by flow cytometry. The NPSCs positively expressed CD73, CD90 and CD105 and negatively expressed CD34, CD45, and HLA-DR.

After three passages, the cells were purified; they exhibited a long spindle shape with a uniform morphology and whirlpool arrangement (Figure 1A). After inducing osteogenic differentiation of the NPSCs, the formation of mineralized nodules was determined by Alizarin red staining. For the chondrogenic differentiation, Alcian blue staining showed a large amount of glycosaminoglycan accumulation in the cells. Oil Red O staining showed many intracellular lipid droplets after adipogenic differentiation (Figure 1A). For the uninduced NPSCs in these stains, no significant positive stains were observed, as demonstrated in the previous study (Li et al., 2019). Furthermore, the expressions of NPSCs surface markers CD73 (+), CD90 (+), CD105 (+) were 98.0%, 98.1%, and 99.8%, respectively, whereas the expressions of CD34 (−), CD45 (−), HLA-DR (−) were 0.12%, 0.07%, and 0.33% respectively, by flow cytometry (Figure 1B). To conclude, these results showed that the NPSCs had higher purity and stemness, which was suitable for the treatment of IVD.

### 3.2 Quiescent NPSCs sustained favorable potential for proliferation and biological function

**FIGURE 2:**
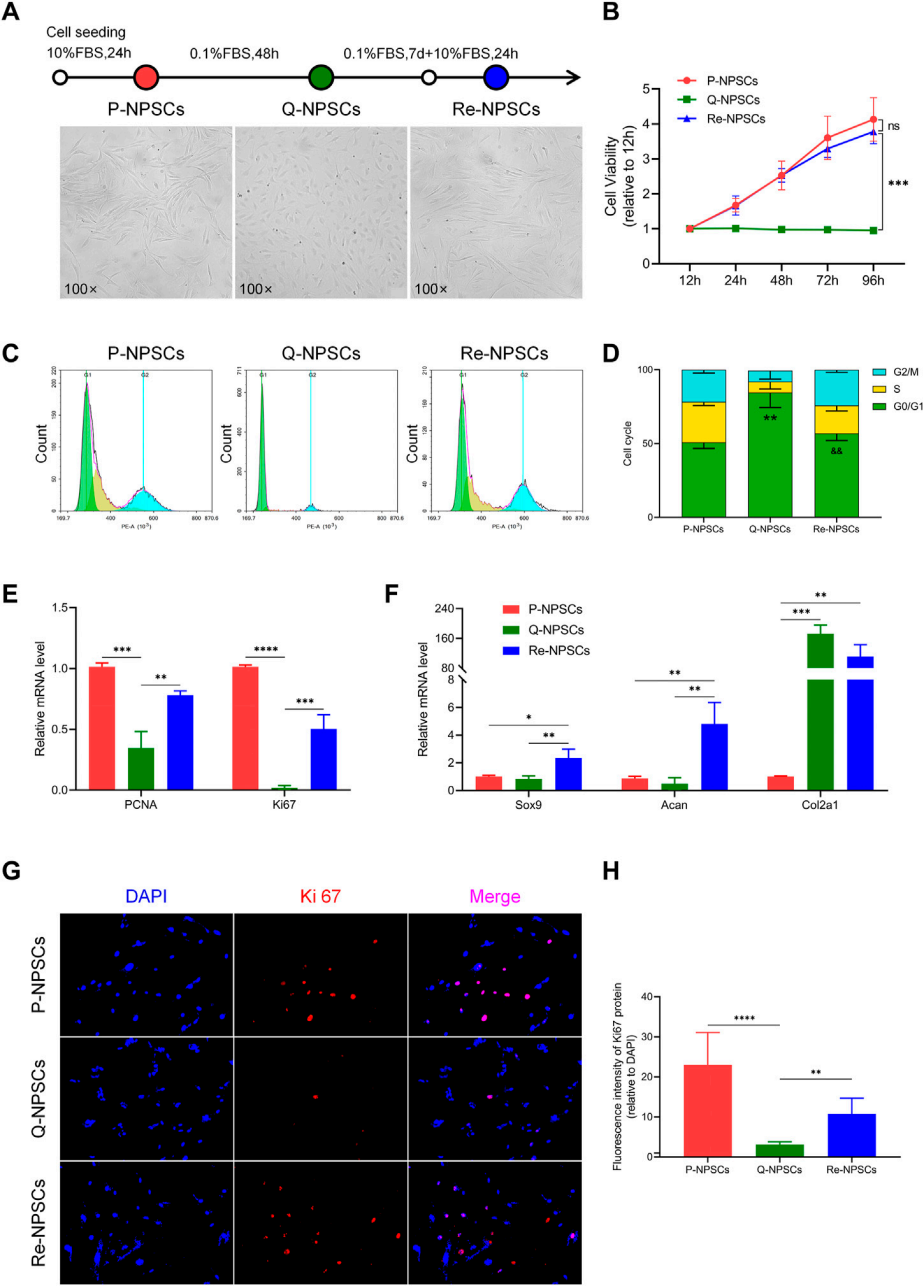
Morphological characteristics, proliferative potentials, and biological functions of the NPSCs were analyzed before and after quiescence induction and reactivation. **(A)** The morphology changes of P-NPSCs, Q-NPSCs and Re-NPSCs. **(B)** The cell viability of NPSCs in each group were detected by CCK-8 assay. ^ns^ *p* > 0.05, ^***^ *p* < 0.001. **(C,D)** The cell cycle of NPSCs in each group were analyzed by flow cytometry and through statistical analysis. ^**^ *p* < 0.01, P-NPSCs vs. Q-NPSCs; ^&&^ *p* < 0.01, Q-NPSCs vs. Re-NPSCs. **(E,F)** The relative mRNA levels of PCNA and Ki67 **(E)** as well as that of Sox9, Acan and Col2a1 **(F)** were analyzed by qRT-PCR. **(G,H)** The protein expression of Ki67 was detected by immunofluorescence **(G)**, followed by quantitative analysis by ImageJ software **(H)**. ^*^ *p* < 0.05, ^**^ *p* < 0.01, ^***^ *p* < 0.001, ^****^ *p* < 0.0001. P-NPSCs: proliferating NPSCs; Q-NPSCs: quiescent NPSCs; Re-NPSCs: reactivated NPSCs.

The quiescent state of NPSCs was induced by serum starvation (DMEM/F12 medium containing 0.1% FBS) and its characteristics were observed. Compared with the proliferating NPSCs (P-NPSCs), the growth of the quiescent NPSCs (Q-NPSCs) was arrested and the cells gradually shrunk and became smaller. At the same time, the cell morphology gradually changed from a long spindle shape to a round or polygon, and the transparency increased. After 7 days, Q-NPSCs were reactivated with DMEM/F12 medium containing 10% FBS. It can be seen that the cells exit the quiescent state and start to proliferate again. It manifested that the cell morphology gradually expands and recovers the long spindle shape (Figure 2A). According to the CCK-8 assay, Q-NPSCs had low or no growth activity compared with P-NPSCs. However, after reactivation, the NPSCs showed the same growth activity as the P-NPSCs and continued to rise over time (Figure 2B). Flow cytometry was adopted to examine the cell cycle (Figures 2C, D). The percentage of Q-NPSCs in the G0/G1 phase was significantly higher than that of P-NPSCs and reactivated NPSCs (Re-NPSCs). In addition, the Q-NPSCs can maintain pluripotent differentiation potential. In our pervious study (Li et al., 2019), we have observed that the Re-NPSCs can be induced to osteogenic, chondrogenic, and adipogenic differentiation just like the Q-NPSCs. These results showed that serum starvation can induce NPSCs to enter the G0 phase, which was defined as the quiescent state. After reactivation, the cells re-entered the cell cycle and resumed proliferation.

Moreover, the expression of genes and the levels of proteins were evaluated to determine the biological activity of each group. PCNA and Ki67 are classical markers of cellular proliferation. Compared with P-NPSCs, the mRNA levels of PCNA and Ki67 in Q-NPSCs decreased significantly, and the levels of PCNA and Ki67 increased again in Re-NPSCs (Figure 2E). Furthermore, when compared with P-NPSCs, the protein level of Ki67 was decreased in Q-NPSCs. After reactivation, the protein level of Ki67 was restored (Figures 2G,H). In addition, many chondrogenic genes (Sox9, Acan, and Col2a1) that promote extracellular matrix synthesis were detected. In contrast to P-NPSCs, Sox9 and Acan mRNA expression was slightly downregulated in Q-NPSCs but restored in Re-NPSCs. However, an interesting observation was that Col2a1 mRNA expression was significantly increased in Q-NPSCs and Re-NPSCs (Figure 2F). These results showed that the proliferation and partial biological activity of NPSCs are temporarily suppressed by quiescence induction but resume or increase immediately after reactivation.

### 3.3 Quiescence preconditioning favored NPSCs against apoptosis and increase cell survival under nutrient deficiency *in vitro*

**FIGURE 3:**
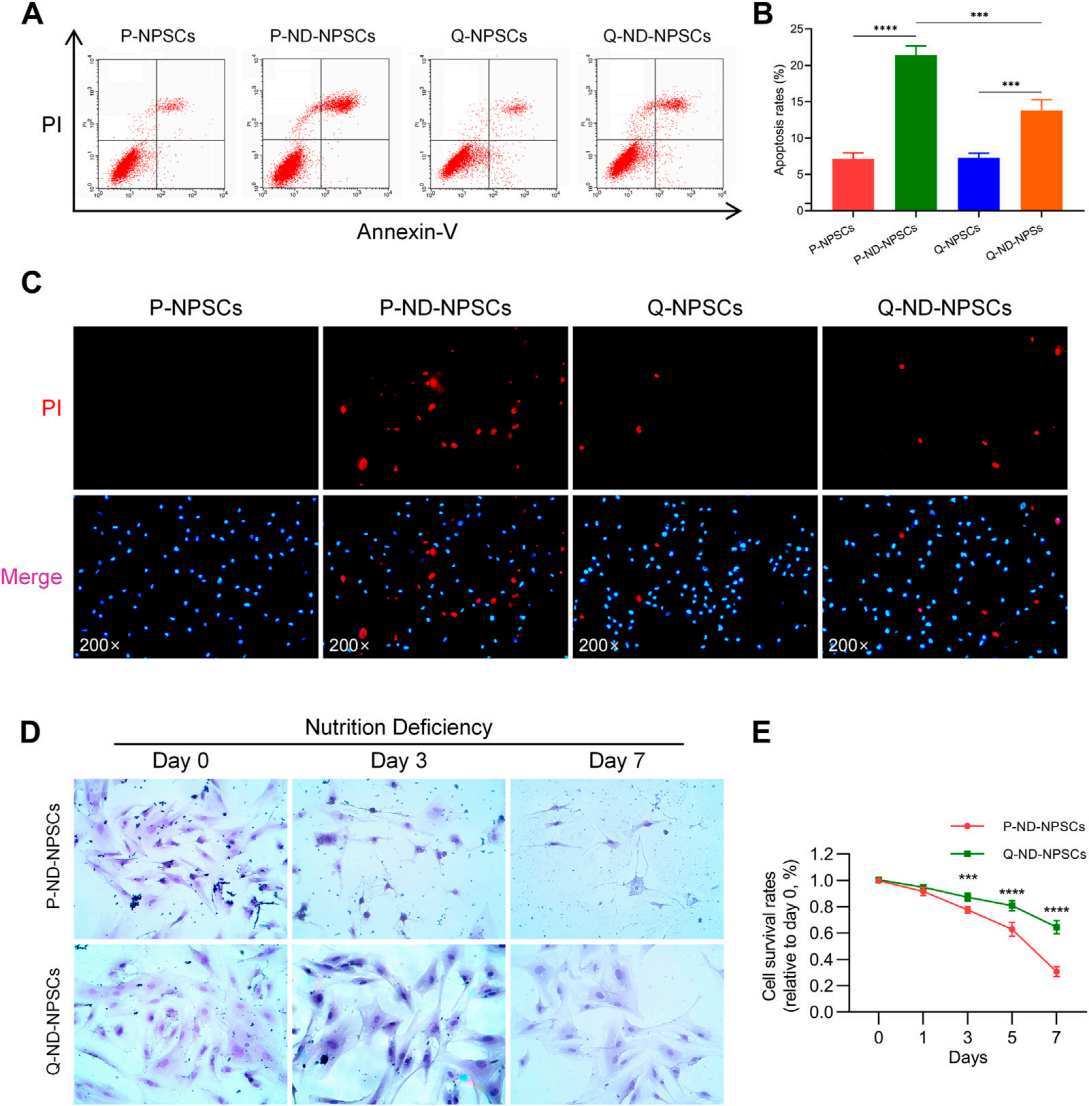
The apoptosis and survival of P-NPSCs and Q-NPSCs were analyzed *in vitro* under nutrient deficiency conditions. **(A,B)** The apoptosis of P-NPSCs, P-ND-NPSCs, Q-NPSCs and Q-ND-NPSCs were analyzed by flow cytometry **(A)** and statistical analyses **(B)**. **(C)** The NPSCs in each group were stained with the Hoechst 33342 dye (blue) and PI dye (red). Blue cells without red color signify living cells and red cells signify dead cells. **(D)** To observe the morphology of the surviving cells in each group, the cells were stained with hematoxylin Eosin (HE). **(E)** The survival of P-ND-NPSCs and Q-ND-NPSCs was analyzed by the Trypan Blue Exclusion Dye assay.

To observe the response of proliferating NPSCs (P-NPSCs) and quiescent NPSCs (Q-NPSCs) in response to the nutrient-deficient (ND) environment, flow cytometry analysis was performed to detect cellular apoptosis. Even though quiescence preconditioning by serum starvation led to apoptosis, the apoptosis rate was less than 10% compared with unpreconditioned NPSCs, which was within the acceptable range. The P-NPSCs and Q-NPSCs were then cultured in the ND condition. After 48 h, both P-NPSCs and Q-NPSCs showed a significant increase in apoptosis, whereas Q-NPSCs had lower apoptosis than P-NPSCs (Figures 3A, 3B). Moreover, Hoechst 33342/PI double staining was used to visualize the living and dead cells at this time (Figure 3C). No obvious PI-positive cells were found in the untreated P-NPSCs, whereas a small number of PI-positive cells were seen in the Q-NPSCs. Under the ND culture condition, the number of PI-positive cells in quiescent NPSCs (Q-ND-NPSCs) was significantly lower than in proliferating NPSCs (P-ND-NPSCs). These results demonstrated that quiescence preconditioning can inhibit apoptosis and reduce cell death under the ND condition.

With the progression of nutrient deprivation, more and more P-ND-NPSCs died, and “ghost cells” appeared (Figure 3D). On the contrary, the Q-ND-NPSCs showed almost no morphological changes and less cell death from day 0 to day 7. The results of the trypan blue exclusion experiment showed (Figure 3E) that the cell survival rate of the P-ND-NPSCs continued to decrease significantly, and reached only about 30% on the seventh day, whereas that of the Q-ND-NPSC decreased slowly and remained above 60% on the seventh day. These results showed that quiescence preconditioning increased the tolerance under the ND environment, thereby improving the cell survival rate.

**FIGURE 4:**
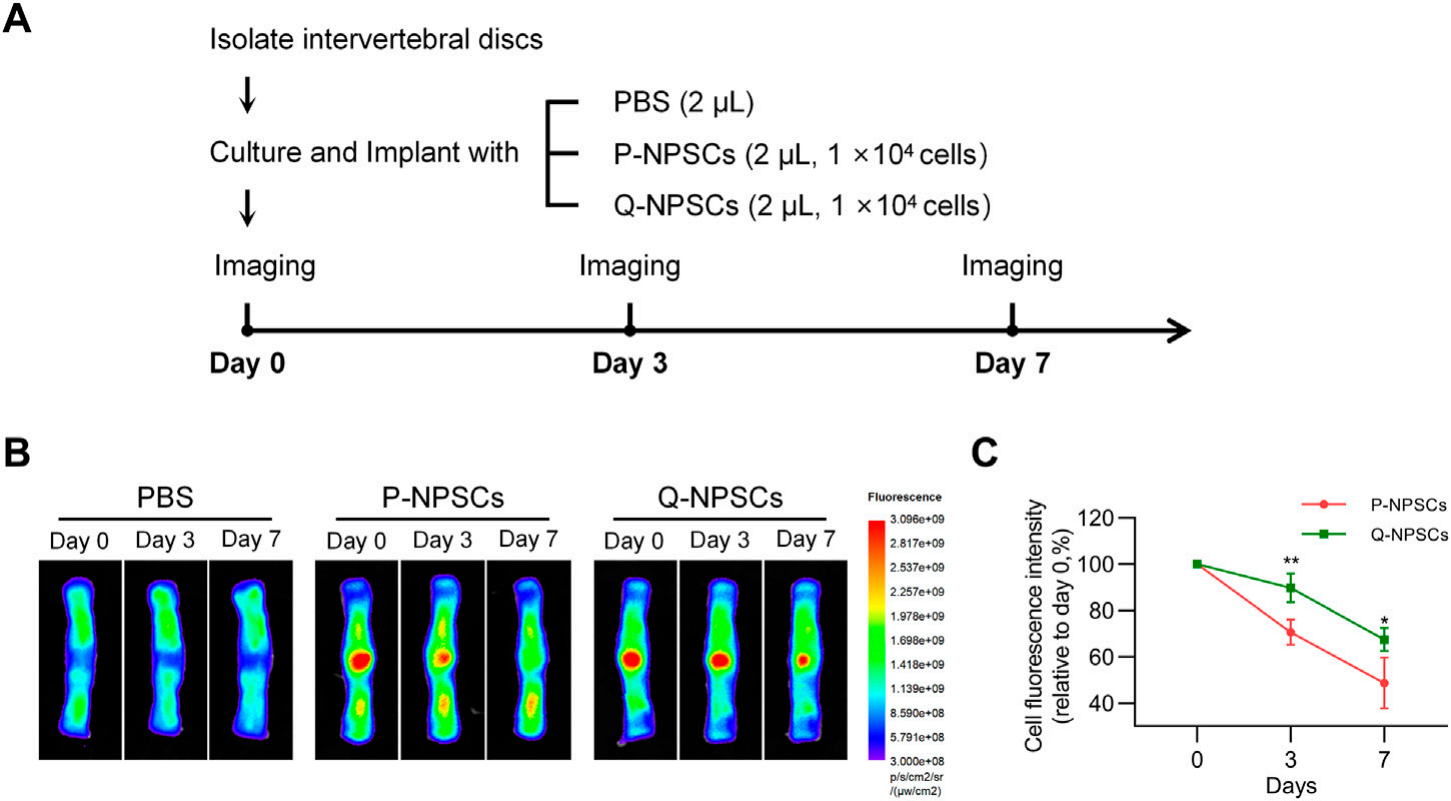
The survival of NPSCs after implantation into isolated discs was measured using an animal fluorescence imaging system. **(A)** Grouping and procedure of the cell retention study. **(B,C)** The fluorescence intensity, which indicates the number of surviving cells, was measured at day 0, day 3 and day 7 after injection of P-NPSCs and Q-NPSCs. PBS was implanted as a control. ^*^ *p* < 0.05, ^**^ *p* < 0.01, ^***^ *p* < 0.001, ^****^ *p* < 0.0001.

Furthermore, the retention of P-NPSCs and Q-NPSCs after implantation in the disc was determined by *in vitro* imaging analysis. As shown in Figure 4, the fluorescence intensity represented cell retention. The fluorescence cells after implantation of P-NPSCs and Q-NPSCs decreased in the IVD. However, the fluorescence intensity of Q-NPSCs was higher than that of P-NPSCs on the seventh day of implantation. These results suggested that quiescence preconditioning also contributed to the survival of NPSCs in the IVD after implantation.

### 3.4 Quiescence preconditioning increased the ability of NPSCs to alleviate the intervertebral disc degeneration *in vivo*

**FIGURE 5:**
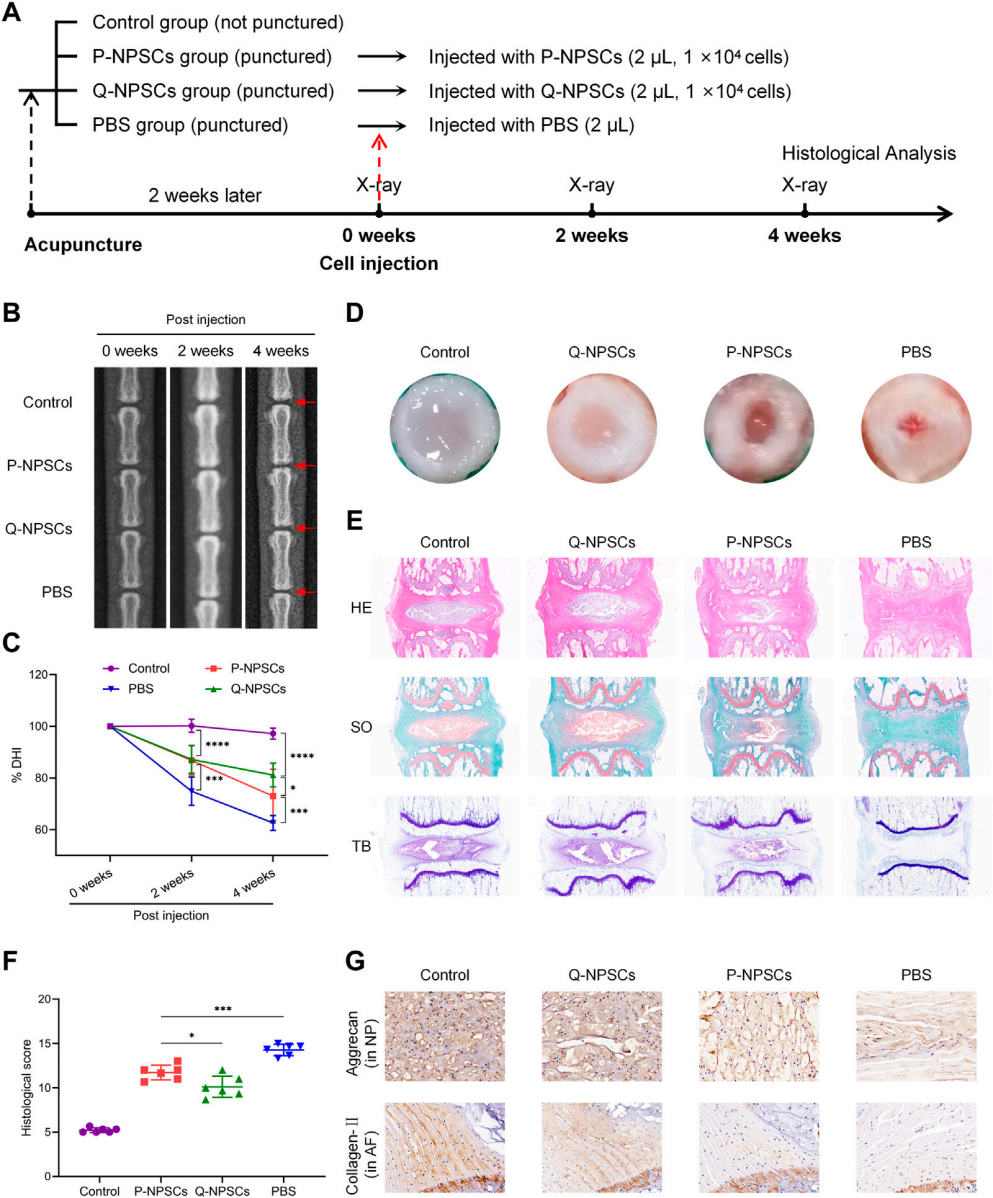
The therapeutic effects of P-NPSCs and Q-NPSCs on intervertebral disc degeneration (IDD) were analyzed and compared *in vivo*. **(A)** Groupings and procedures for *in vivo* experiments. **(B)** X-rays were captured to assess the disc height before the puncture, at 2 weeks after the puncture and then at 2 weeks after the cell injection. The red arrows indicate the location of the disc, as well as the locations of the acupuncture and injection cells. **(C)** The disc height index (DHI) was calculated and expressed as %DHI to assess the change in disc height after cell implantation. **(D)** Two weeks after the cell transplantation, the rat caudal disc tissues were assessed. **(E)** The disc sections were stained with hematoxylin eosin (HE), saffron O-fast green (SO), and toluidine blue (TB). **(F)** The degree of disc degeneration was assessed through histological scoring. **(G)** The protein expression of aggrecan in the nucleus pulposus (NP) and collagen-Ⅱ in the annulus fibrosus (AF) was determined by immunohistochemistry analysis (×100). ^*^ *p* < 0.05, ^***^ *p* < 0.001.

To determine the therapeutic effect of Q-NPSCs transplantation on disc degeneration compared with P-NPSCs *in vivo*, a rat model of IDD was established by needle puncture. After 2 weeks, P-NPSCs, Q-NPSCs, and PBS were administered into different segments of intervertebral discs, where PBS was used as the control. The untreated segment was the normal group. We then performed radiographic analysis and histological analysis of each group after transplantation, as shown in Figure 5A.

First, the intervertebral disc height was observed by X-ray after cell injection and evaluated by disc height index (DHI) (Figures 5B, C). Compared with the normal group, DHI decreased after induction of IDD in the PBS, P-NPSCs, and Q-NPSCs groups. The mean DHI continued to decrease in the PBS group. After 2 weeks of cell transplantation, the DHI of the P-NPSCs group and Q-NPSCs group was significantly higher than that of the PBS group. After 4 weeks of cell transplantation, the DHI of the Q-NPSCs group was significantly higher than that of the P-NPSCs group. All these results suggested that NPSCs transplantation can maintain IVD height and quiescence preconditioning can increase this ability.

Four weeks after transplantation, the caudal discs were harvested to assess histological differences between each group. The macroscopic observation of the IVD was evaluated (Figure 5D). The NP and AF in the IVD without acupuncture were distinctly defined. The AF was complete, and the NP tissue was water-rich. In the PBS group, the AF structure was disordered, and the NP area was significantly reduced and replaced by microscopic tissues. Although AF tissues in the P-NPSC and Q-NPSC groups were thicker than those in the PBS group, they were rich in NP tissues, and the boundary between the NP and AF was clear. Notably, the NP area of the Q-NPSC group was larger than that of the P-NPSC group. HE staining (Figure 5E) showed that NP tissues were arranged neatly and evenly in the normal group. In the PBS group, NP tissues were damaged or disappeared and filled with fibrous connective tissues, whereas AF tissues were disordered and lost their concentric circular lamellar structures. The distribution and structures of the NP and ECM in the P-NPSC and Q-NPSC groups were visible; however, the quantity and structure of the ECM in the Q-NPSC group were better than those in the P-NPSC group. S-O staining (Figure 5E) showed that polysaccharide content in the PBS group decreased significantly; thus, the staining of both groups was stronger than that of the PBS group. Furthermore, the staining of the Q-NPSC group was stronger than that of the P-NPSC group. In contrast, toluidine blue staining (Figure 5E) showed that the P-NPSC and Q-NPSC groups showed more NP chondrocytes and positive proteoglycan staining than the PBS group, and the staining intensity of the Q-NPSC group was better than that of the P-NPSC group. Overall, the histological scores were significantly higher in all experimental groups than those in the normal group and were higher in the PBS group than those in the P-NPSC and Q-NPSC groups. The histological scores of the Q-NPSC group were lower than those of the P-NPSC group (Figure 5F). These results indicated that the transplantation of preconditioned quiescent NPSCs might maintain or improve the tissue structure of the IVD with better effects than the transplantation of non-preconditioned proliferating NPSCs.

Immunohistochemical staining showed that collagen-Ⅱ and aggrecan were present in the normal, P-NPSC, and Q-NPSC groups (Figure 5G). Due to the absence of NP tissues in the PBS group, only small amounts of collagen-II and aggrecan were observed. According to the quantitative analysis, collagen-Ⅱ and aggrecan levels in the P-NPSC and Q-NPSC groups were significantly higher than those in the PBS group and lower than those in the normal group. The collagen-Ⅱ and aggrecan levels in the Q-NPSC group were higher than those in the P-NPSC group. These results suggested that the preconditioned quiescent NPSCs maintained ECM production in the degenerate disc.

### 3.5 Preconditioned quiescent NPSCs adopt an adaptive metabolic pattern

**FIGURE 6:**
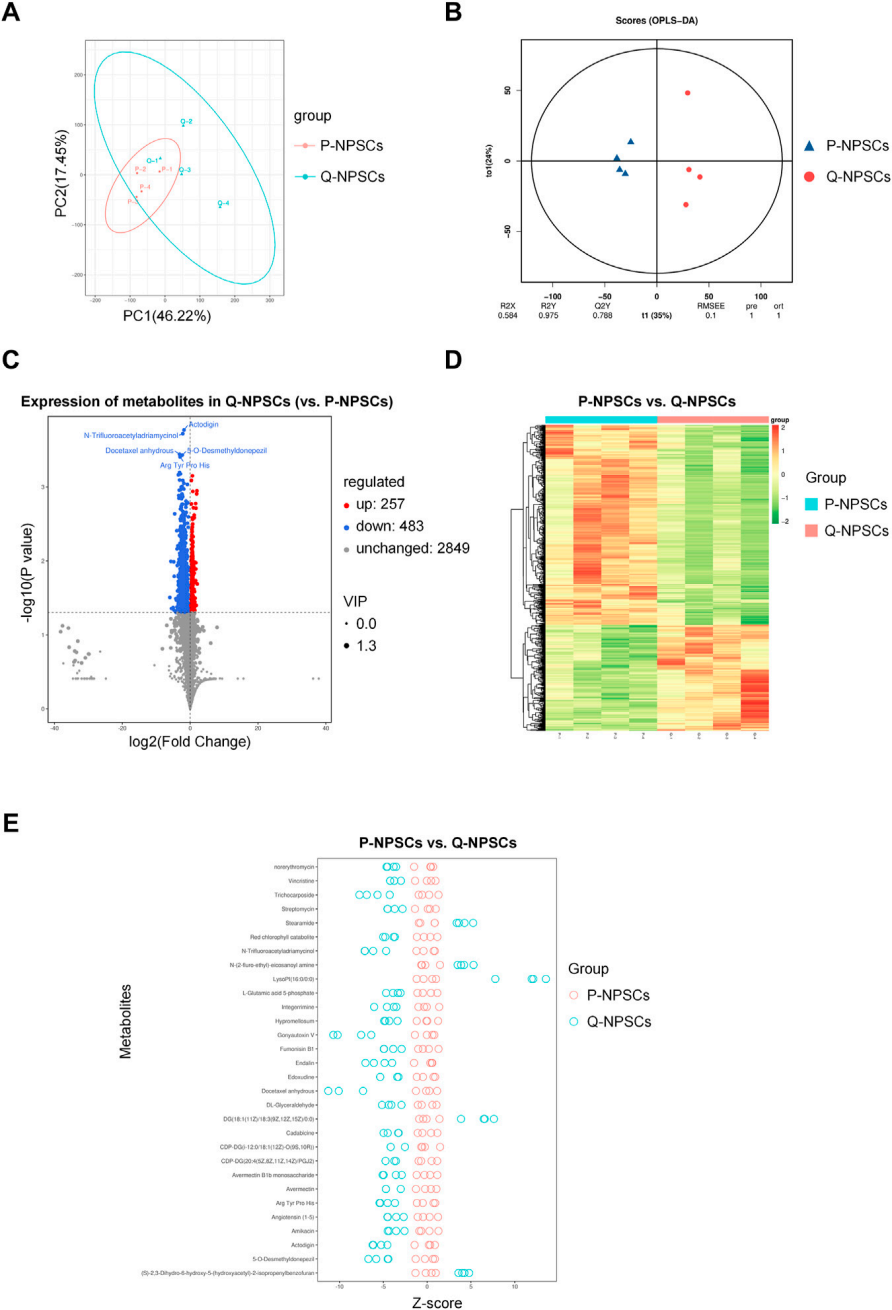
The metabolomics analysis of P-NPSCs and Q-NPSCs. **(A)** Principal component analysis (PCA) revealed the metabolic differences between P-NPSCs and Q-NPSCs. **(B)** The permutation test was performed by the orthogonal partial least squares discriminant analysis (OPLS-DA). **(C,D)** The differential metabolites of P-NPSCs and Q-NPSCs were analyzed by cluster analysis and represented by volcano plots **(C)** and heat map **(D)**. The screening criteria were Fold Change (FC) > *p*-value <0.05 and Variable Importance in Projection (VIP) > 1. The scatter size represents the VIP value of the OPLS-DA model. The labeled differential metabolites in the volcano represent the top five differential metabolites ranked by *p*-value. **(E)** The difference deviation in the P-NPSCs and Q-NPSCs were analyzed using the Z-score. A positive value means up, while a negative value means down. The top 30 differential metabolites ranked by *p*-value are shown in **(E)**.

Using the liquid chromatography-quaternary time-of-flight mass spectrometry (LC-QTOF) technology, qualitative and quantitative analyses were performed for metabolomics on the eight samples of P-NPSCs and Q-NPSCs, and a total of 14,809 peaks were detected (Supplementary Figure S1, Supplementary File S1). A total of 3,589 metabolites were annotated using the online METLIN database and Biomark’s self-built library. Principal component analysis (PCA) showed considerable differences between P-NPSCs and Q-NPSCs (Figure 6A, Supplementary File S2). Orthogonal partial least squares discriminant analysis (OPLS-DA) was performed to assess the reliability of the data model. The Q2Y score of the OPLS-DA represents the predictive ability of the model, that is, whether the model can distinguish correct sample groups by metabolic expression. The closer the R2Y and Q2Y of the index are to 1, the more stable and reliable the model is, that is, the model can be used to screen differential metabolites. The model was considered effective when Q2Y > 0.5 and excellent when Q2Y > 0.9. This model has a Q2Y value of 0.788 (Figure 6B). Then 740 differential metabolites were screened out according to Fold Change (FC), *p*-value, and Variable Importance in Projection (VIP) obtained by the OPLS-DA. Differences were considered significant if FC ≥ 1, *p*-value <0.05 and VIP ≥1. Compared with the P-NPSCs, the overall metabolic level of the Q-NPSC group was reduced, with 483 downregulated and 257 upregulated metabolites. The expression levels of differential metabolites in the two groups were represented by volcano and heatmap, as shown in Figures 6C, D and Supplementary File S3 respectively. The Z-score (standard score) value, a value converted based on the quantitative value of metabolites, was used to measure the difference deviation between the experimental and control groups. It should be noted that the Z-scores are calculated based on the mean and standard deviation of the control group (P-NPSCs), in order to center the control group on the axis for easy comparison. A positive value means up, while a negative value means down. The top 30 differential metabolites ranked by *p*-value are shown in Figure 6E (Supplementary File S4).

**FIGURE 7:**
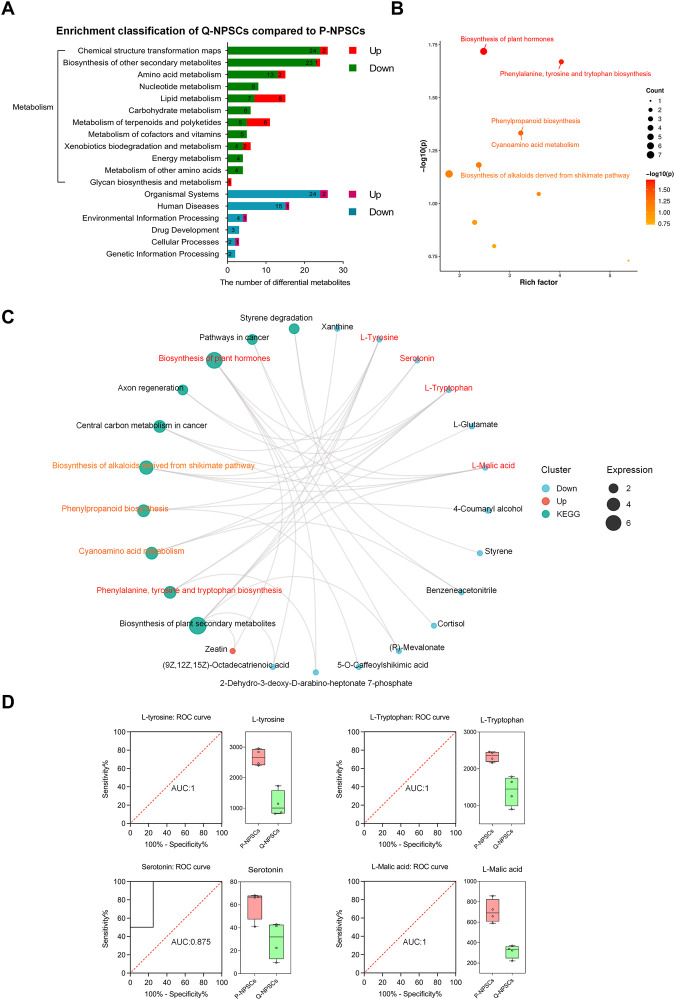
The pathway enrichment analysis of differential metabolites between P-NPSCs and Q-NPSCs. **(A)** Metabolic pathway and other pathways were analyzed with reference to the KEGG database. **(B)** Bubble map of the KEGG enrichment factor for differential metabolites. The *X*-axis is the enrichment factor and the *Y*-axis is the *p*-value. The size of the dots represents the number of differential metabolites enriched. The labeled dots are the top five metabolic pathways ranked by *p*-value and were used for the analysis; The unlabeled dots were not used. **(C,D)** Several representative differential metabolites of Q-NPSCs compared to P-NPSCs were obtained by enrichment network diagram **(C)** and analyzed by the receiver operating characteristic (ROC) curves and box plots **(D)**.

Next, a pathway enrichment analysis was performed using the KEGG database for these differential metabolites (Figures 7A, 7B, Supplementary Files S5, S6). Considerable differences were observed in the following metabolic pathways: phenylalanine, tyrosine, and tryptophan biosynthesis, plant hormone biosynthesis, cyanoamino acid metabolism, and phenylpropanoid biosynthesis. Then, by analyzing the pathway network map (Figure 7C), it was found that L-tyrosine, L-tryptophan, Serotonin, and L-malic acid were enriched into multiple metabolic pathways, indicating that they play a role in these different pathways. The ROC curves were used to analyze the possibility of these four differential metabolites as specific markers of the quiescent state of NPSCs (Figure 7D). The AUC (area under the curve) is a very useful measure for measuring the ROC curve. It is always between 0.5 and 1.0. The closer the AUC is to 1, the more different the substance is from the control and experimental groups (i.e., a potential biomarker). L-tyrosine, L-tryptophan and L-malic acid have an AUC of 1, and Serotonin has an AUC of 0.875. The boxplots showed that the expressions of L-tyrosine, L-tryptophan, Serotonin, and L-malic acid in Q-NPSCs were significantly lower than those in P-NPSCs. These results showed that the Q-NPSC group showed distinct metabolic patterns that were significantly different from those in the P-NPSC group. Furthermore, it may be possible to find biomarkers for the quiescent state of the NPSCs from a metabolic perspective, and a large sample size is required for verification.

## 4 Discussion

**FIGURE 8:**
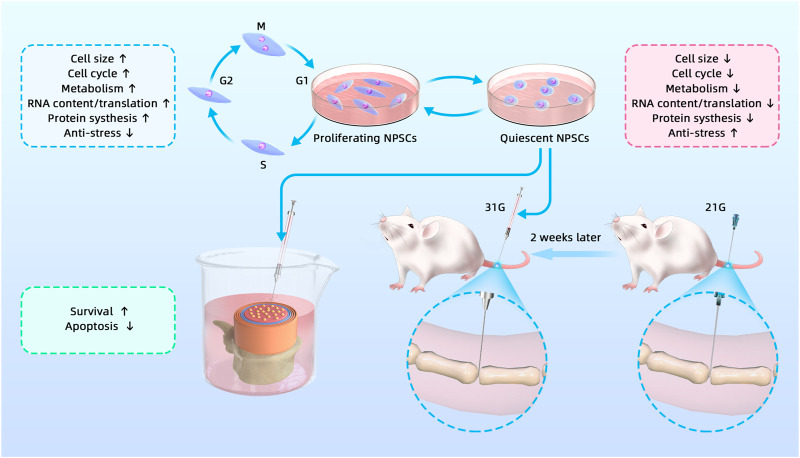
Graphical abstract of the study, including *in vivo* and *in vitro* experiments.

Stem cell transplantation for IDD treatment is a hot topic in current research.​ ​NPSCs have become one of the ideal seed cells because of their unique advantages. However, stem cells used for transplantation are generally in the proliferative phase, which is inconsistent with the quiescence of adult stem cells *in vivo*. Moreover, we have previously confirmed the quiescence of NPSCs *in vitro* (Li et al., 2019). Theoretically, the transplantation of Q-NPSCs would be more suitable for the internal environment of the disc. In addition, quiescent stem cells can resist adverse environments to enhance their survival. Moya et al. showed that preconditioned quiescent human MSCs could survive better *in vitro* under hypoxia and glucose-free conditions (Moya et al., 2017). In view of the special ND microenvironment in the IVD, we hypothesized that the transplantation of Q-NPSCs could better preserve survival and cellular biological functions, facilitating the repair of the degenerated disc. As shown in Figure 8, we tested this hypothesis through *in vivo* and *in vitro* experiments.

First, we successfully isolated NPSCs from rat NP tissues and identified their multilineage differentiation ability and immunophenotypes. As shown in Figure 1, NPSCs have good growth activity, high purity, and strong differentiation ability, and they are highly potential seed cells for repairing IDD. Then we induced these NPSCs to enter the quiescent phase by serum starvation pretreatment for 48 h. As shown in Figure 2, the vast majority of quiescence-inducing NPSCs were in the G0 phase and showed low or no proliferation. The mRNA levels of PCNA and Ki67 were decreased. Compared with the proliferating NPSCs, the mRNA levels of Sox9 and Acan were also decreased in the quiescent NPSCs. Interestingly, Col2a1 mRNA level was significantly increased. The secretion of ECM was increased in quiescent cells, potentially reflecting the preservation of essential physiological functions of the cells in response to harsh environments (Lemons et al., 2010). In addition, we also observed Ki67 level by cellular immunofluorescence. Similar to the mRNA level, Ki67 level was significantly decreased in the Q-NPSCs. However, when the cells were reactivated, the proliferative activity was immediately restored, and the mRNA levels of PCNA, Ki67, Sox9, and Acan were significantly restored or increased, and the mRNA level of Col2a1 was high. Furthermore, Ki67 level was restored in the reactivated NPSCs. These results suggest that Q-NPSCs maintain the potential to proliferate, differentiate, and synthesize ECM and may even perform better after reactivation.

As shown in Figures 2D,E,H, Re-NPSCs outperformed P-NPSCs in the expression of Sox-9, Acan, and Col2a1, and were comparable to P-NPSCs in cell viability, but slightly inferior to P-NPSCs in the expression of PCNA and Ki67 and in the cell cycle. This involves the activation mechanism of quiescent stem cells. Urbán et al. (Urban and Cheung, 2021) suggested that when stem cells enter a deep quiescent state, it takes longer to recover to a proliferative state, which is influenced by various factors. ​Furthermore, due to the drawbacks of inducing stem cells into a quiescent state by means of serum starvation, it leads to a degree of apoptosis or irreversible senescence (G0) (Marescal and Cheeseman, 2020). These ideas suggest it takes a longer time for stem cells to recover from quiescence and that some degree of cell loss occurs are well made. This is a problem that researchers are eager to address and look forward to more satisfactory induction methods in the future. In conclusion, our data suggest that NPSCs regain proliferation and biological functions to levels comparable with P-NPSCs. We believe that as technology advances and difficulties are resolved in the future, quiescence preconditioned NPSCs are expected to become a new transplantation strategy for repairing disc degeneration.

Studies have shown that quiescence enables stem cells to withstand harsh environments and reduces cell death (Stuart and Brown, 2006; Alekseenko et al., 2018; Ferro et al., 2019). P- NPSCs and Q-NPSCs were cultured in a serum-free and glucose-free environment to investigate their survival under ND conditions *in vitro*. As shown in Figure 3, the results showed that Q-NPSCs showed a higher survival rate and anti-apoptotic ability than P-NPSCs in response to the ND environment. Subsequently, when the two types of NPSCs were implanted into the IVD, a similar phenomenon was observed; the Q-NPSCs survived more, as shown in Figure 4. On the seventh day of cell transplantation, the survival rate of these P-NPSCs decreased to 30%, whereas that of the Q-NPSCs remained above 60%. This result indicates that Q-NPSCs have better tolerance to the ND environment than P-NPSCs, which contributes to cell survival.

Cell therapy of the IVD depends on the survival of transplanted cells and their biological functions (Sakai and Grad, 2015; Tong et al., 2017). In this study, we constructed a rat model of IDD by needle puncture injury. We transplanted quiescent NPSCs, proliferating NPSCs, and PBS into the IVD of the rat IDD model and evaluated the repair efficacy of each group by radiography, histology, and ECM synthesis. The results are shown in Figure 5. The disc segment degeneration was most obvious in PBS injection only. In contrast, the degeneration of the segments transplanted with either quiescent or proliferating NPSCs was significantly improved. However, Q-NPSC transplantation showed lower disc height reduction, lower histological scores, and better ECM synthesis than P-NPSC transplantation. Therefore, quiescence preconditioning can improve the effectiveness of NPSCs in IDD repair.

Although many previous studies have reported cell therapy for IDD, ensuring cell survival after transplantation is difficult. In this study, we present an innovative treatment strategy for the quiescence preconditioning of NPSCs. This approach was experimentally evaluated and successfully demonstrated to improve the survival and repair ability of NPSCs, both *in vitro* and *in vivo*. However, due to the lack of vertical stress on the spine, the rat model of IDD constructed by needle puncture was not completely consistent with the IDD process in humans (Han et al., 2008). Therefore, performing experiments on large animals, such as rhesus monkeys (Zhou et al., 2013), is necessary to verify the repair ability of quiescent NPSCs further.

However, one of the most important questions will be to determine whether engrafted cells survive over the long term and directly contribute to disc formation, or if they survive only transiently, but in a manner that is sufficient to stimulate a repair from within the endogenous environment. According to previous studies (Clouet et al., 2019; Lyu et al., 2019), there must be endogenous cellular repair during disc degeneration. In our study, injected segments significantly delayed the disc degeneration compared to segments without injected cells, indicating that exogenous NPSCs play an important role in repairing intervertebral disc degeneration. This may be one of the shortcomings of this study, and more information is needed for further research.

Based on the above studies, we have confirmed that Q-NPSCs are more tolerant to the harsh environment to maintain survival, but the specific mechanism still needs further exploration. Previous studies have shown that the quiescent state of stem cells is regulated by metabolism (Moya et al., 2017; Theret et al., 2017; Cho et al., 2019; Marescal and Cheeseman, 2020). Moya A et al. reported that quiescence pretreatment causes a protective metabolic adaptation to improve the survival of human MSCs in an ischemic environment (Moya et al., 2017). Theret M et al. showed that AMP-activated protein kinase, the master metabolic regulator of a cell, regulated muscle stem cells returning to quiescence, emphasizing the importance of metabolism in stem cell fate (Theret et al., 2017). Therefore, we examined the metabolic differences between the quiescent and proliferating NPSCs by metabolomics. The results are shown in Figures 6, 7. The overall metabolic level of the quiescent NPSCs was significantly reduced, including amino acid, nucleotide, carbohydrate, and energy metabolisms. Amino acid metabolism pathways (including tyrosine, tryptophan, and phenylalanine metabolisms) and nucleotide metabolism pathways (including pyrimidine and purine metabolisms) were significantly downregulated, indicating that protein anabolism was reduced, which was consistent with previous reports (Cho et al., 2019; Marescal and Cheeseman, 2020).

Further analysis of the KEGG enrichment network graph revealed several significantly under-expressed metabolites such as L-tyrosine, L-tryptophan, Serotonin, and L-malic acid, which are key metabolites in multiple enrichment pathways. L-Tyrosine is closely related to cell cycle transition (Kaldis, 2007; Jäkel et al., 2012). The cyclin-dependent kinase inhibitor P27 can be phosphorylated on tyrosine, and phosphorylated P27 reduces the ability to inhibit the activity of cyclin-dependent kinases, a mechanism that allows cells to enter the cell cycle from quiescence. In this study, we hypothesized that the low level of tyrosine in the quiescent NPSCs reduced the phosphorylation of P27, improving the stability of P27, thus inhibiting the cell cycle and allowing the NPSCs to remain in the quiescent state. Moreover, tryptophan is an essential amino acid that the body cannot synthesize; it is also an essential precursor in serotonin synthesis, which promotes proliferation (Comai et al., 2020). Thus, with the constant depletion of tryptophan in quiescent NPSCs, cell cycle arrest was induced (Mellor et al., 2003; Oh et al., 2017) and serotonin level was reduced (Fang et al., 2018; Gordeeva and Safandeev, 2021), probably contributing to NPSC quiescence. L-Malic acid is a vital organic acid produced in the metabolic process of organisms and an important intermediate in the tricarboxylic acid cycle and its branch, the glyoxylate cycle. L-Malic acid can quickly pass through the cell membrane and enter mitochondria to participate in energy metabolism directly (Ding et al., 2016); it is also an important component of the malate aspartate shuttle and is important in transporting NADH from the cytosol to mitochondria for ATP production (Scholz et al., 2000; Wu et al., 2011). In the present study, L-malic acid was reduced in the Q-NPSCs, which reduced ATP production.

However, we found that lipid, terpenoid, and polyketide metabolisms were active in the quiescent NPSCs. This is consistent with previously reported results (Lemons et al., 2010). Lipid metabolism is an important and complex biochemical reaction in the body, which refers to the process of digestion, absorption, synthesis, and decomposition of lipids in organisms under the catalysis of numerous enzymes to be processed into products needed by the body to ensure the normal operation of physiological functions and is crucial for life activities (DeBose-Boyd, 2018). Lipids are not only essential for energy supply and storage but they also play an important role as structural components of biofilms (Yao et al., 2019). Thus, ensuring basic lipid metabolism is beneficial for maintaining cellular functions and for stabilizing the membrane structure. In addition, terpenoids and polyketides stabilize cells, resist oxidation, and inhibit proliferation (de Carvalho and da Fonseca, 2006; Kotoku et al., 2017), which need to be further verified in Q-NPSCs.

## 5 Conclusion

In summary, our findings suggested that the quiescence preconditioning of the NPSCs maintained proliferative potential and cell viability, enhanced survival, and exhibited cellular functions in response to the transition to a harsh, nutrient-deficient environment, thereby facilitating IDD repair. Thus, we hypothesized that the quiescent NPSCs adopted an adaptive metabolic pattern that favored resistance to the metabolic stresses encountered. As this quiescence preconditioning method *via* serum starvation is safe, feasible, and does not require many cells, it can be a suitable strategy for using NPSCs in IDD treatment in the future.

## Data Availability

The datasets presented in this study can be found in online repositories. The names of the repository/repositories and accession number(s) can be found in the article/Supplementary Material.
