# Supplementary material for: Quiescence preconditioned nucleus pulposus stem cells alleviate intervertebral disc degeneration by enhancing cell survival via adaptive metabolism pattern in rats
Source: Front Bioeng Biotechnol. 2023 Feb 10;11:1073238. doi: 10.3389/fbioe.2023.1073238 (PMC9950514; doi:10.3389/fbioe.2023.1073238)
Supplement: Supplementary file 1 [file Table1.DOCX]

**Histological grading of the disc degeneration**

| Cellularity and Morphology | Grade |
| --- | --- |
| Cellularity of the annulus fibrosus | 1. Fibroblasts comprise more than 75% of the cells 2. Neither fibroblasts nor chondrocytes comprise more than 75% of the cells 3. Chondrocytes comprise more than 75% of the cells |
| Morphology of the annulus fibrosus | 1. Well-organized collagen lamellae without ruptured or serpentine fibers 2. Inward bulging, ruptured or serpentine fibers in less than one third of the annulus 3. Inward bulging, ruptured or serpentine fibers in more than one third of the annulus |
| Border between the annulus fibrosus and nucleus pulposus | 1. Normal, without any interruption 2. Minimal interruption 3. Moderate or severe interruption |
| Cellularity of the nucleus pulposus | 1. Normal cellularity with stellar-shaped nuclear cells evenly distributed throughout the nucleus 2. Slight decrease in the number of cells with some clustering 3. Moderate or severe decrease (>50%) in the number of cells with all the remaining cells clustered and separated by dense areas of proteoglycans |
| Morphology of the nucleus pulposus | 1. Round, comprising at least half of the disc area in mid-sagittal sections 2. Rounded or irregularly shaped, comprising one quarter to half of the disc area in mid-sagittal sections 3. Irregularly shaped, comprising less than one quarter of the disc area in mid-sagittal sections |

**Reference：**

Zhou X, Wang J, Fang W, et al. Genipin cross-linked type II collagen/chondroitin sulfate composite hydrogel-like cell delivery system induces differentiation of adipose-derived stem cells and regenerates degenerated nucleus pulposus. Acta Biomater. 2018;71:496-509.
