# Supplementary material for: Quiescence preconditioned nucleus pulposus stem cells alleviate intervertebral disc degeneration by enhancing cell survival via adaptive metabolism pattern in rats
Source: Front Bioeng Biotechnol. 2023 Feb 10;11:1073238. doi: 10.3389/fbioe.2023.1073238 (PMC9950514; doi:10.3389/fbioe.2023.1073238)
Supplement: Supplementary file 2 [file DataSheet1.zip › Supplementary Figure S1_raw data/neg-total.pdf]

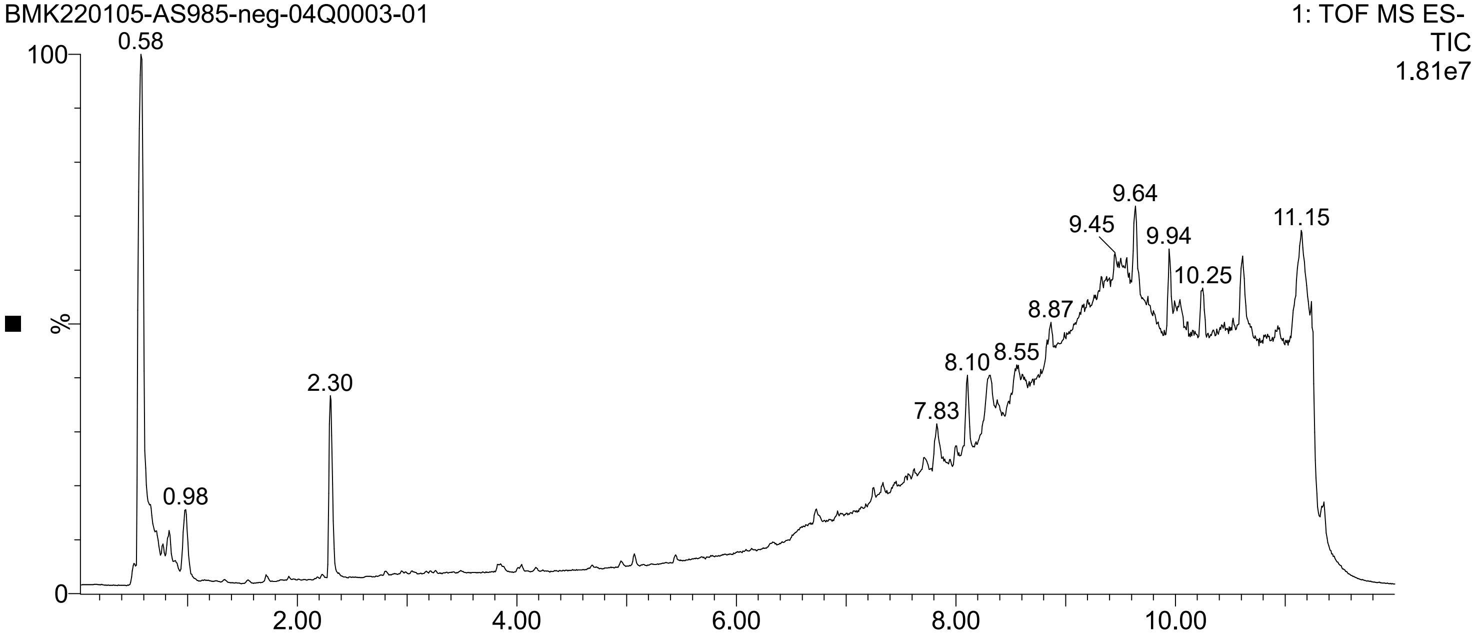
BMK220105-AS985-neg-04Q0003-01
1: TOF MS ES-
TIC
1.81e7
100
%
0
0.58
0.98
2.30
7.83
8.10
8.55
8.87
9.45
9.64
9.94
10.25
11.15
2.00
4.00
6.00
8.00
10.00

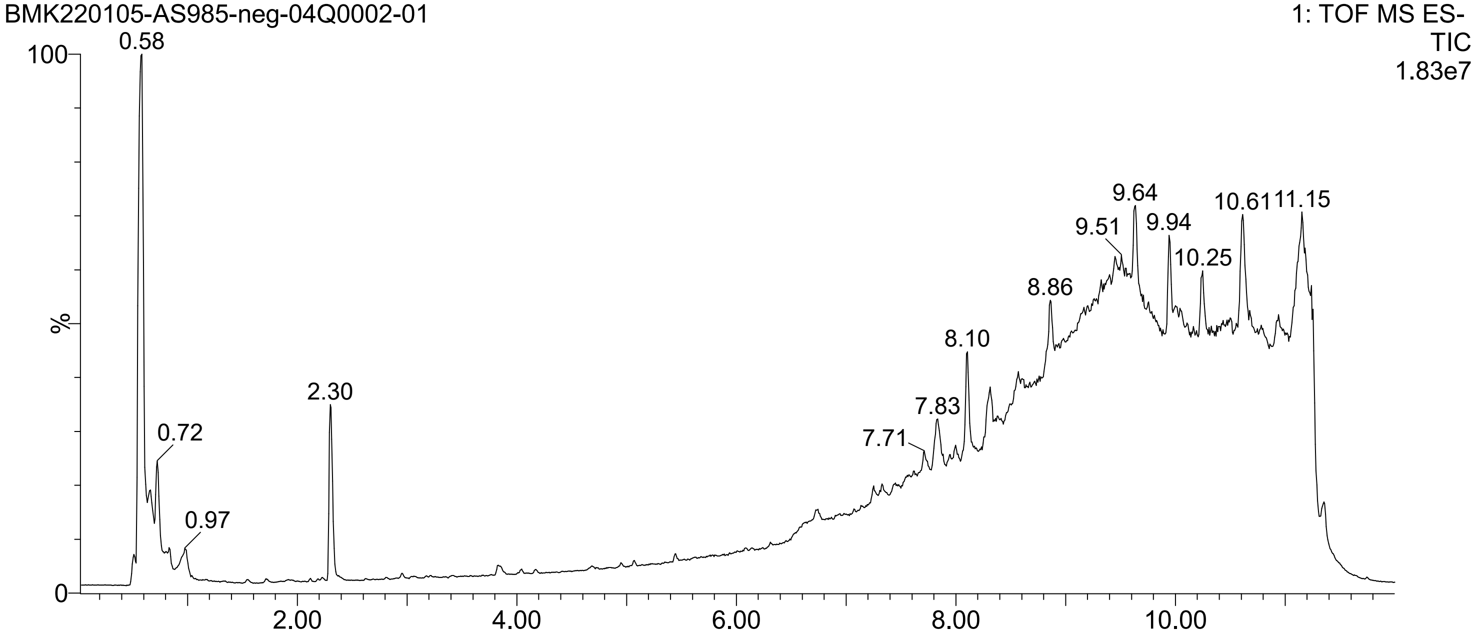
BMK220105-AS985-neg-04Q0002-01
1: TOF MS ES-
TIC
1.83e7
100
%
0
0.58
0.72
0.97
2.30
7.71
7.83
8.10
8.86
9.51
9.64
9.94
10.25
10.61
11.15
2.00
4.00
6.00
8.00
10.00

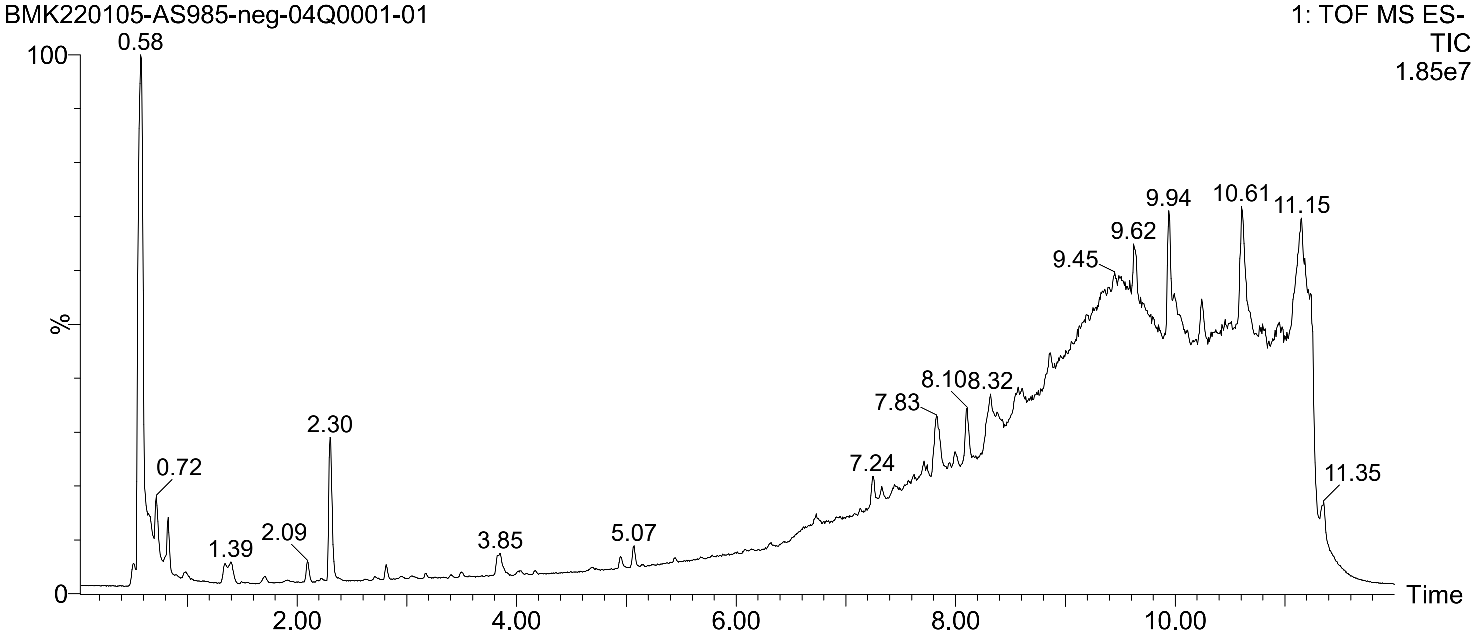
BMK220105-AS985-neg-04Q0001-01
1: TOF MS ES-
TIC
1.85e7
100
%
0
0.58
0.72
1.39
2.09
2.30
3.85
5.07
7.24
7.83
8.10
8.32
9.45
9.62
9.94
10.61
11.15
11.35
Time
2.00
4.00
6.00
8.00
10.00

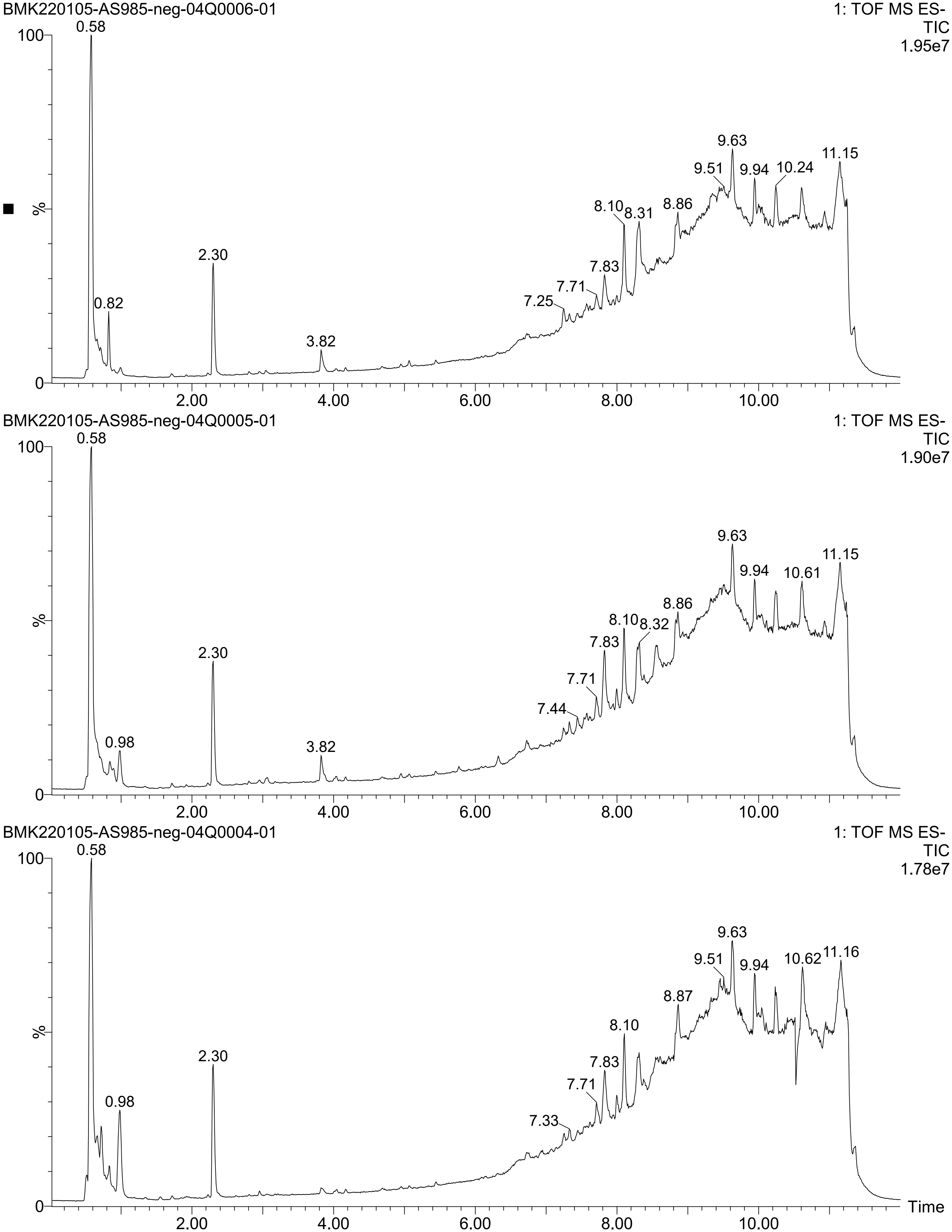
BMK220105-AS985-neg-04Q0006-01
1: TOF MS ES-
TIC
1.95e7
0.58
0.82
2.30
3.82
7.25
7.71
7.83
8.10
8.31
8.86
9.51
9.63
9.94
10.24
11.15
100
%
0
2.00
4.00
6.00
8.00
10.00
BMK220105-AS985-neg-04Q0005-01
1: TOF MS ES-
TIC
1.90e7
0.58
0.98
2.30
3.82
7.44
7.71
7.83
8.10
8.32
8.86
9.63
9.94
10.61
11.15
100
%
0
2.00
4.00
6.00
8.00
10.00
BMK220105-AS985-neg-04Q0004-01
1: TOF MS ES-
TIC
1.78e7
0.58
0.98
2.30
7.33
7.71
7.83
8.10
8.87
9.51
9.63
9.94
10.62
11.16
100
%
0
2.00
4.00
6.00
8.00
10.00
Time

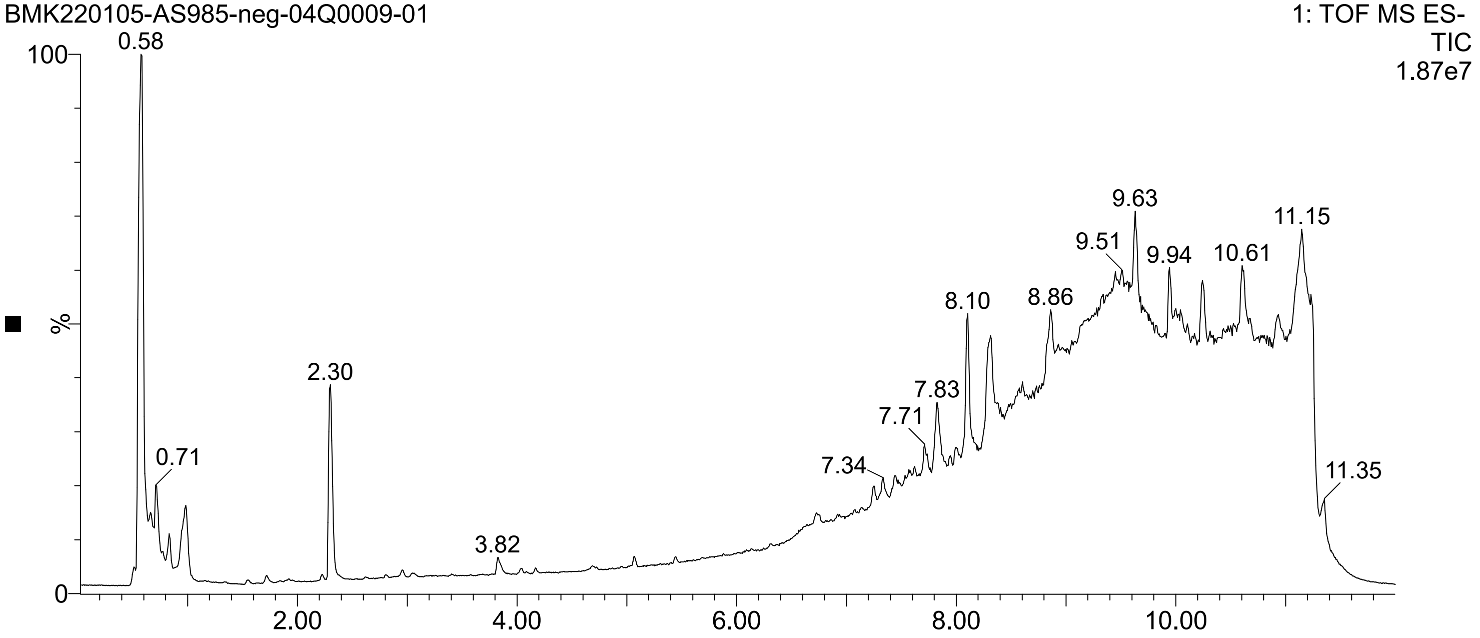
BMK220105-AS985-neg-04Q0009-01
1: TOF MS ES-
TIC
1.87e7
100
%
0
0.58
0.71
2.30
3.82
7.34
7.71
7.83
8.10
8.86
9.51
9.63
9.94
10.61
11.15
11.35
2.00
4.00
6.00
8.00
10.00

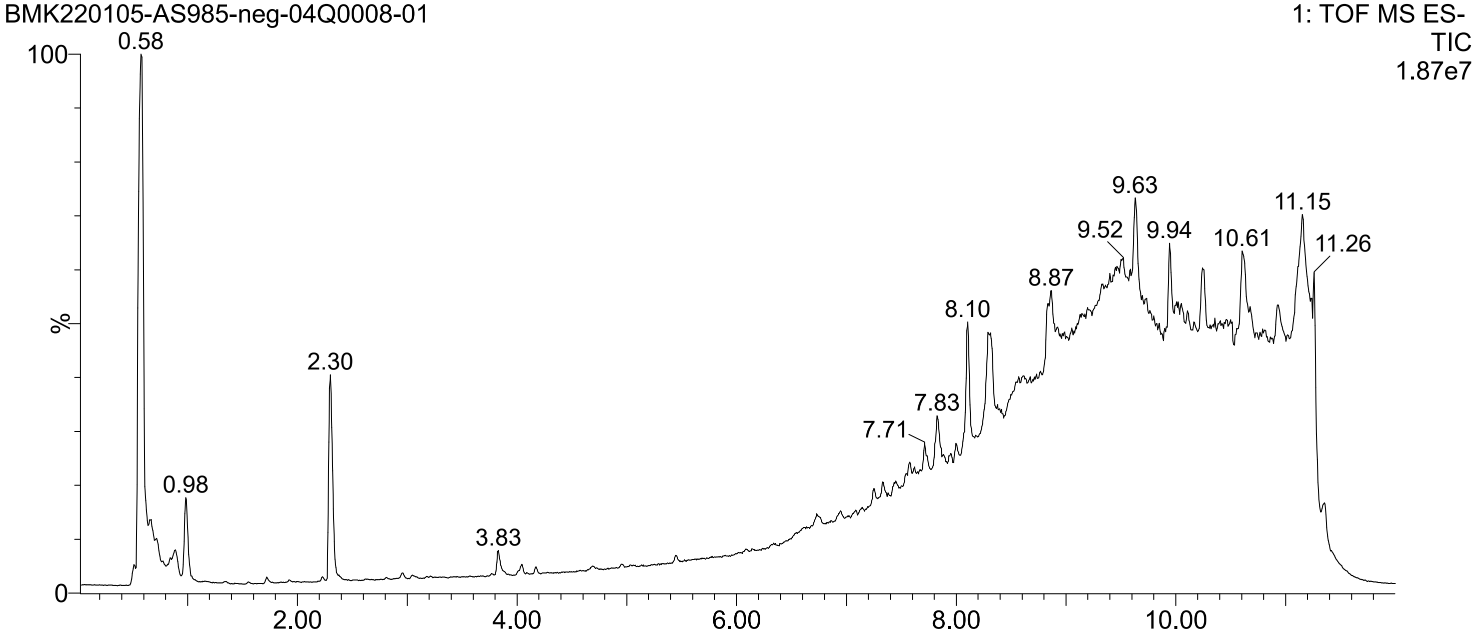
BMK220105-AS985-neg-04Q0008-01
1: TOF MS ES-
TIC
1.87e7
100
%
0
0.58
0.98
2.30
3.83
7.71
7.83
8.10
8.87
9.52
9.63
9.94
10.61
11.15
11.26
2.00
4.00
6.00
8.00
10.00

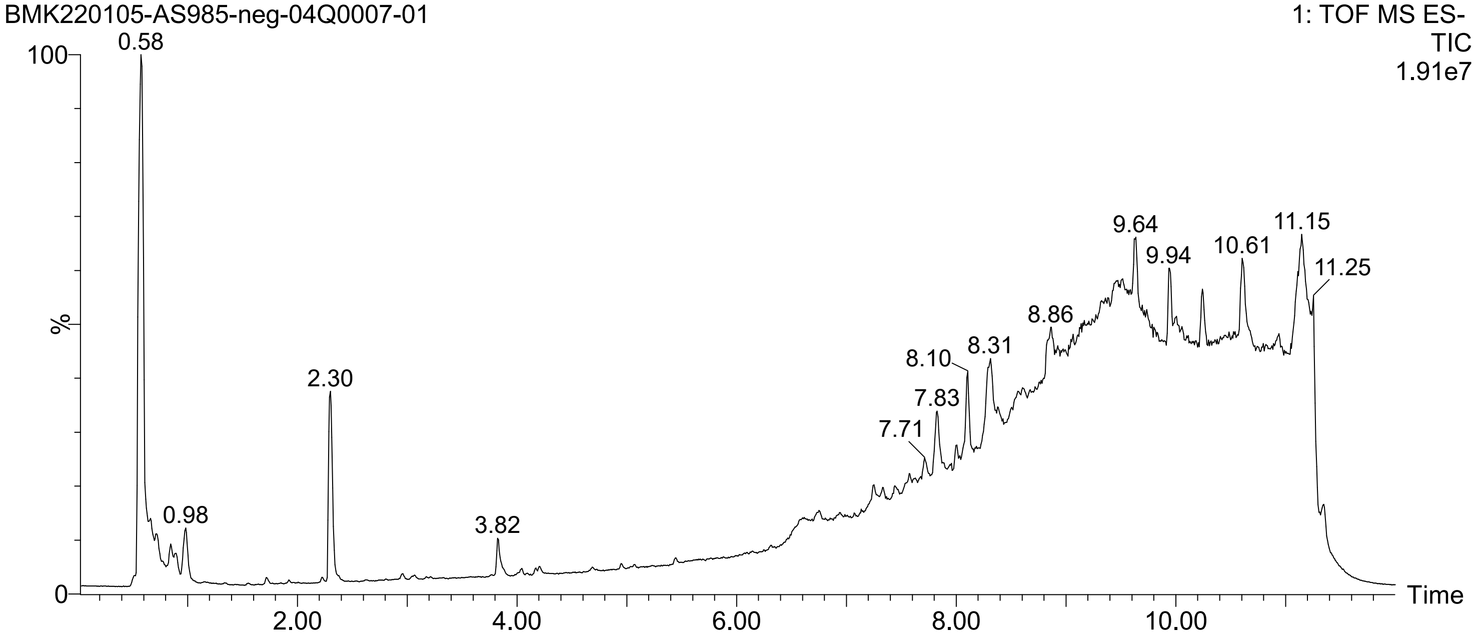
BMK220105-AS985-neg-04Q0007-01
1: TOF MS ES-
TIC
1.91e7
100
%
0
0.58
0.98
2.30
3.82
7.71
7.83
8.10
8.31
8.86
9.64
9.94
10.61
11.15
11.25
2.00
4.00
6.00
8.00
10.00
Time

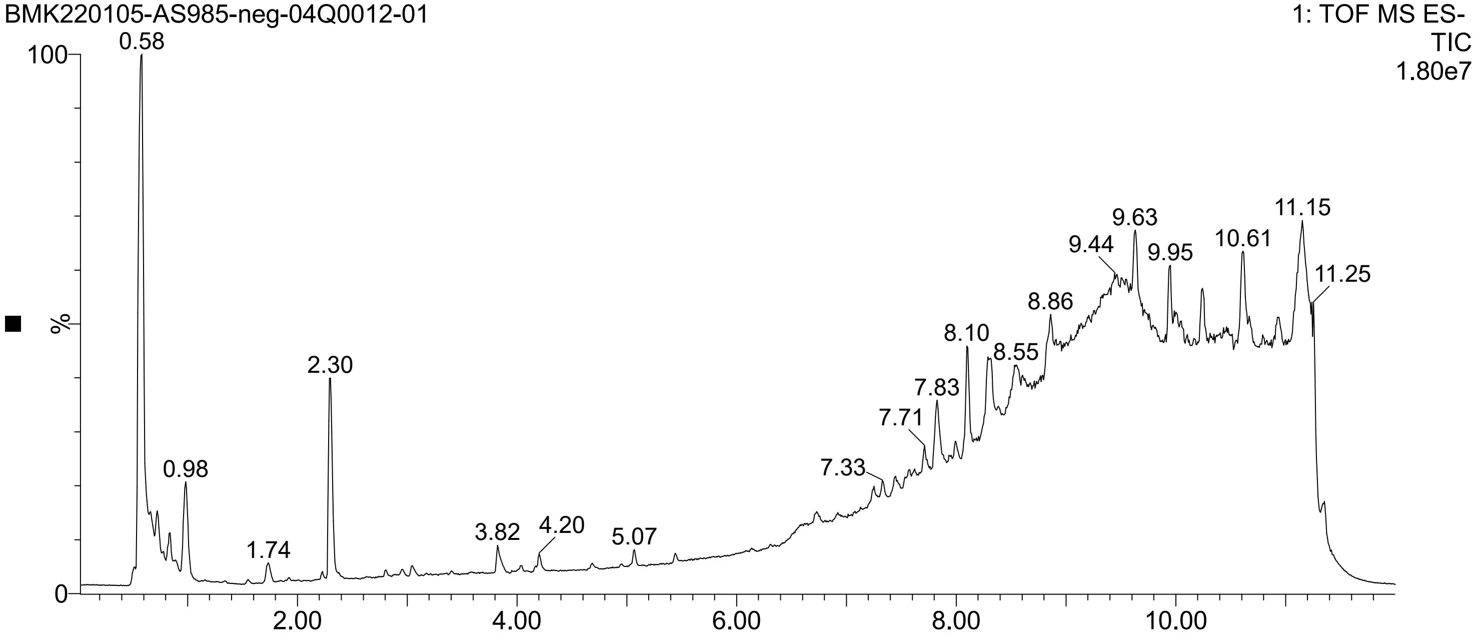
BMK220105-AS985-neg-04Q0012-01
1: TOF MS ES-
TIC
1.80e7
100
%
0
0.58
0.98
1.74
2.30
3.82
4.20
5.07
7.33
7.71
7.83
8.10
8.55
8.86
9.44
9.63
9.95
10.61
11.15
11.25
2.00
4.00
6.00
8.00
10.00

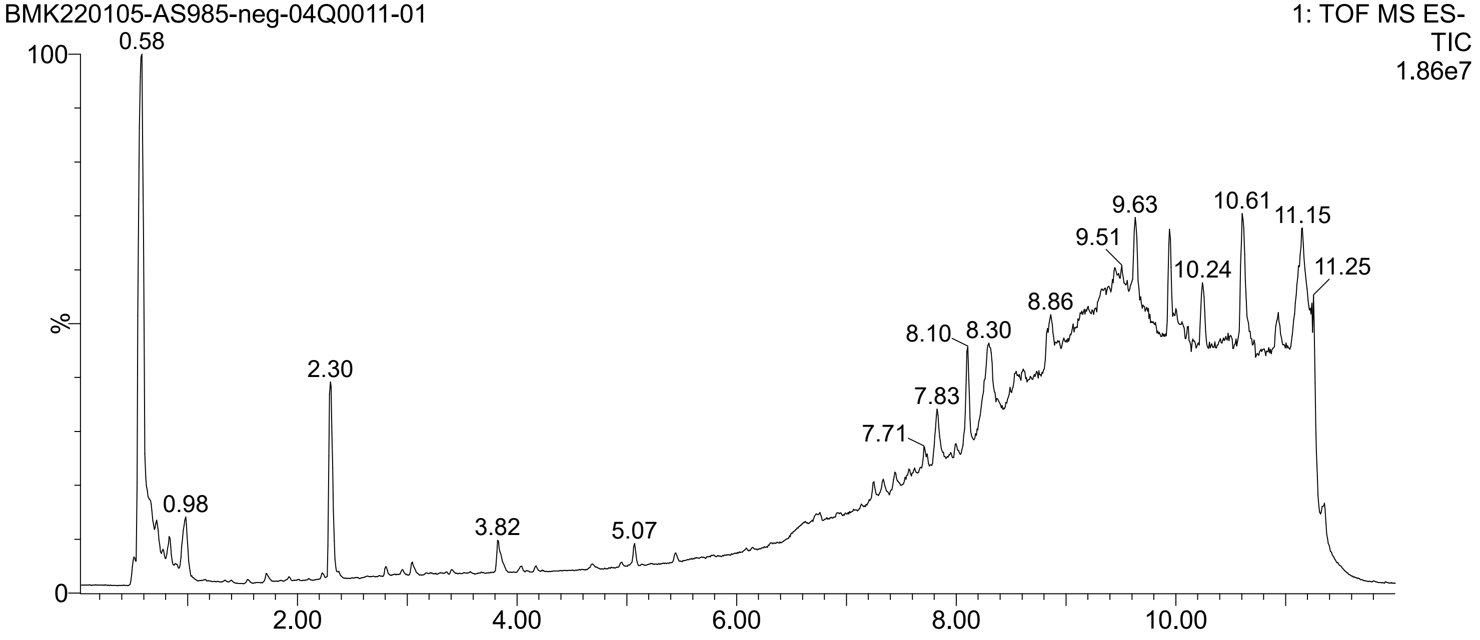
BMK220105-AS985-neg-04Q0011-01
1: TOF MS ES-
TIC
1.86e7
100
%
0
0.58
0.98
2.30
3.82
5.07
7.71
7.83
8.10
8.30
8.86
9.51
9.63
10.24
10.61
11.15
11.25
2.00
4.00
6.00
8.00
10.00

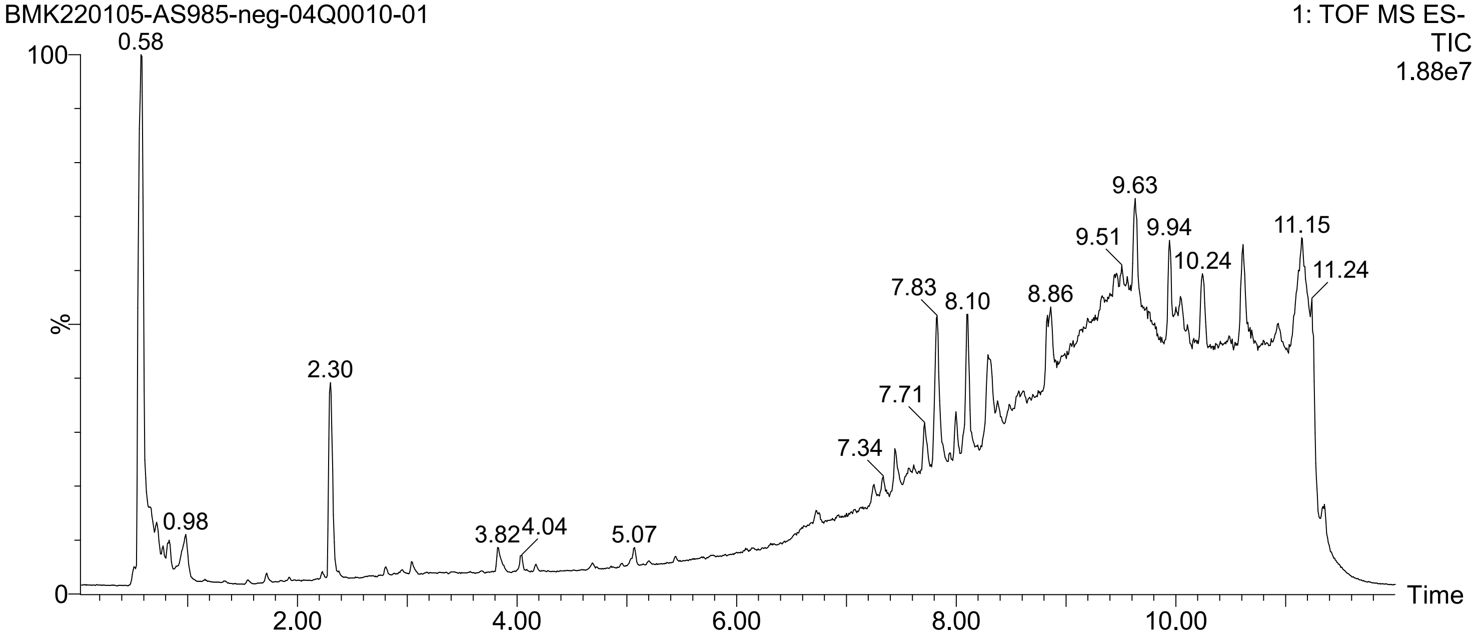
BMK220105-AS985-neg-04Q0010-01
1: TOF MS ES-
TIC
1.88e7
100
%
0
0.58
0.98
2.30
3.82
4.04
5.07
7.34
7.71
7.83
8.10
8.86
9.51
9.63
9.94
10.24
11.15
11.24
2.00
4.00
6.00
8.00
10.00
Time

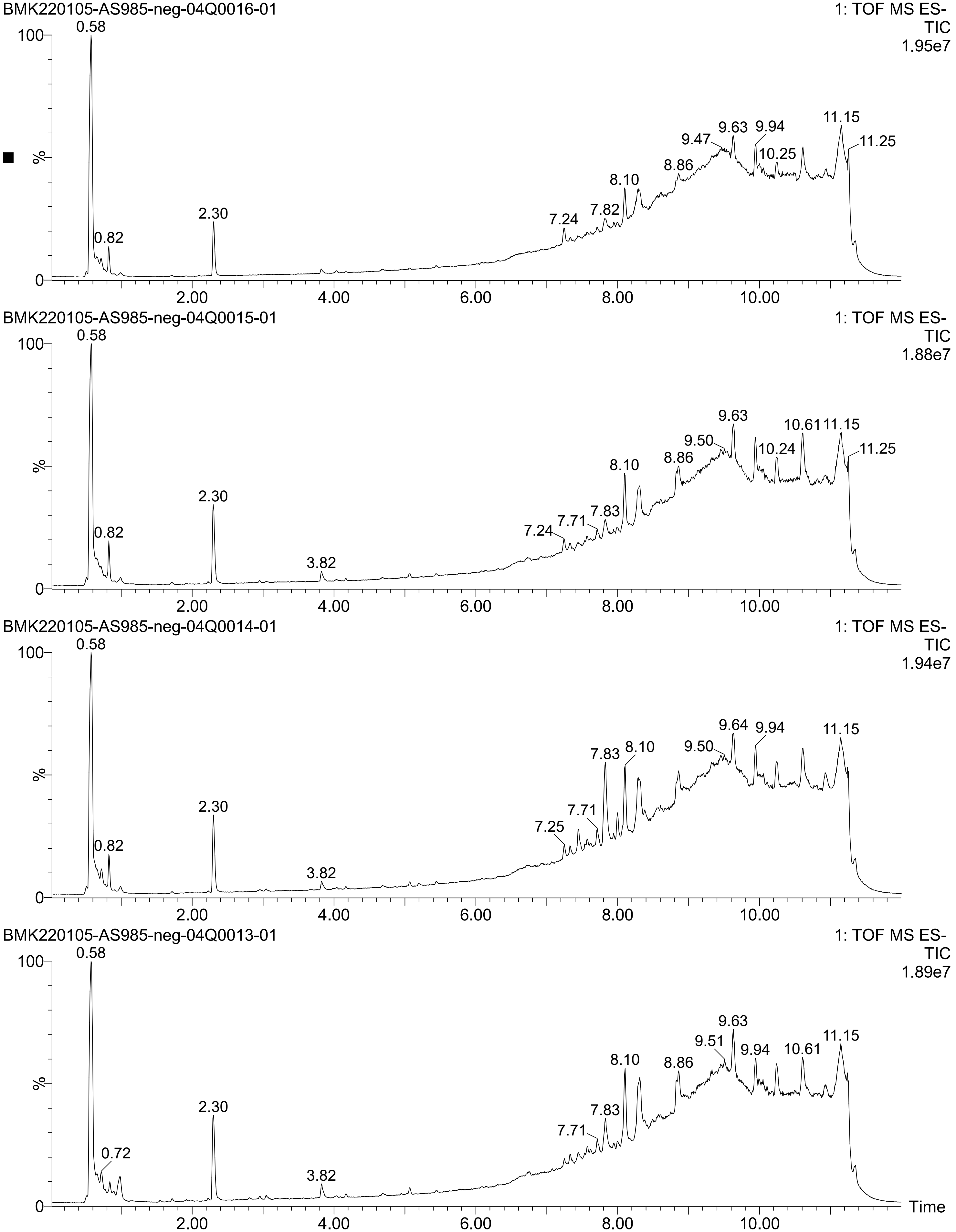
BMK220105-AS985-neg-04Q0016-01
1: TOF MS ES-
TIC
1.95e7
0.58
0.82
2.30
7.24
7.82
8.10
8.86
9.47
9.63
9.94
10.25
11.15
11.25
BMK220105-AS985-neg-04Q0015-01
1: TOF MS ES-
TIC
1.88e7
0.58
0.82
2.30
3.82
7.24
7.71
7.83
8.10
8.86
9.50
9.63
10.24
10.61
11.15
11.25
BMK220105-AS985-neg-04Q0014-01
1: TOF MS ES-
TIC
1.94e7
0.58
0.82
2.30
3.82
7.25
7.71
7.83
8.10
9.50
9.64
9.94
11.15
BMK220105-AS985-neg-04Q0013-01
1: TOF MS ES-
TIC
1.89e7
0.58
0.72
2.30
3.82
7.71
7.83
8.10
8.86
9.51
9.63
9.94
10.61
11.15
100
%
0
2.00
4.00
6.00
8.00
10.00
Time

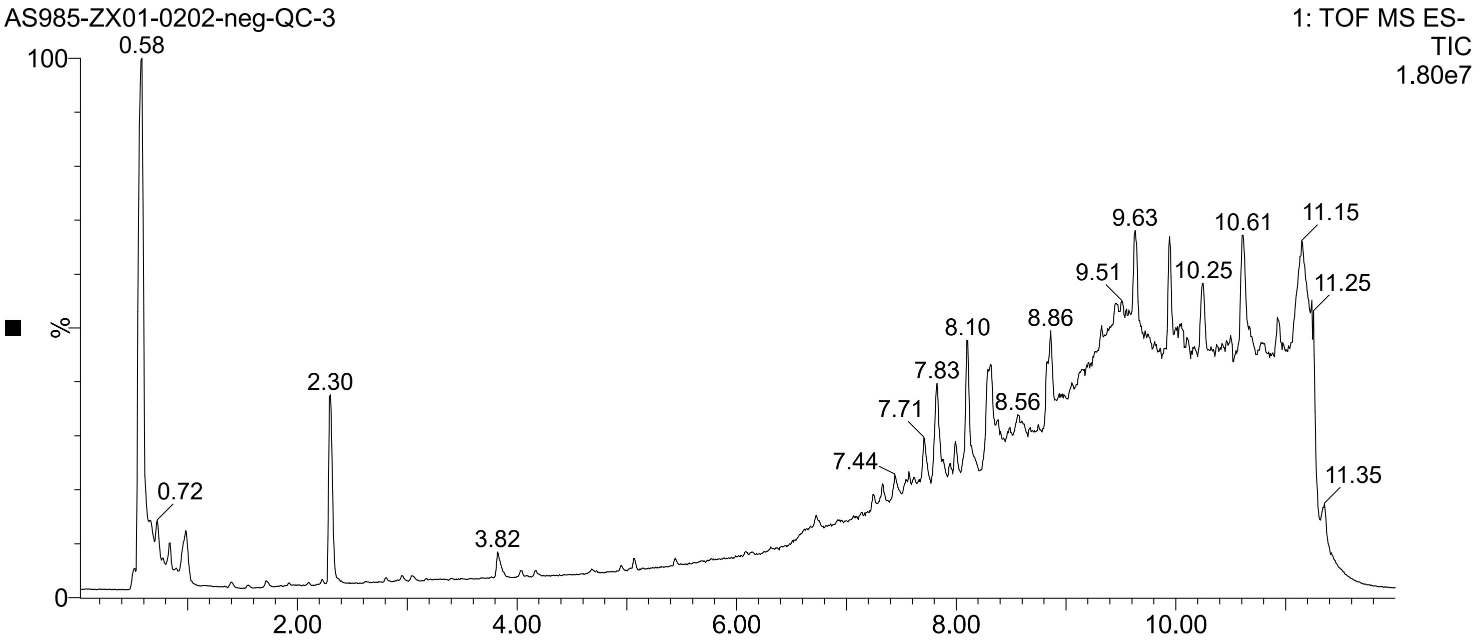
AS985-ZX01-0202-neg-QC-3
1: TOF MS ES-
TIC
1.80e7
0.58
0.72
2.30
3.82
7.44
7.71
7.83
8.10
8.56
8.86
9.51
9.63
10.25
10.61
11.15
11.25
11.35
100
%
0
2.00
4.00
6.00
8.00
10.00

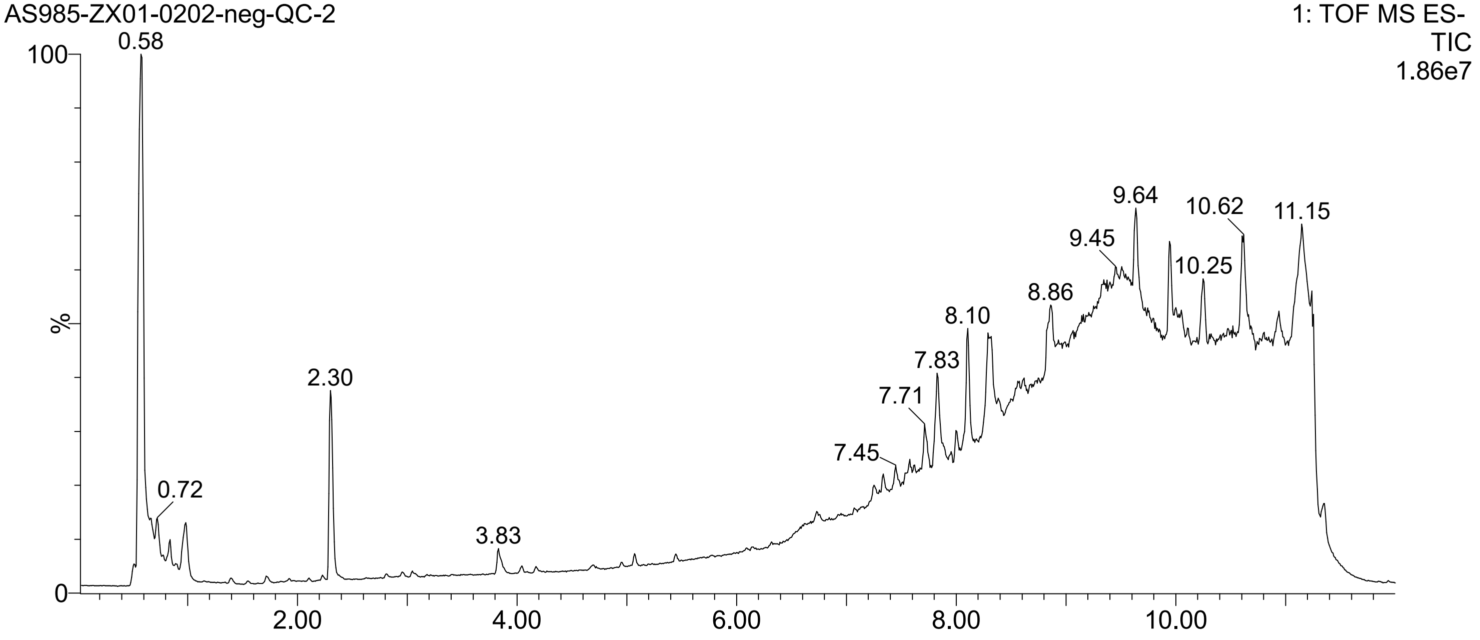
AS985-ZX01-0202-neg-QC-2
1: TOF MS ES-
TIC
1.86e7
0.58
0.72
2.30
3.83
7.45
7.71
7.83
8.10
8.86
9.45
9.64
10.25
10.62
11.15
100
%
0
2.00
4.00
6.00
8.00
10.00

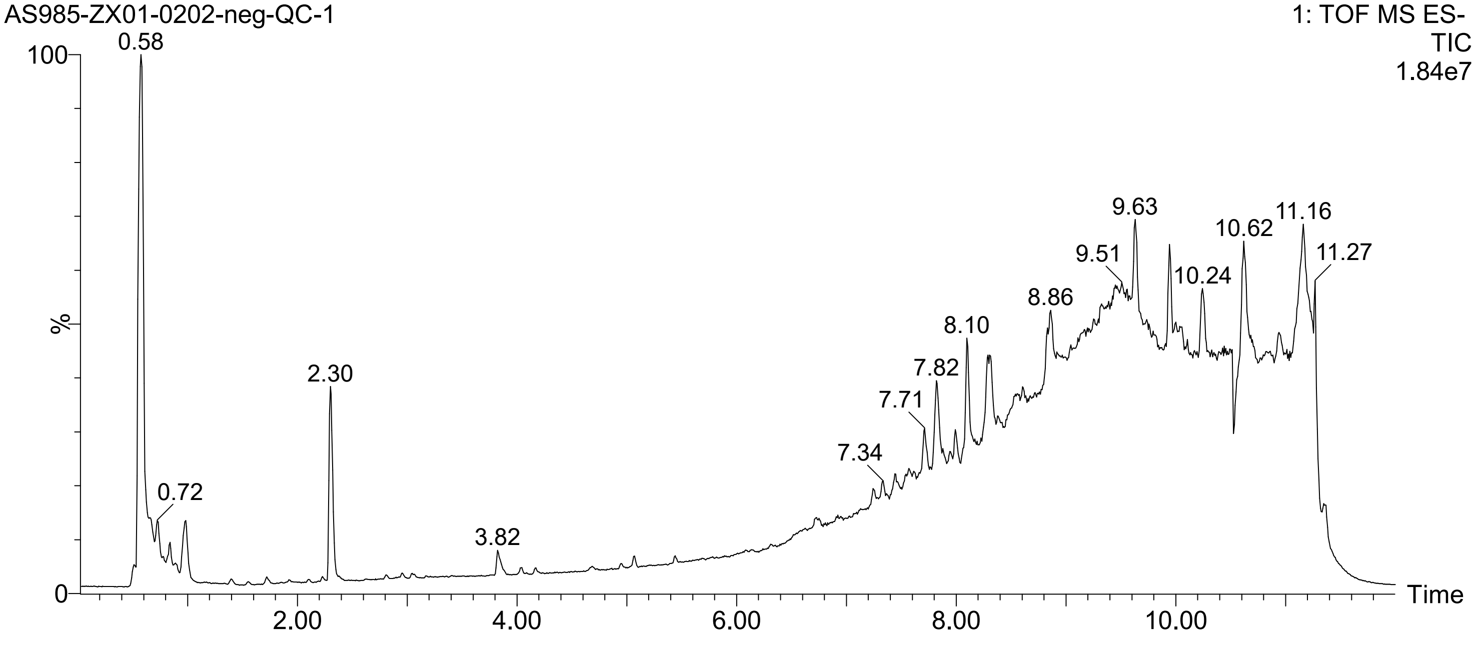
AS985-ZX01-0202-neg-QC-1
1: TOF MS ES-
TIC
1.84e7
0.58
0.72
2.30
3.82
7.34
7.71
7.82
8.10
8.86
9.51
9.63
10.24
10.62
11.16
11.27
100
%
0
Time
2.00
4.00
6.00
8.00
10.00
