# Supplementary material for: Quiescence preconditioned nucleus pulposus stem cells alleviate intervertebral disc degeneration by enhancing cell survival via adaptive metabolism pattern in rats
Source: Front Bioeng Biotechnol. 2023 Feb 10;11:1073238. doi: 10.3389/fbioe.2023.1073238 (PMC9950514; doi:10.3389/fbioe.2023.1073238)
Supplement: Supplementary file 2 [file DataSheet1.zip › Supplementary Figure S1_raw data/pos-total.pdf]

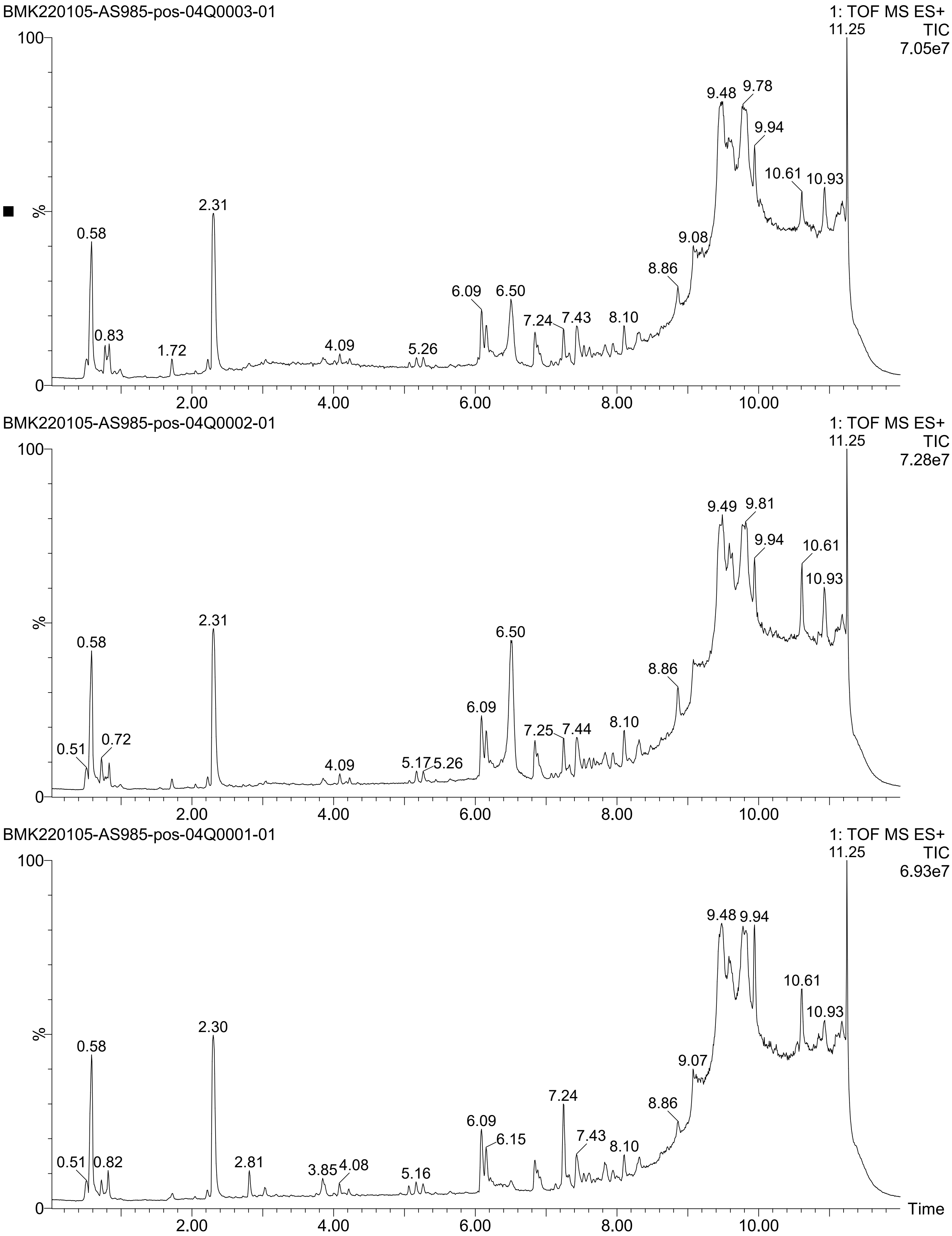
BMK220105-AS985-pos-04Q0003-01
1: TOF MS ES+
TIC
7.05e7
11.25
9.48
9.78
9.94
10.61
10.93
9.08
8.86
6.09
6.50
7.24
7.43
8.10
2.31
0.58
0.83
1.72
4.09
5.26
100
%
0
2.00
4.00
6.00
8.00
10.00
BMK220105-AS985-pos-04Q0002-01
1: TOF MS ES+
TIC
7.28e7
11.25
9.49
9.81
9.94
10.61
10.93
2.31
6.50
0.58
8.86
6.09
7.25
7.44
8.10
0.51
0.72
4.09
5.17
5.26
100
%
0
2.00
4.00
6.00
8.00
10.00
BMK220105-AS985-pos-04Q0001-01
1: TOF MS ES+
TIC
6.93e7
11.25
9.48
9.94
10.61
10.93
2.30
0.58
9.07
7.24
8.86
6.09
6.15
7.43
8.10
0.51
0.82
2.81
3.85
4.08
5.16
100
%
0
2.00
4.00
6.00
8.00
10.00
Time

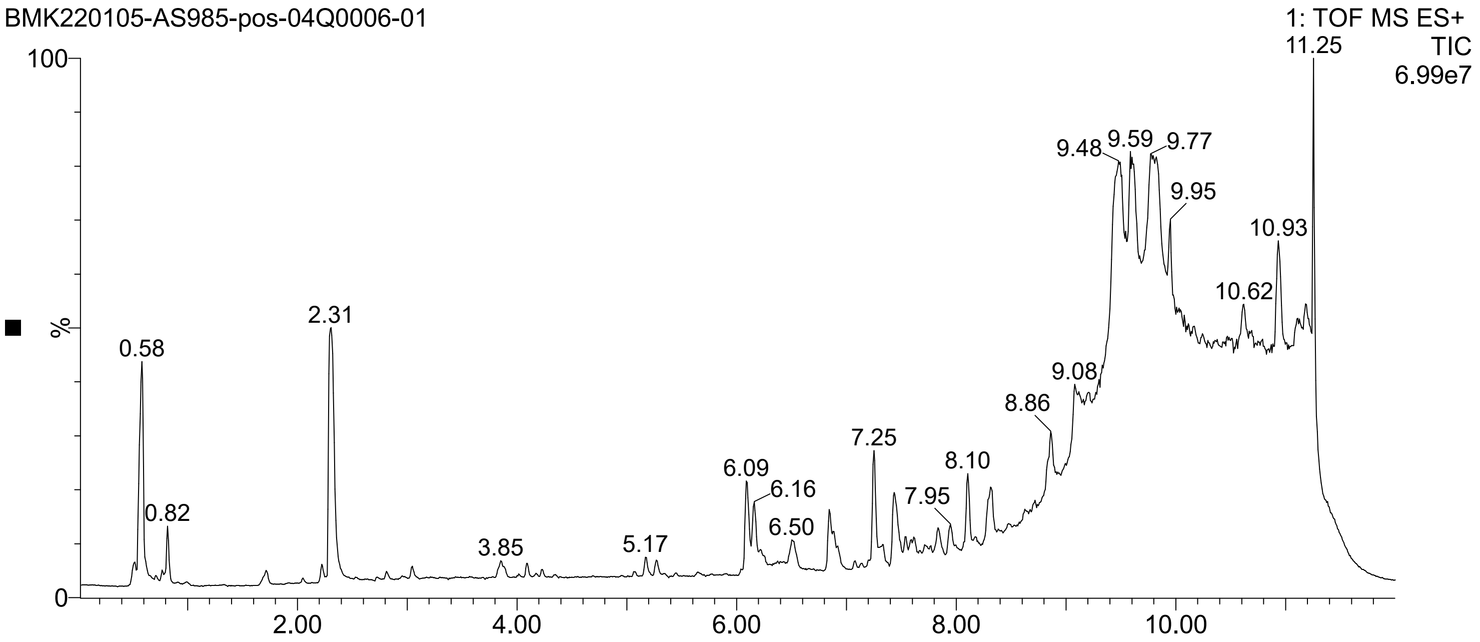
BMK220105-AS985-pos-04Q0006-01
1: TOF MS ES+
TIC
6.99e7
11.25
100
%
0
9.48
9.59
9.77
9.95
10.93
10.62
2.31
0.58
9.08
8.86
7.25
8.10
6.09
6.16
7.95
0.82
6.50
3.85
5.17
2.00
4.00
6.00
8.00
10.00

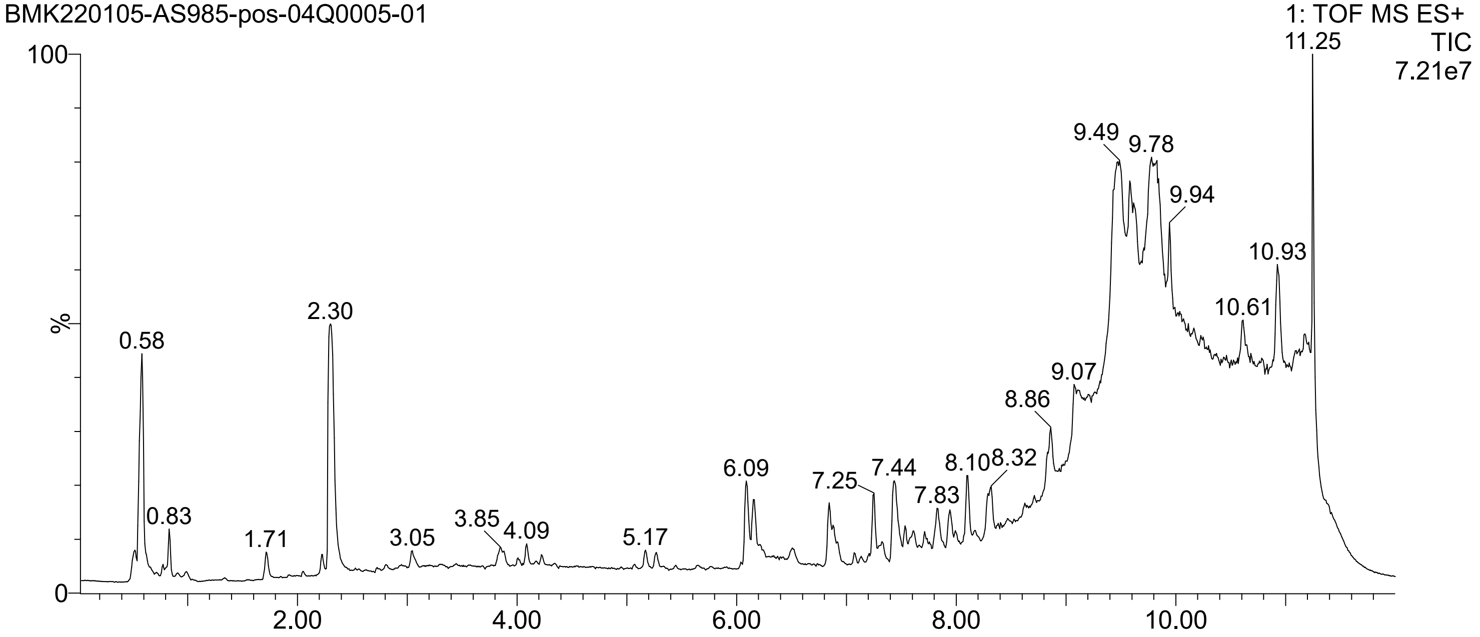
BMK220105-AS985-pos-04Q0005-01
1: TOF MS ES+
TIC
7.21e7
11.25
100
%
0
9.49
9.78
9.94
10.93
10.61
2.30
0.58
9.07
8.86
6.09
7.25
7.44
8.10
8.32
7.83
0.83
1.71
3.05
3.85
4.09
5.17
2.00
4.00
6.00
8.00
10.00

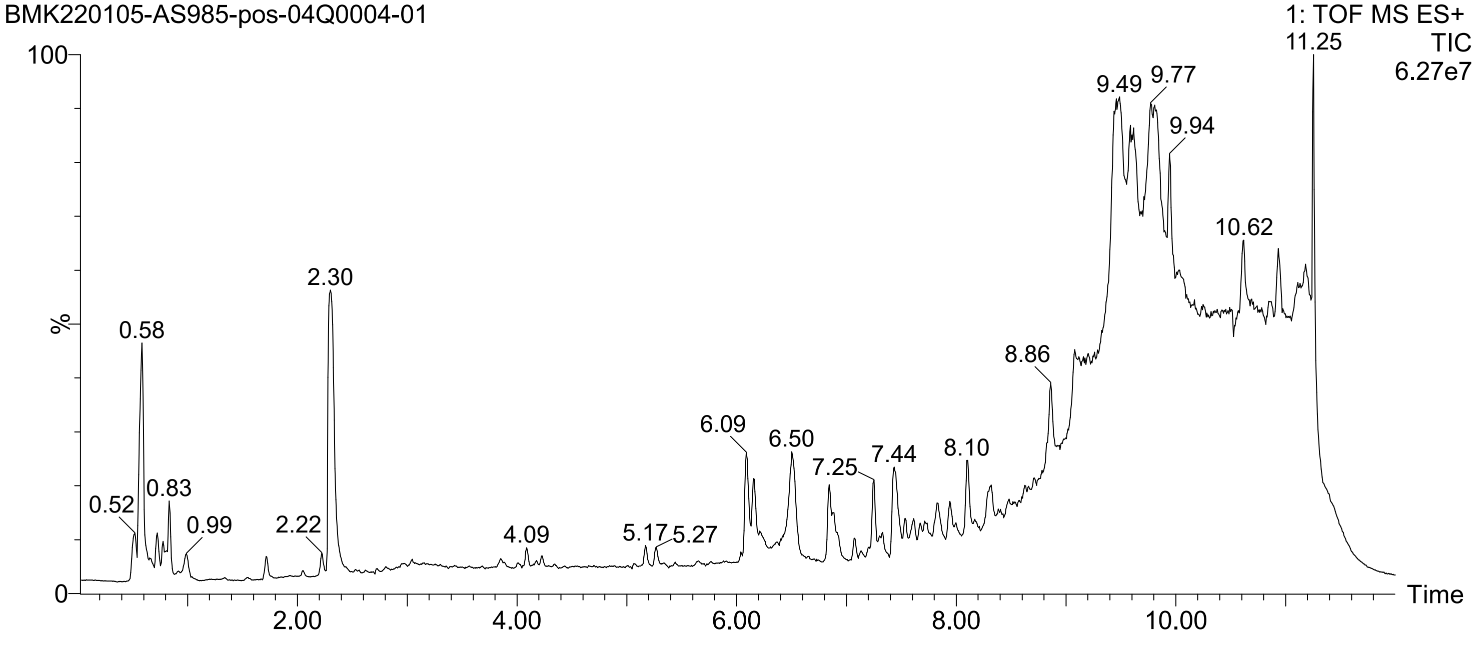
BMK220105-AS985-pos-04Q0004-01
1: TOF MS ES+
TIC
6.27e7
11.25
100
%
0
9.49
9.77
9.94
10.62
2.30
0.58
8.86
6.09
6.50
7.25
7.44
8.10
0.83
0.52
0.99
2.22
4.09
5.17
5.27
2.00
4.00
6.00
8.00
10.00
Time

BMK220105-AS985-pos-04Q0009-01

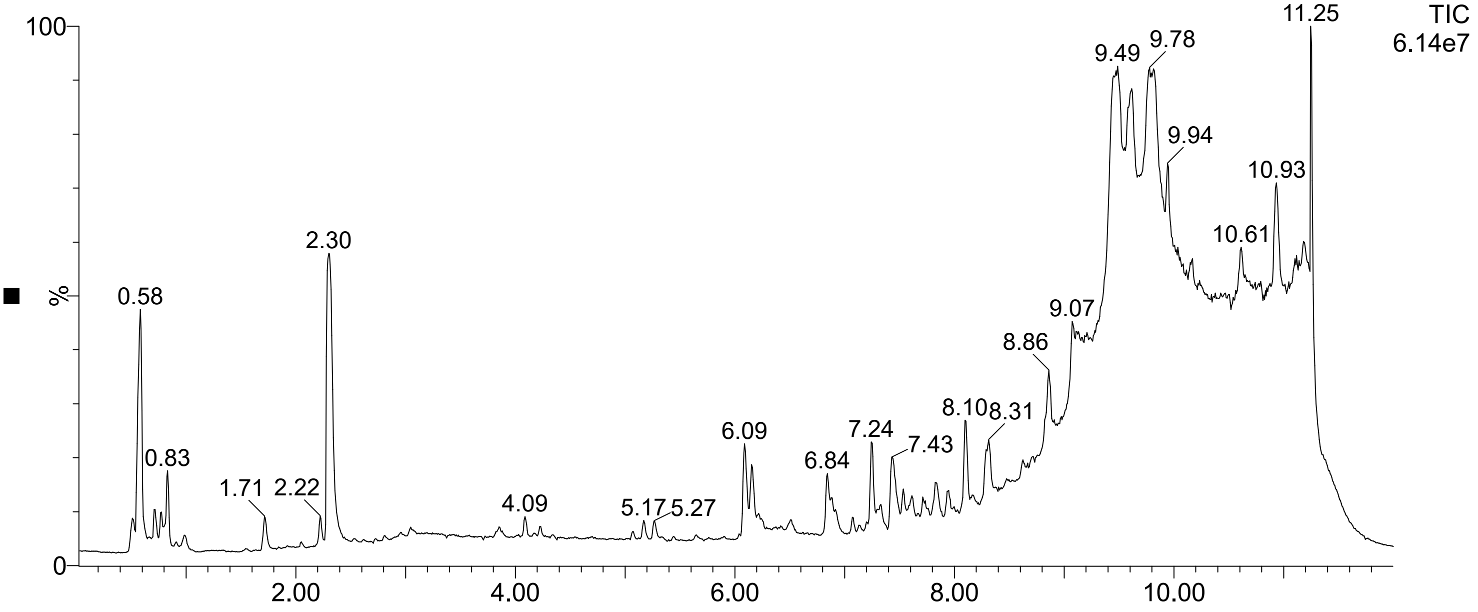

BMK220105-AS985-pos-04Q0008-01

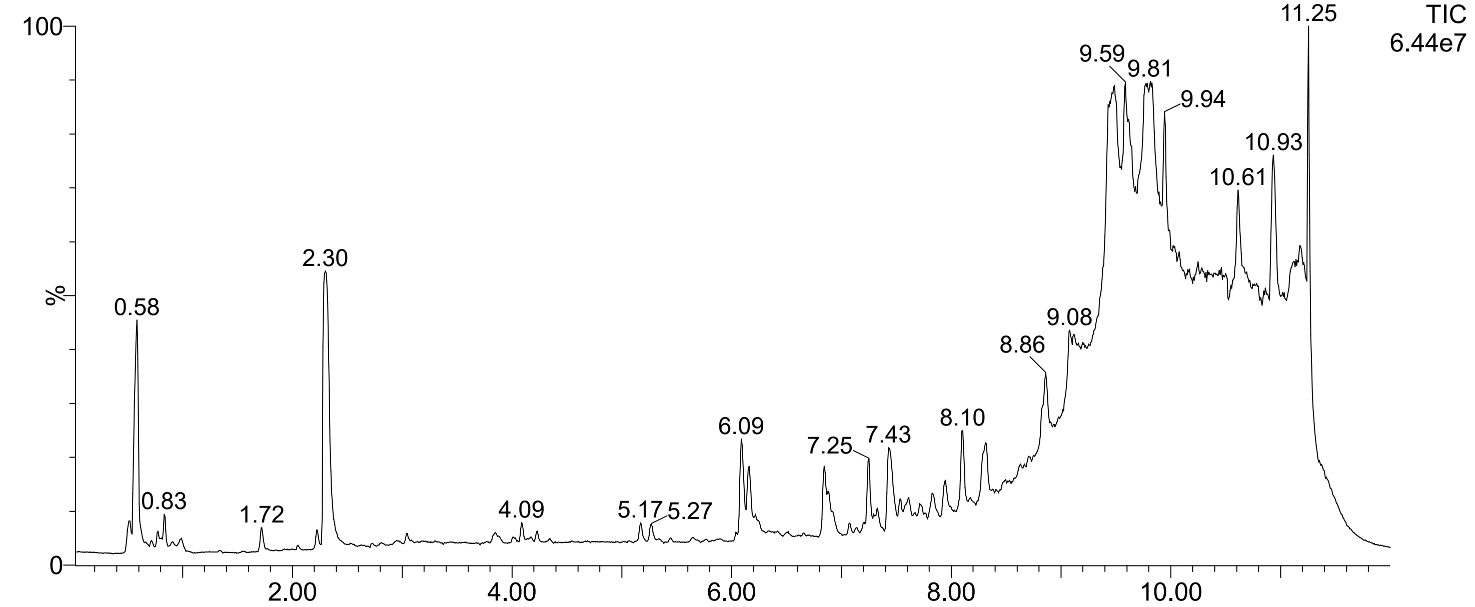

BMK220105-AS985-pos-04Q0007-01

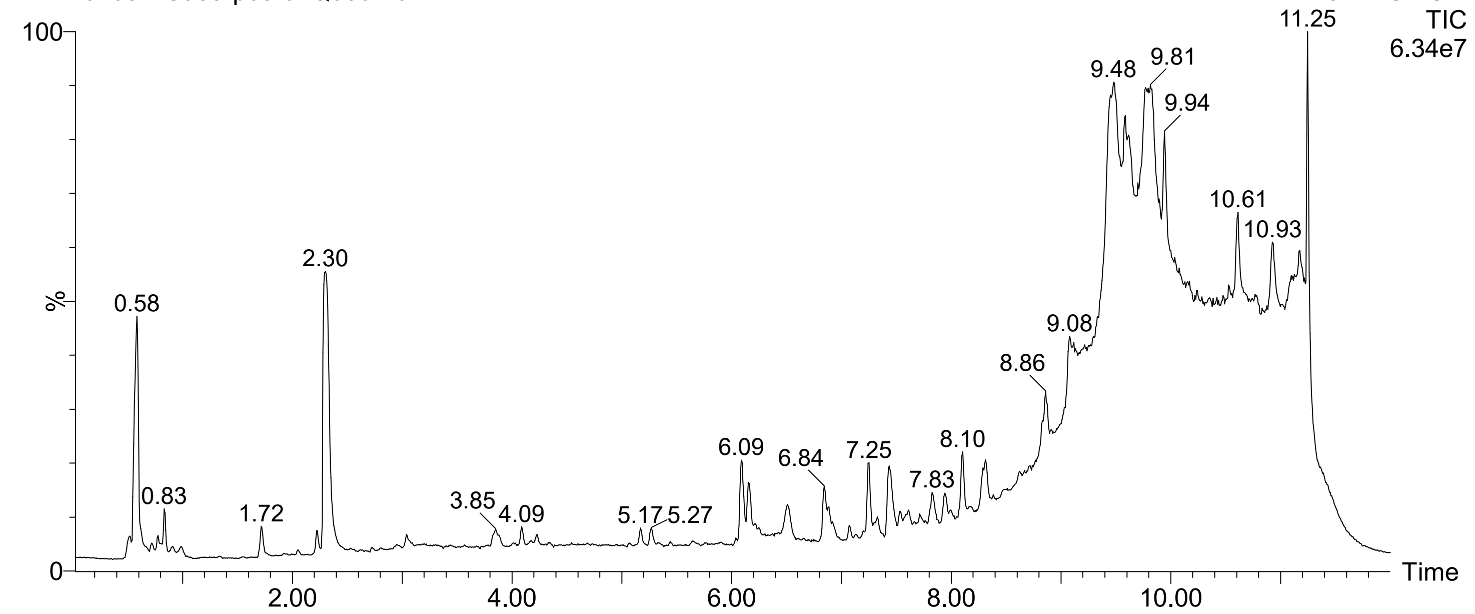

BMK220105-AS985-pos-04Q0012-01

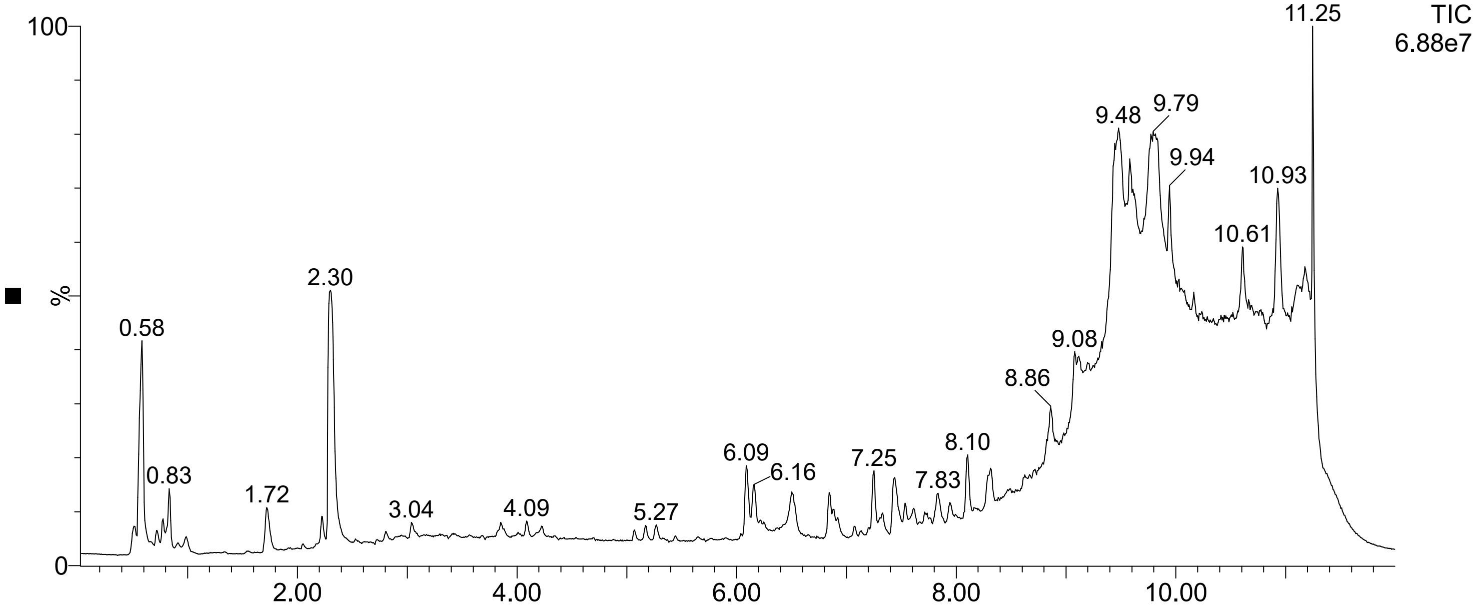

BMK220105-AS985-pos-04Q0011-01

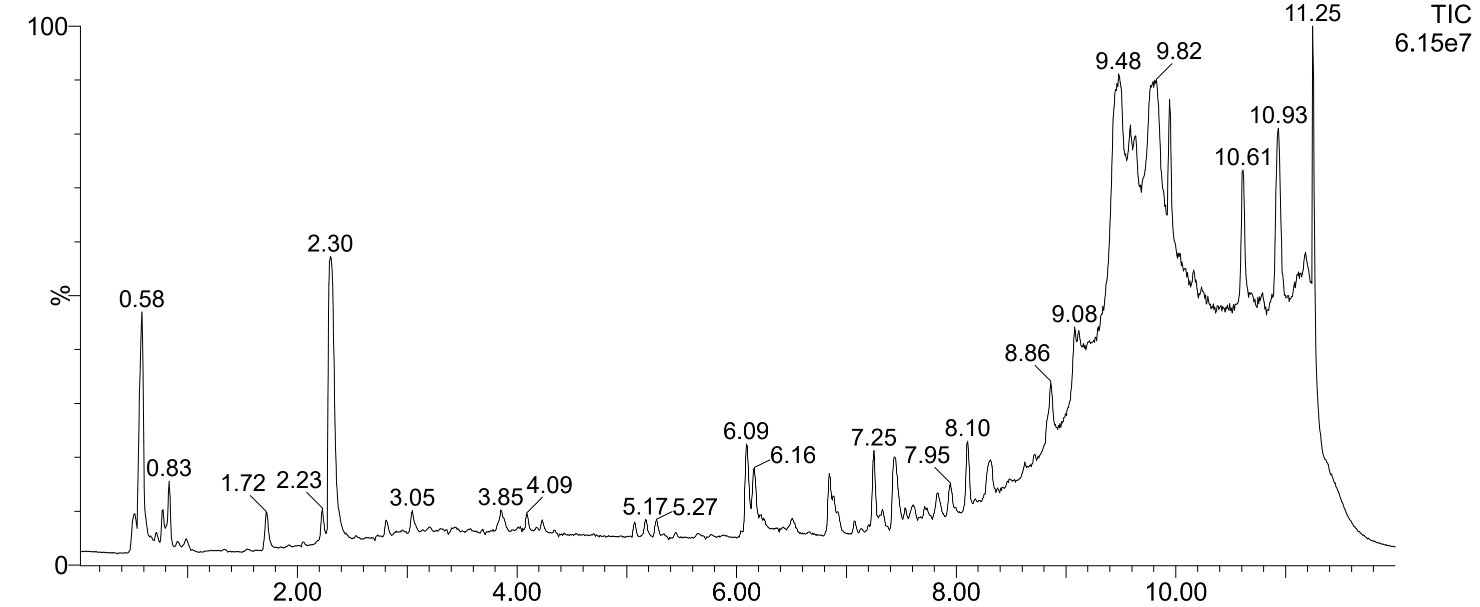

BMK220105-AS985-pos-04Q0010-01

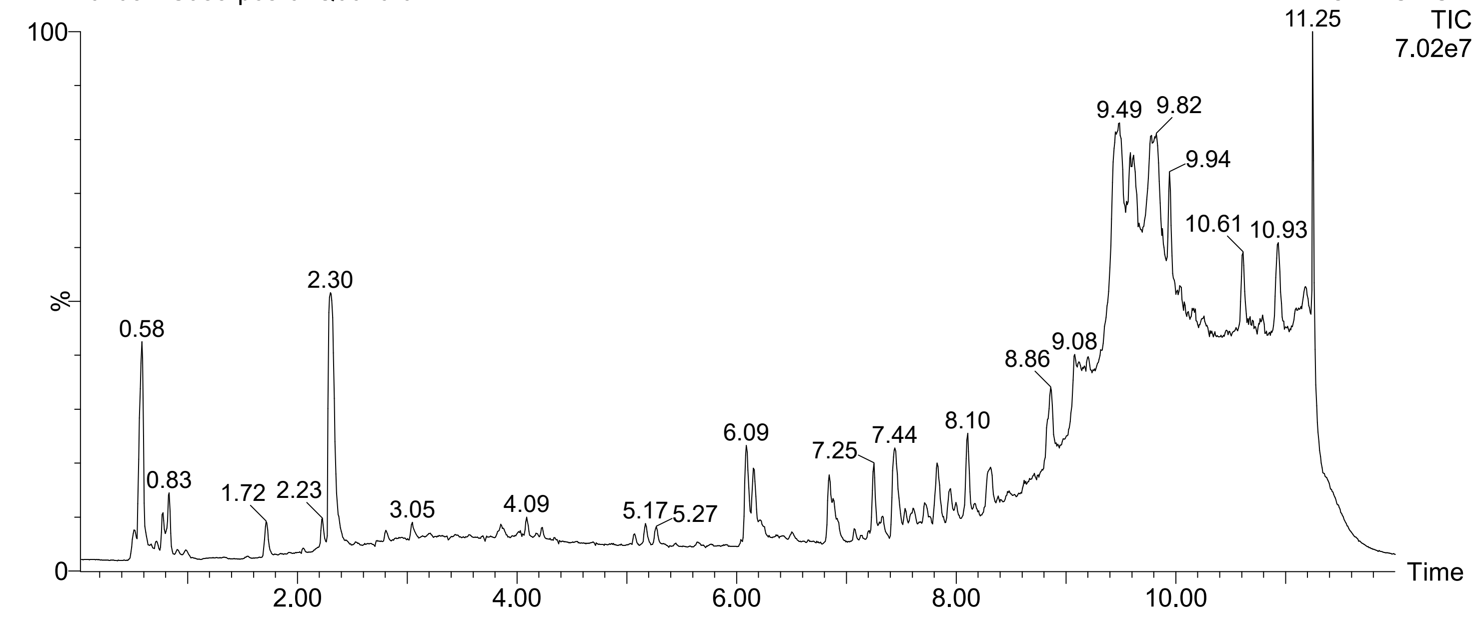

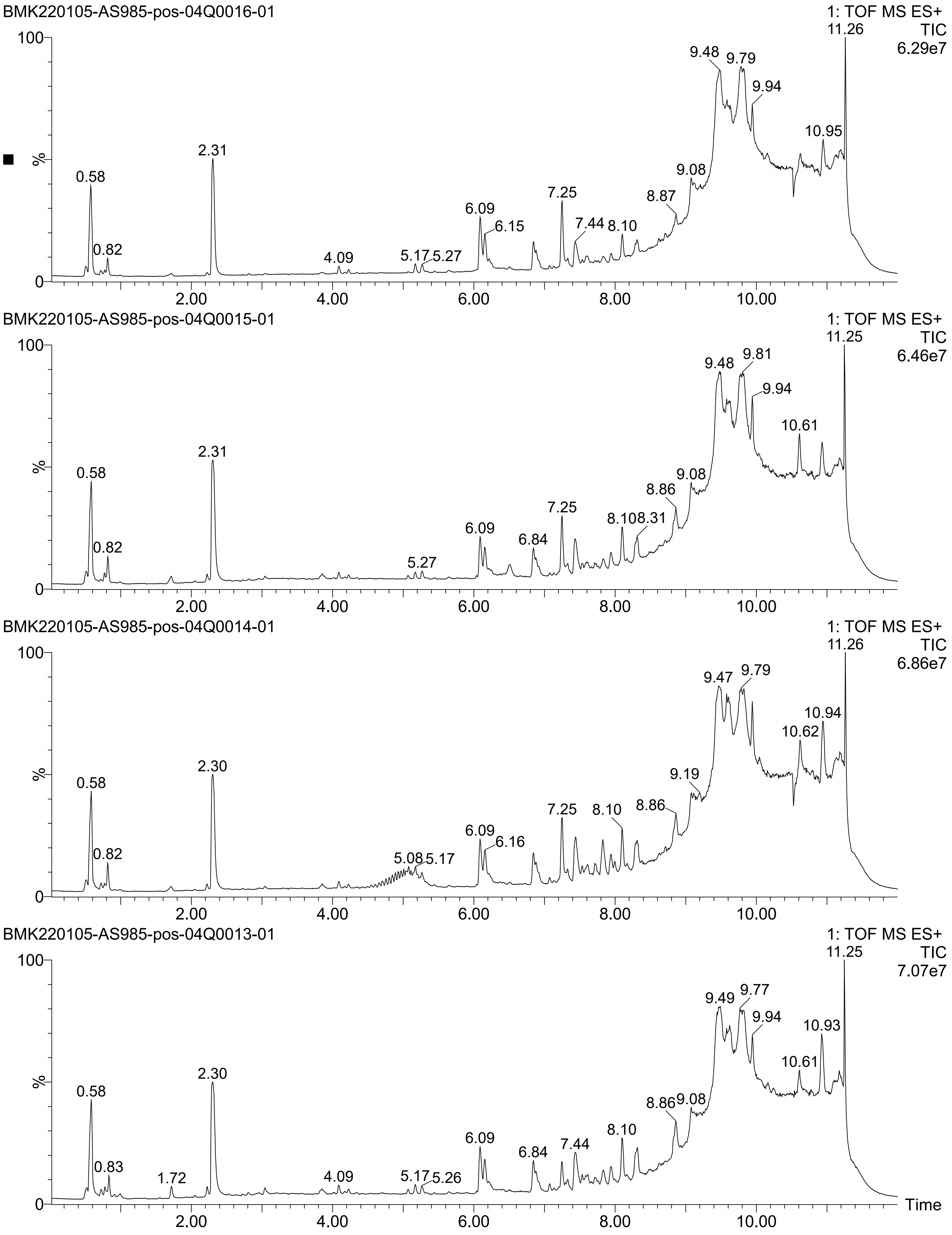

BMK220105-AS985-pos-04Q0016-01
1: TOF MS ES+
TIC
6.29e7
11.26
9.48
9.79
9.94
10.95
9.08
8.87
7.25
7.44
8.10
6.09
6.15
5.17
5.27
4.09
2.31
0.58
0.82
BMK220105-AS985-pos-04Q0015-01
1: TOF MS ES+
TIC
6.46e7
11.25
9.48
9.81
9.94
10.61
9.08
8.86
8.10
8.31
7.25
6.84
6.09
5.27
2.31
0.58
0.82
BMK220105-AS985-pos-04Q0014-01
1: TOF MS ES+
TIC
6.86e7
11.26
9.47
9.79
10.94
10.62
9.19
8.86
8.10
7.25
6.09
6.16
5.08
5.17
2.30
0.58
0.82
BMK220105-AS985-pos-04Q0013-01
1: TOF MS ES+
TIC
7.07e7
11.25
9.49
9.77
9.94
10.93
10.61
8.86
9.08
8.10
7.44
6.84
6.09
5.17
5.26
4.09
2.30
1.72
0.58
0.83
%
100
0
2.00
4.00
6.00
8.00
10.00
Time

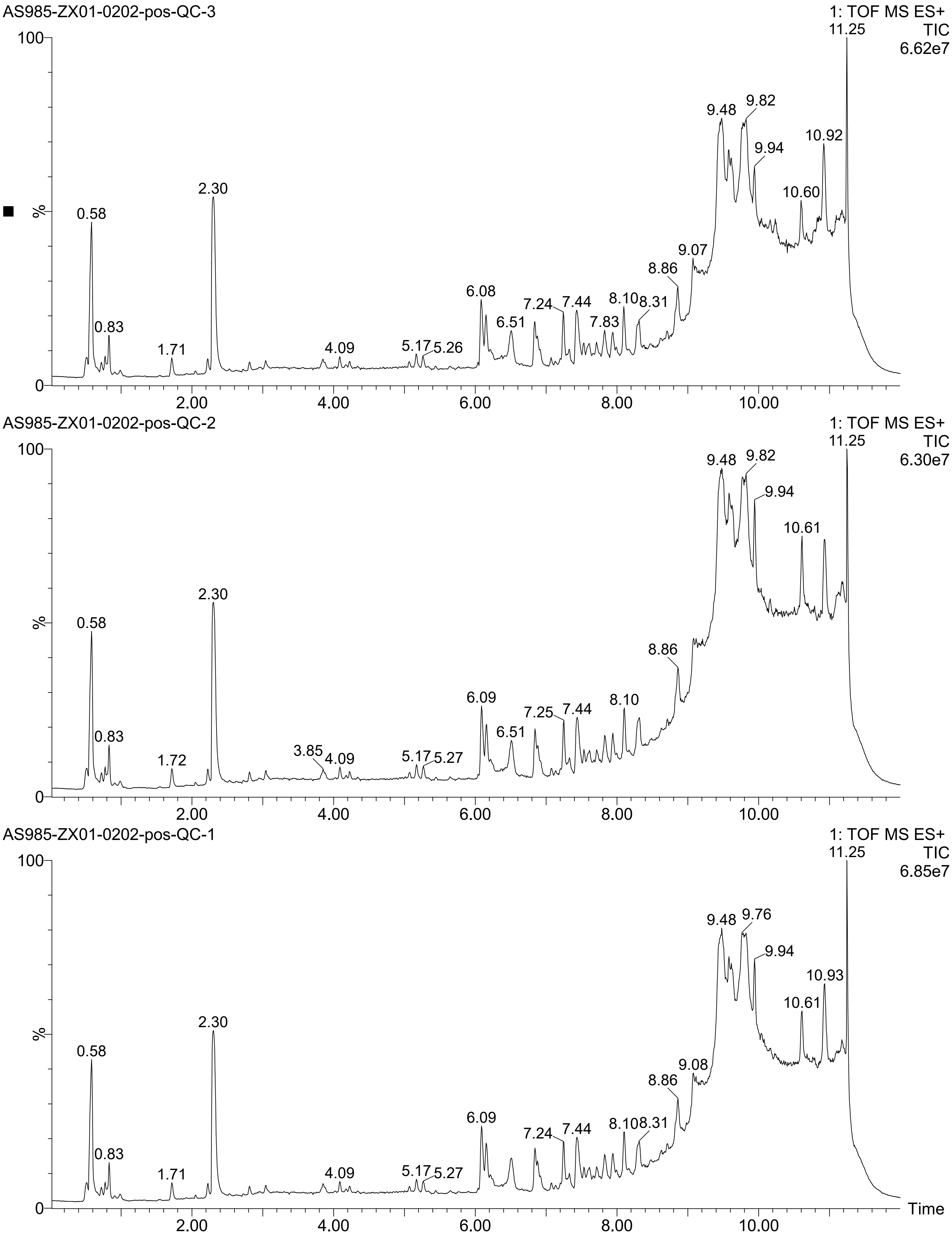
AS985-ZX01-0202-pos-QC-3
1: TOF MS ES+
TIC
6.62e7
11.25
9.48
9.82
9.94
10.92
10.60
2.30
0.58
9.07
8.86
6.08
7.24
7.44
8.10
8.31
6.51
7.83
0.83
1.71
4.09
5.17
5.26
%
100
0
2.00
4.00
6.00
8.00
10.00
AS985-ZX01-0202-pos-QC-2
1: TOF MS ES+
TIC
6.30e7
11.25
9.48
9.82
9.94
10.61
2.30
0.58
8.86
6.09
7.25
7.44
8.10
6.51
0.83
1.72
3.85
4.09
5.17
5.27
%
100
0
2.00
4.00
6.00
8.00
10.00
AS985-ZX01-0202-pos-QC-1
1: TOF MS ES+
TIC
6.85e7
11.25
9.48
9.76
9.94
10.93
10.61
2.30
0.58
9.08
8.86
6.09
7.24
7.44
8.10
8.31
0.83
1.71
4.09
5.17
5.27
%
100
0
2.00
4.00
6.00
8.00
10.00
Time
