# Supplementary material for: Quiescence preconditioned nucleus pulposus stem cells alleviate intervertebral disc degeneration by enhancing cell survival via adaptive metabolism pattern in rats
Source: Front Bioeng Biotechnol. 2023 Feb 10;11:1073238. doi: 10.3389/fbioe.2023.1073238 (PMC9950514; doi:10.3389/fbioe.2023.1073238)
Supplement: Supplementary file 2 [file DataSheet1.zip › Supplementary Material.pdf]

Supplementary Material

1 Supplementary Data

Supplementary File 1: Metabolomic detection was performed between the P-NPSCs and Q-NPSCs groups. A total of 3589 metabolites were annotated using the online METLIN database, HMDB, and the self-built Biomarker Co. database with 483 downregulated and 257 upregulated metabolites. P-NPSCs: proliferating NPSCs; Q-NPSCs: quiescent NPSCs.

Supplementary File 2: Expression levels of all metabolites in P-NPSCs and Q-NPSCs.

Supplementary File 3: The expression levels of differential metabolites in P-NPSCs and Q-NPSCs were represented by volcano and heatmap.

Supplementary File 4: The expression of top 30 metabolites in the P-NPSC and Q-NPSC groups.

Supplementary File 5: The pathway enrichment analysis was performed using the KEGG database for these differential metabolites in the P-NPSCs and Q-NPSCs groups.

Supplementary File 6: The number of up- and down-regulated differential metabolites in the differential metabolic pathway between the P-NPSCs and Q-NPSCs groups.

2 Supplementary Figures

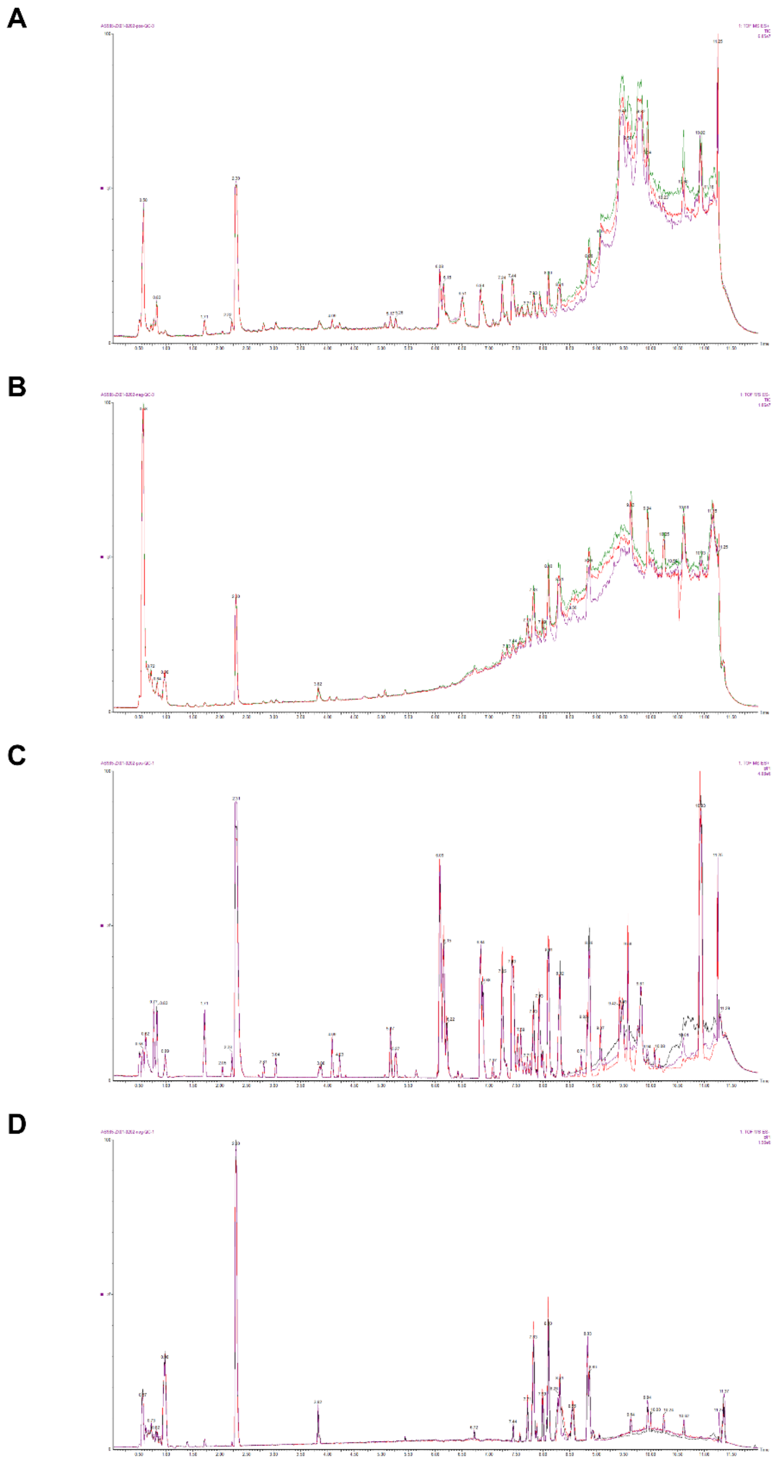

Supplementary Figure 1. Total ion chromatogram (TIC) in positive **(A)** and negative **(B)** ion modes. Base Peak Chromatogram (BPC) in positive **(C)** and negative **(D)** ion modes. Based on the LC-QTOF platform, qualitative and quantitative analyses were performed for metabolomics on the

eight samples of P-NPSCs and Q-NPSCs, and a total of 14,809 peaks were detected. P-NPSCs: proliferating NPSCs; Q-NPSCs: quiescent NPSCs.
