# Supplementary figures and images for: Quiescence preconditioned nucleus pulposus stem cells alleviate intervertebral disc degeneration by enhancing cell survival via adaptive metabolism pattern in rats
Source: Front Bioeng Biotechnol. 2023 Feb 10;11:1073238. doi: 10.3389/fbioe.2023.1073238 (PMC9950514; doi:10.3389/fbioe.2023.1073238)

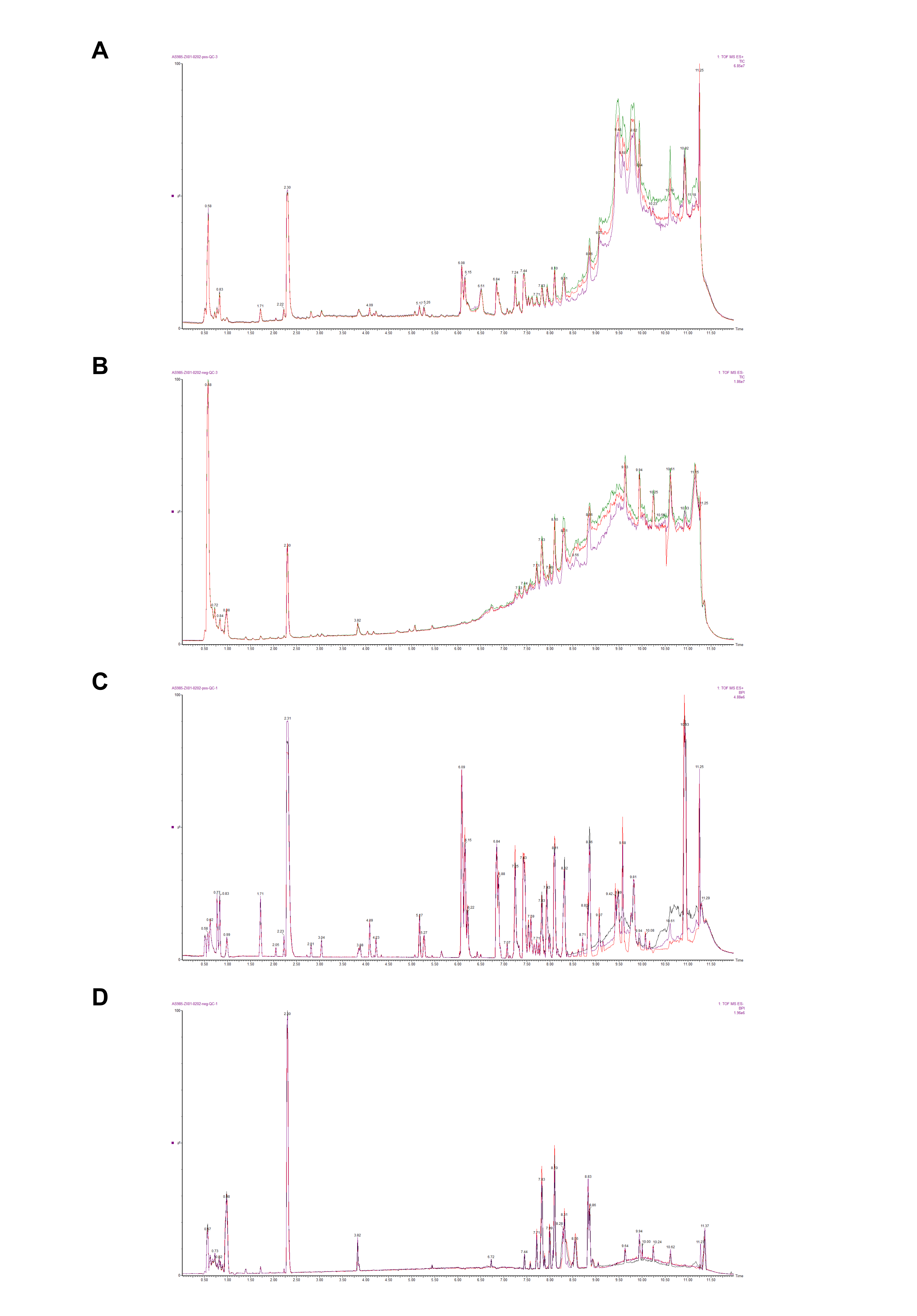

Supplement: Supplementary file 2 [file DataSheet1.zip › Supplementary Figure S1.tif]

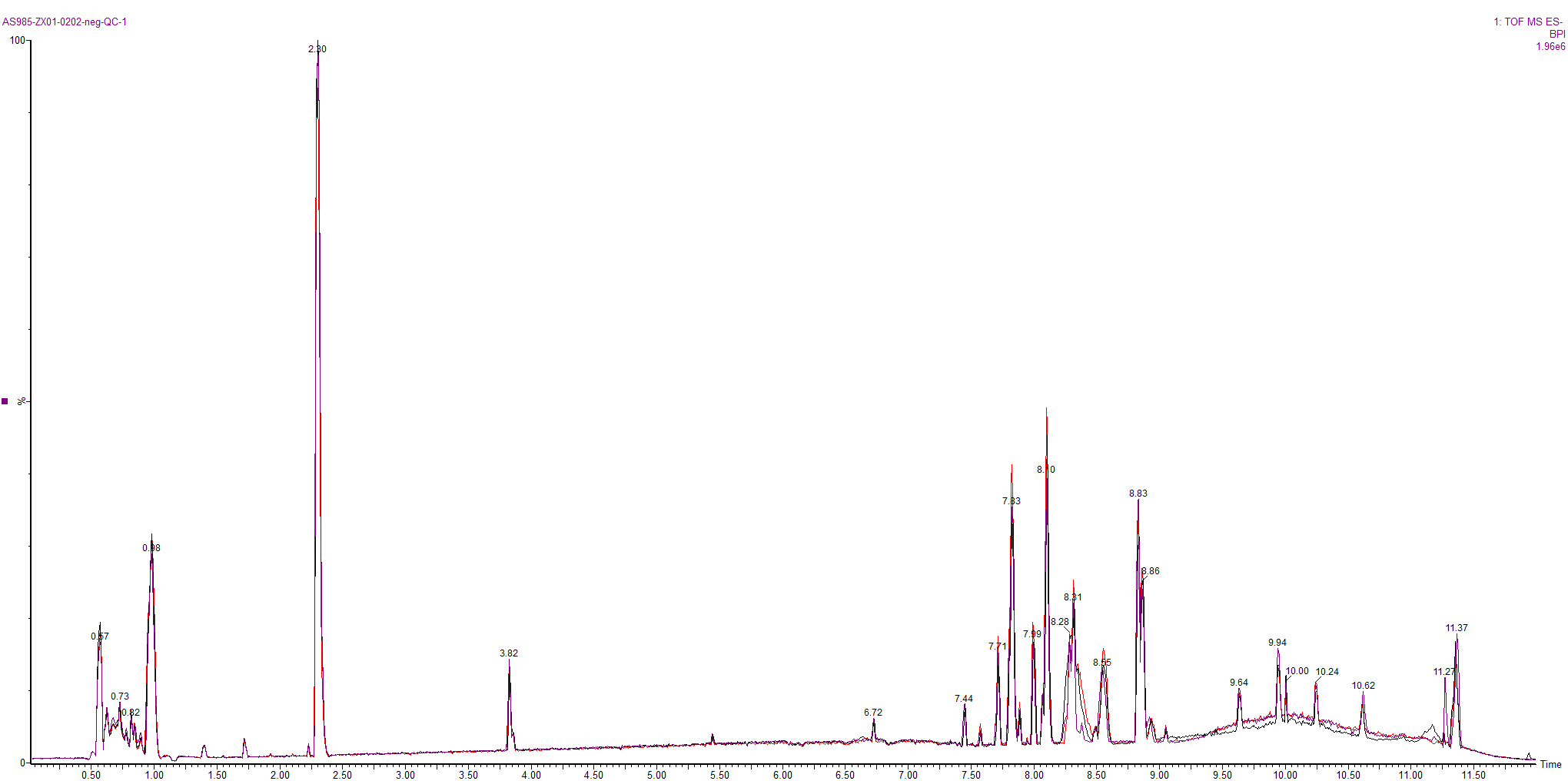

Supplement: Supplementary file 2 [file DataSheet1.zip › Supplementary Figure S1_raw data/bpi-nb/neg-bpi.bmp]

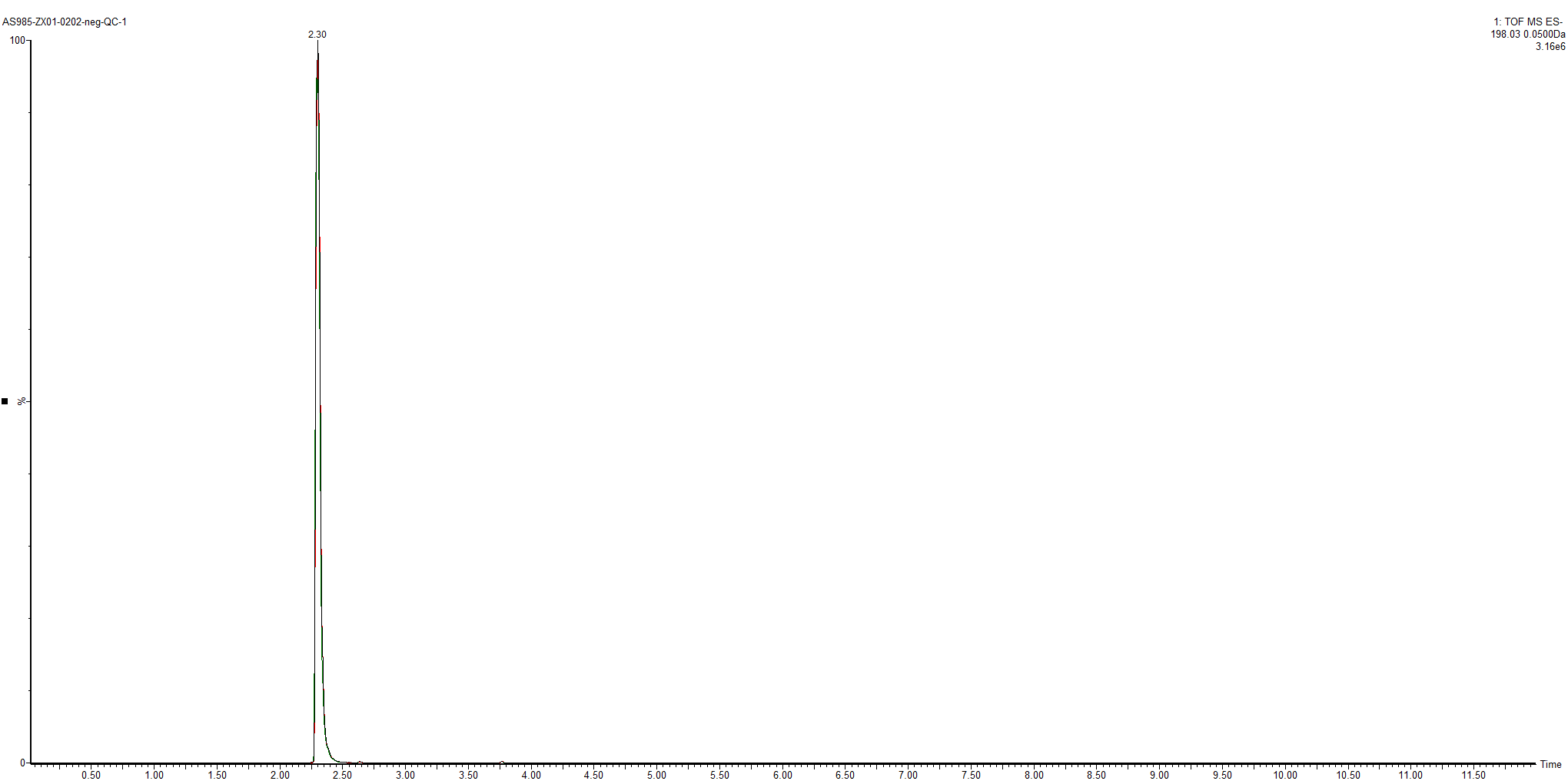

Supplement: Supplementary file 2 [file DataSheet1.zip › Supplementary Figure S1_raw data/bpi-nb/neg-nb.bmp]

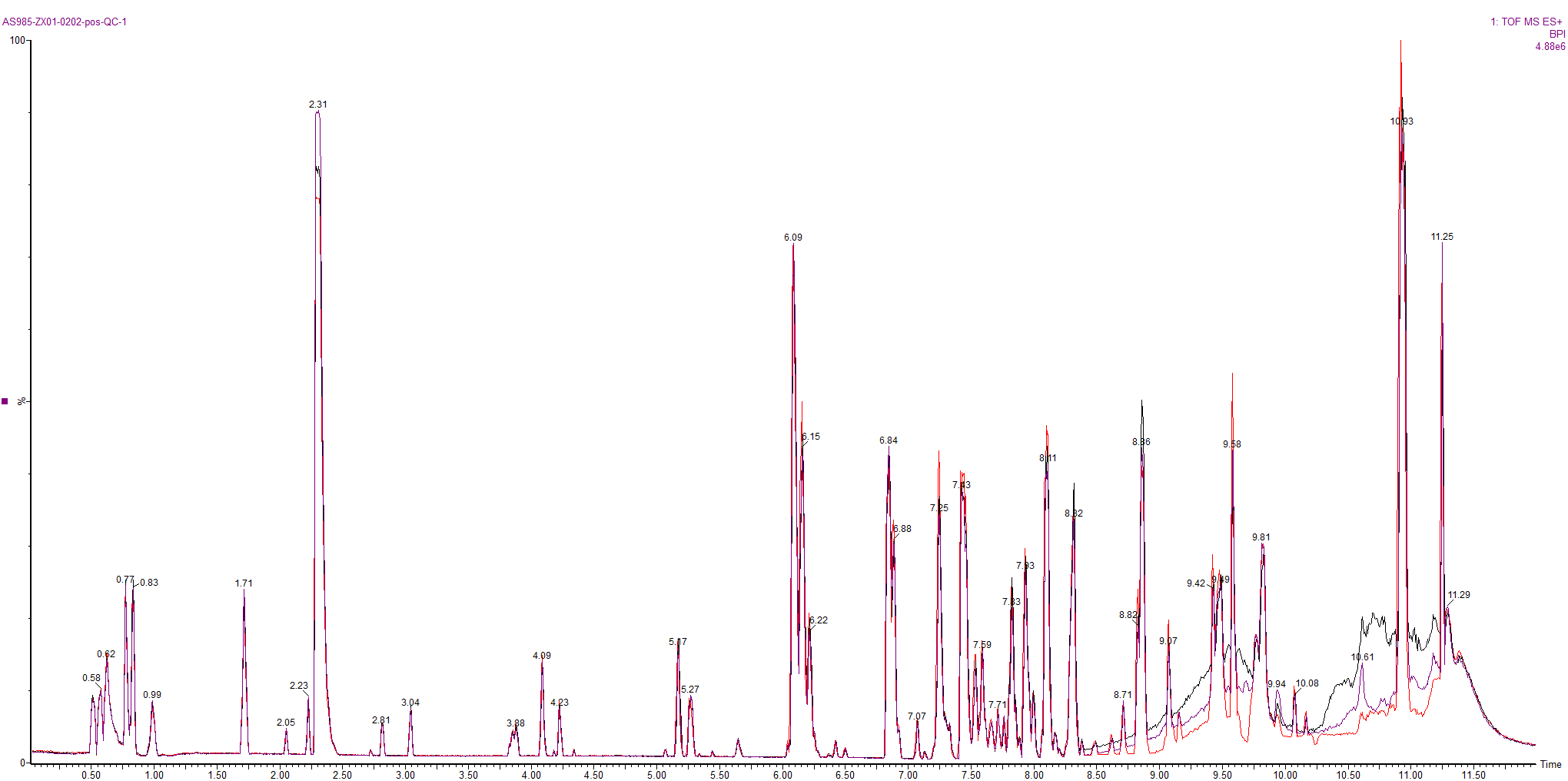

Supplement: Supplementary file 2 [file DataSheet1.zip › Supplementary Figure S1_raw data/bpi-nb/pos-bpi.bmp]

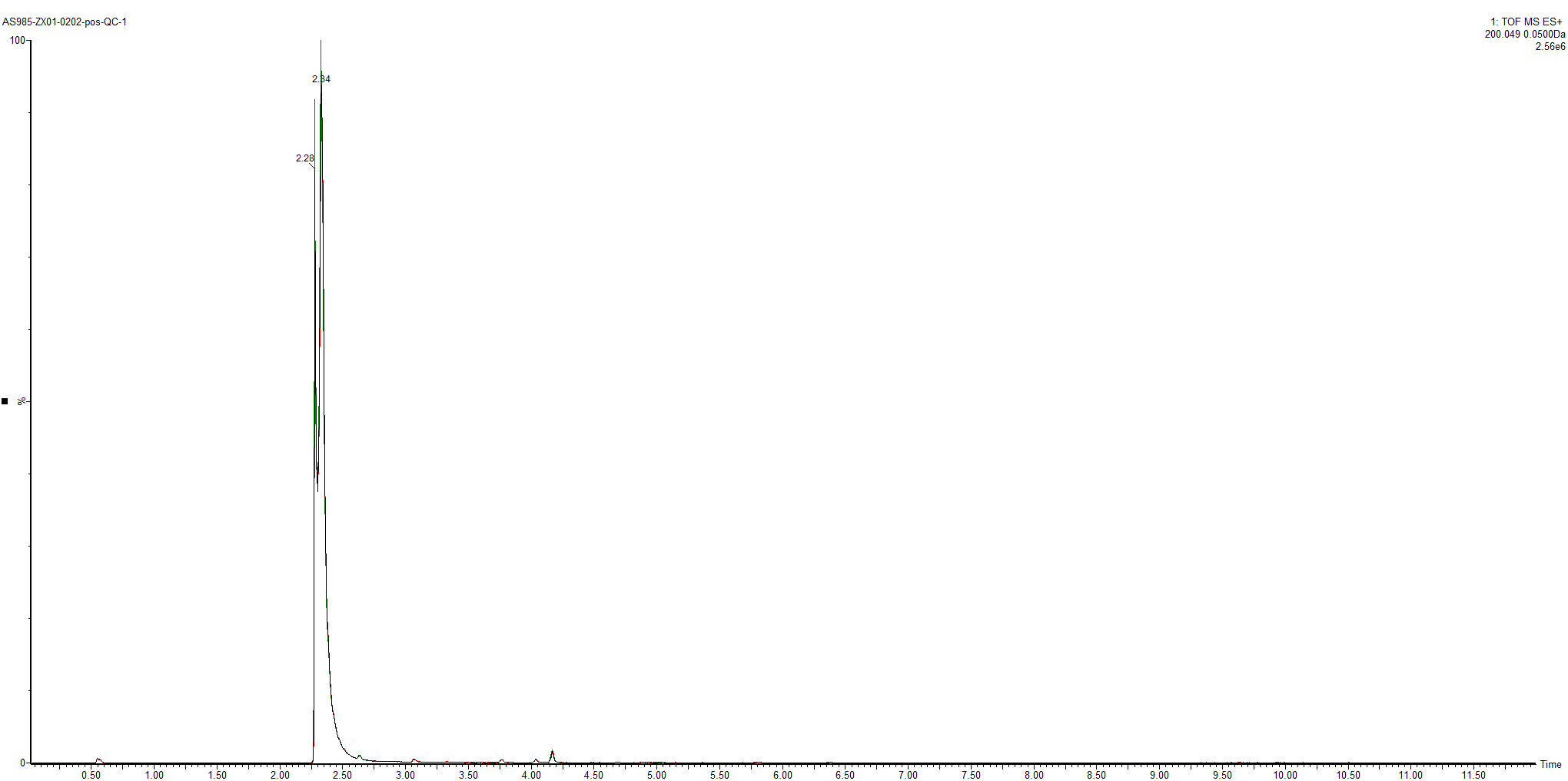

Supplement: Supplementary file 2 [file DataSheet1.zip › Supplementary Figure S1_raw data/bpi-nb/pos-nb.bmp]

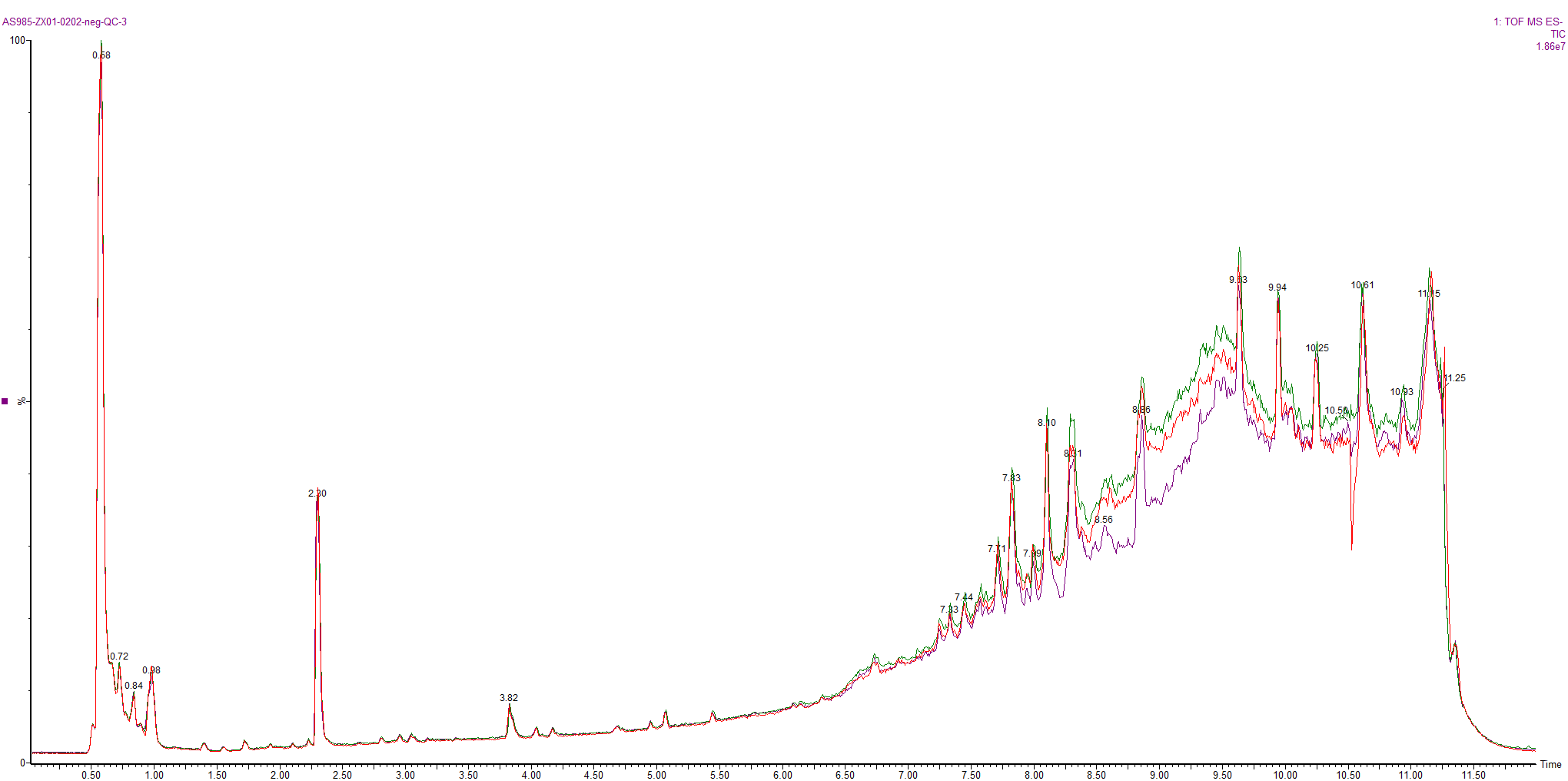

Supplement: Supplementary file 2 [file DataSheet1.zip › Supplementary Figure S1_raw data/tic/neg-tic.bmp]

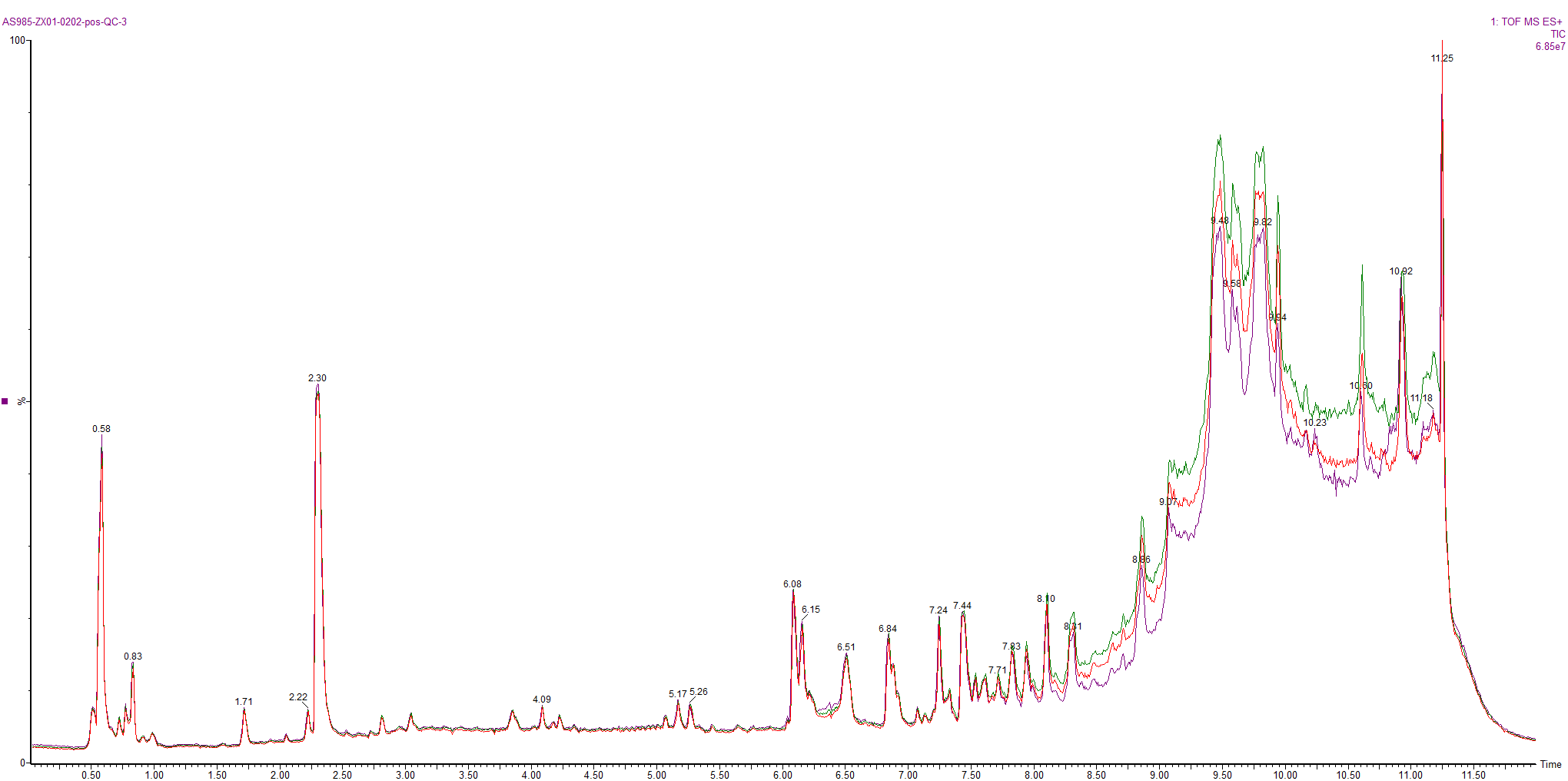

Supplement: Supplementary file 2 [file DataSheet1.zip › Supplementary Figure S1_raw data/tic/pos-tic.bmp]

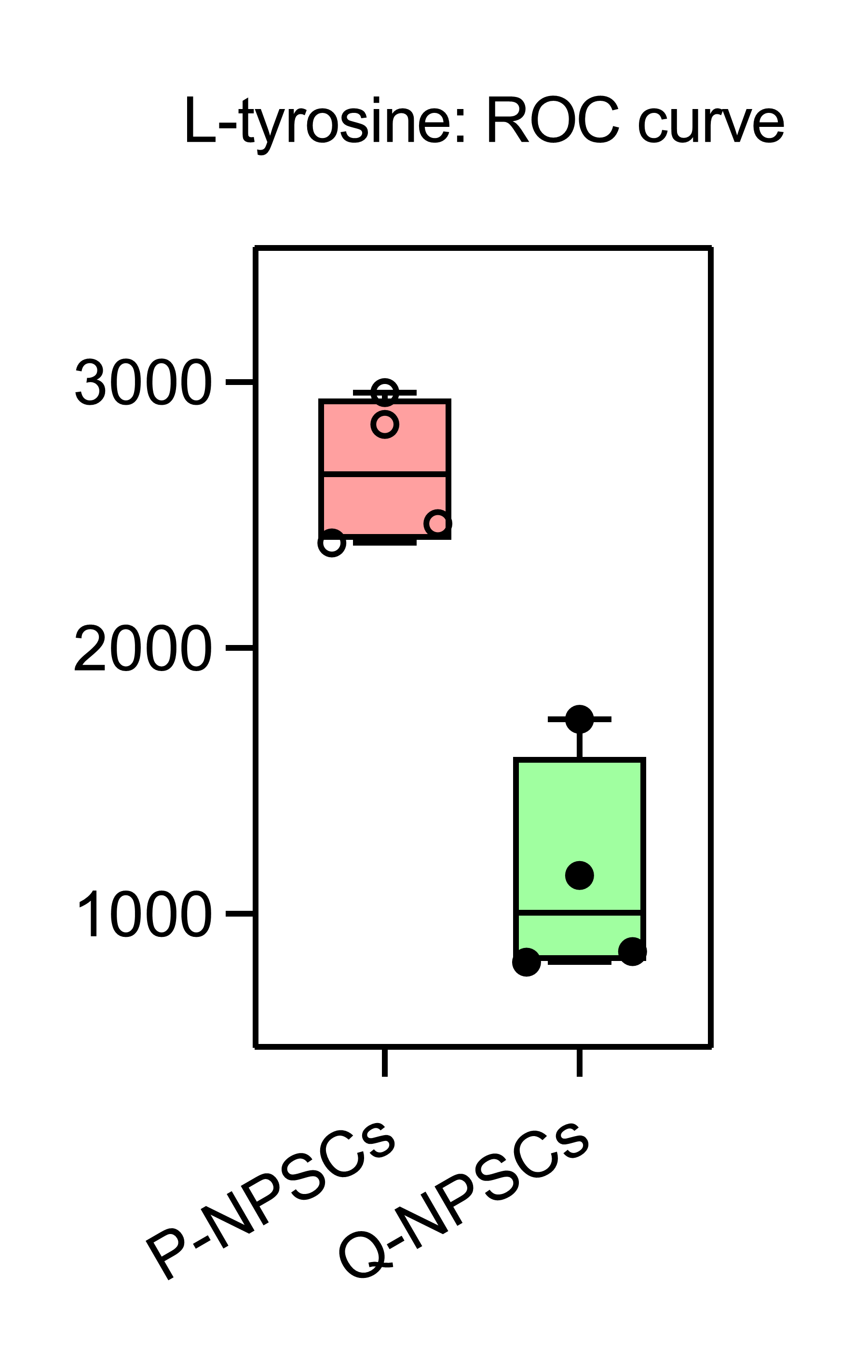

Supplement: Supplementary file 2 [file DataSheet1.zip › Supplementary File_ROC/neg_1122_L-tyrosine ROC curve (1).tif]

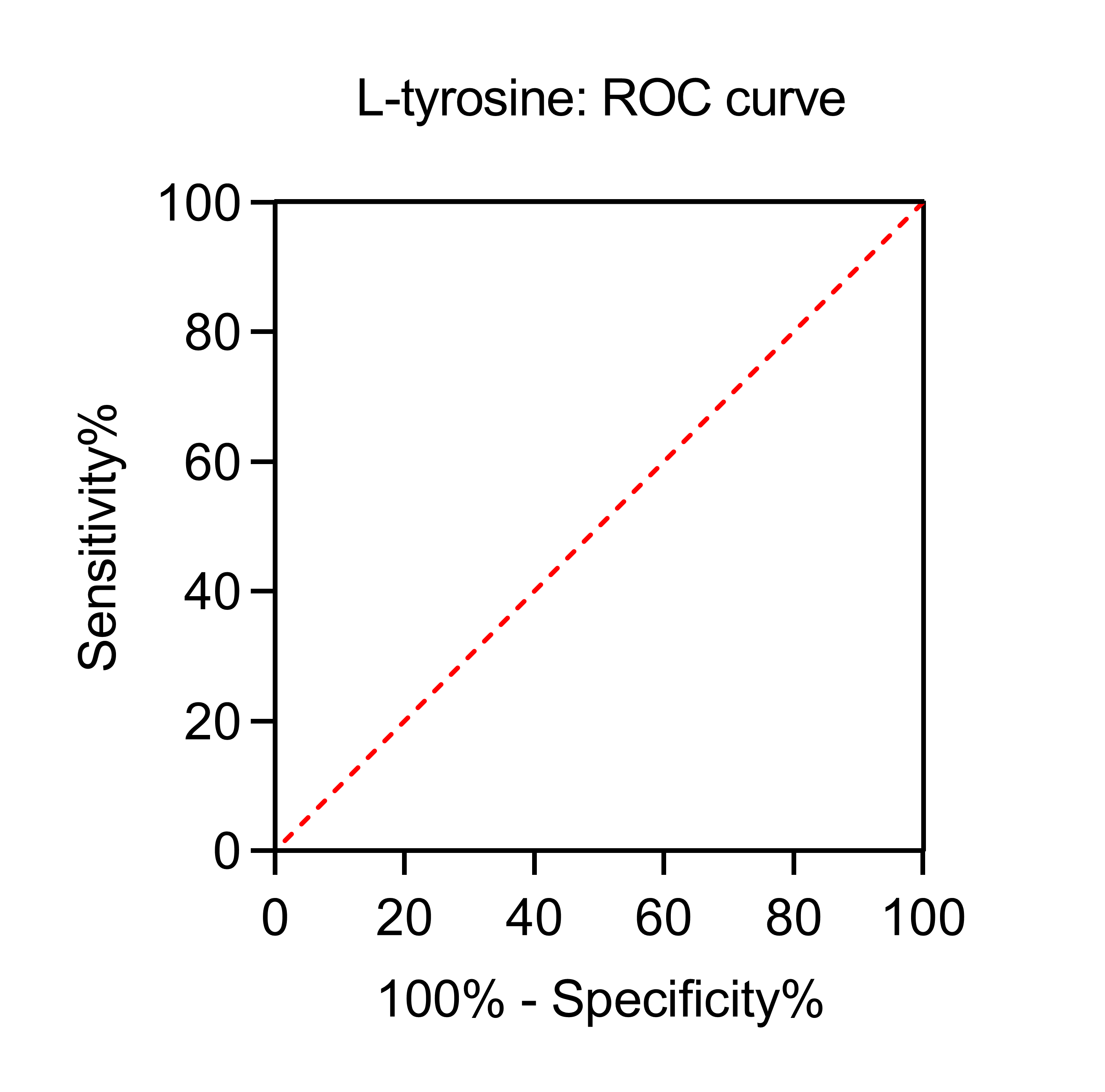

Supplement: Supplementary file 2 [file DataSheet1.zip › Supplementary File_ROC/neg_1122_L-tyrosine ROC curve (2).tif]

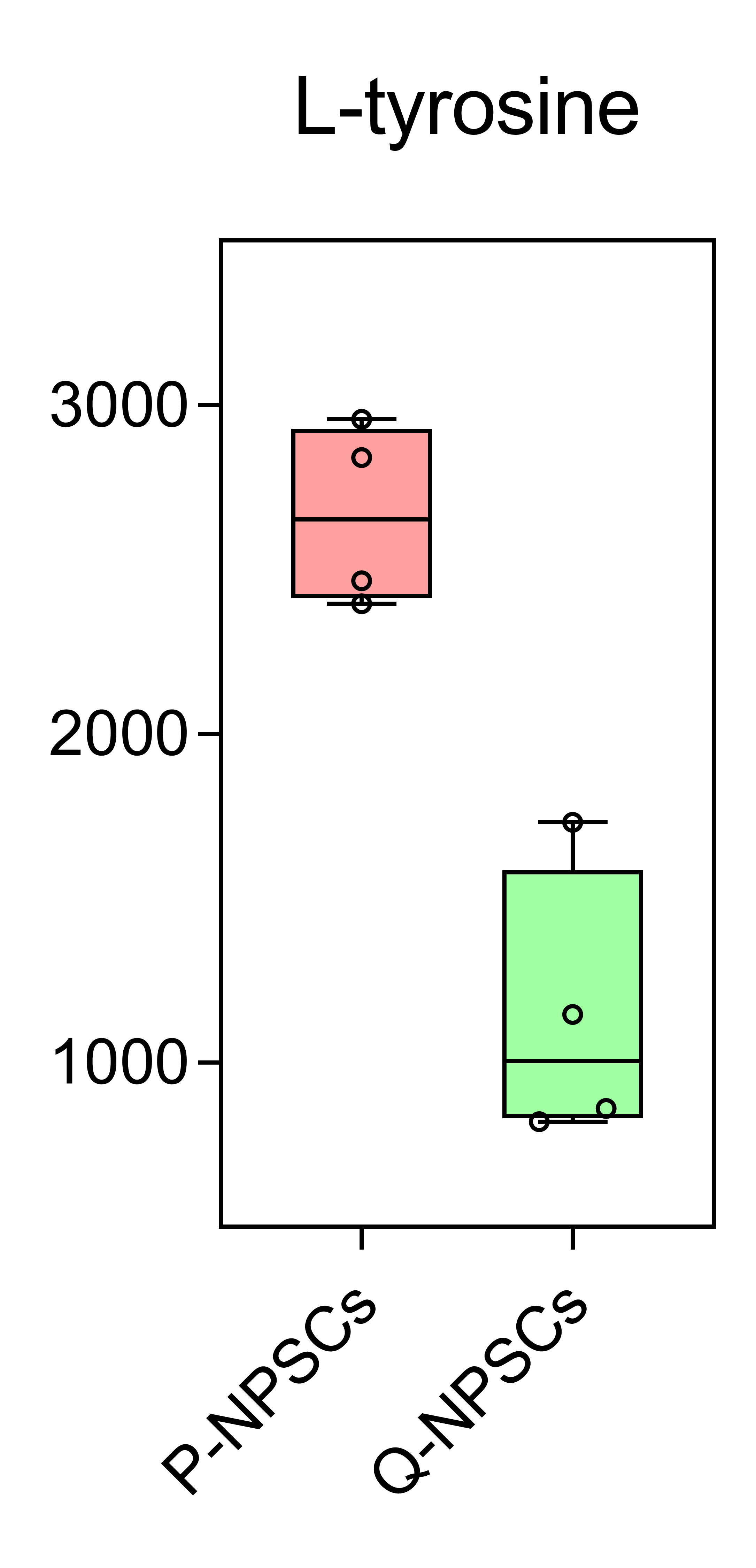

Supplement: Supplementary file 2 [file DataSheet1.zip › Supplementary File_ROC/neg_1122_L-tyrosine.tif]

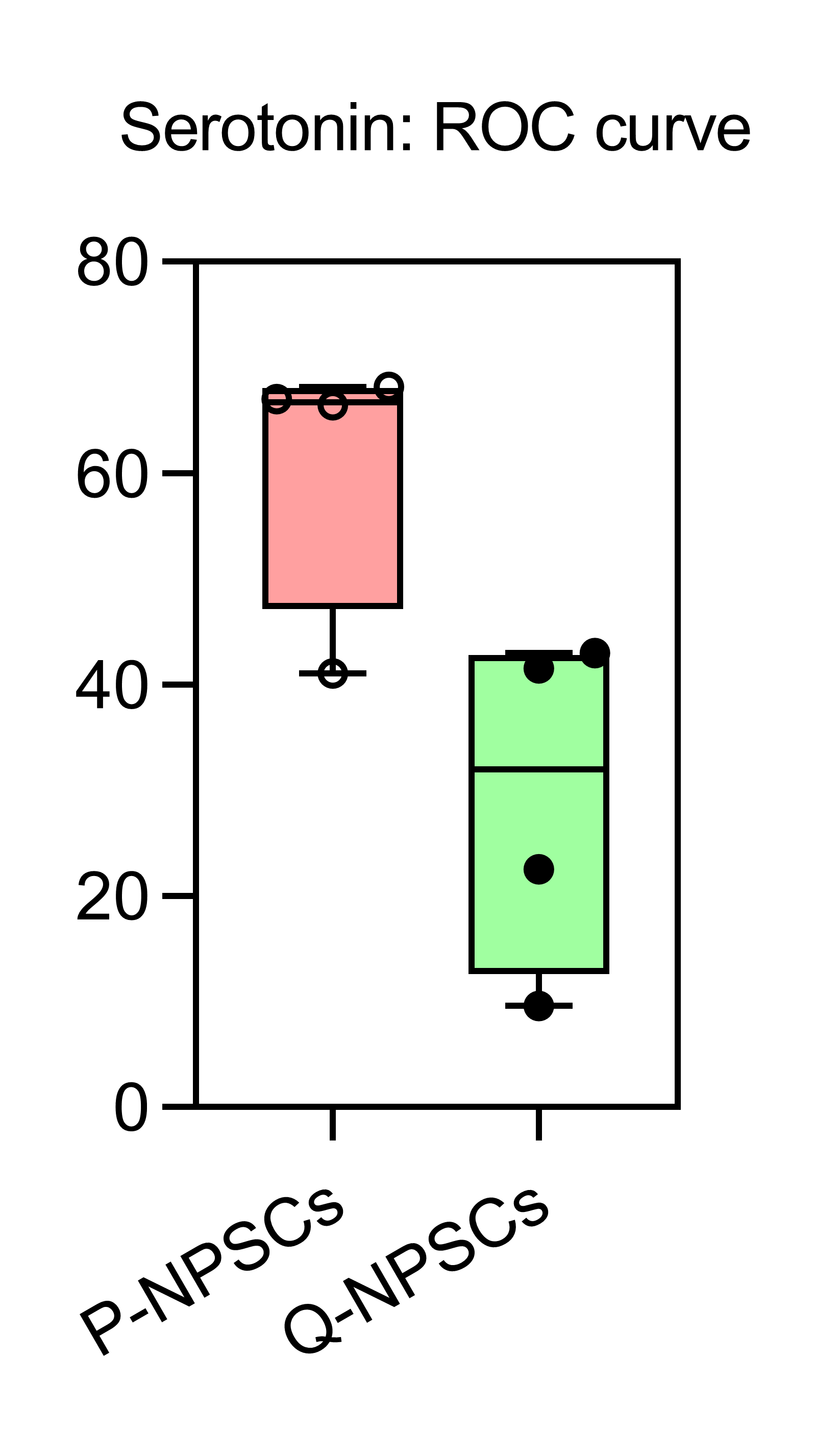

Supplement: Supplementary file 2 [file DataSheet1.zip › Supplementary File_ROC/neg_1997_Serotonin ROC curve (1).tif]

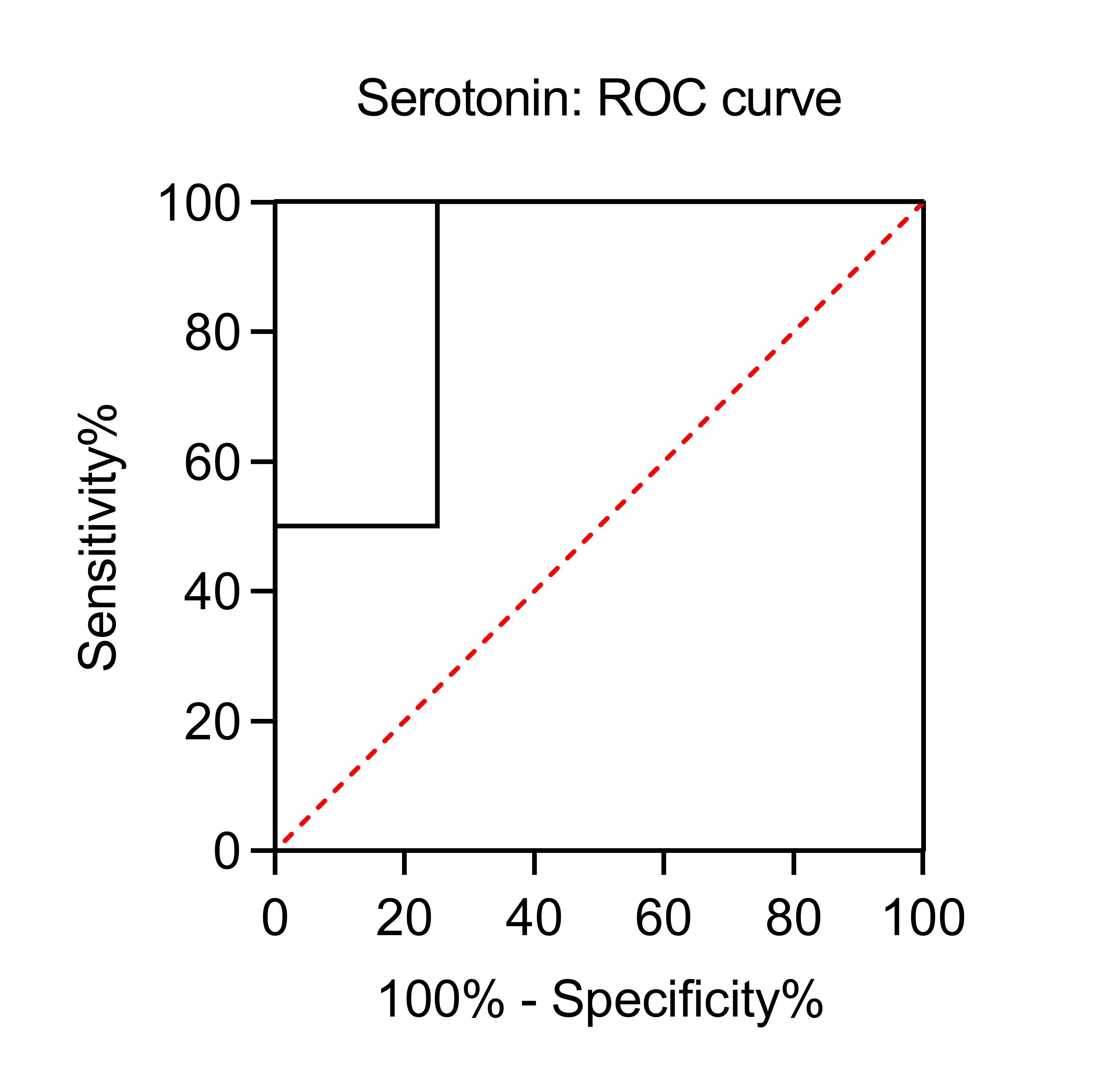

Supplement: Supplementary file 2 [file DataSheet1.zip › Supplementary File_ROC/neg_1997_Serotonin ROC curve (2).tif]

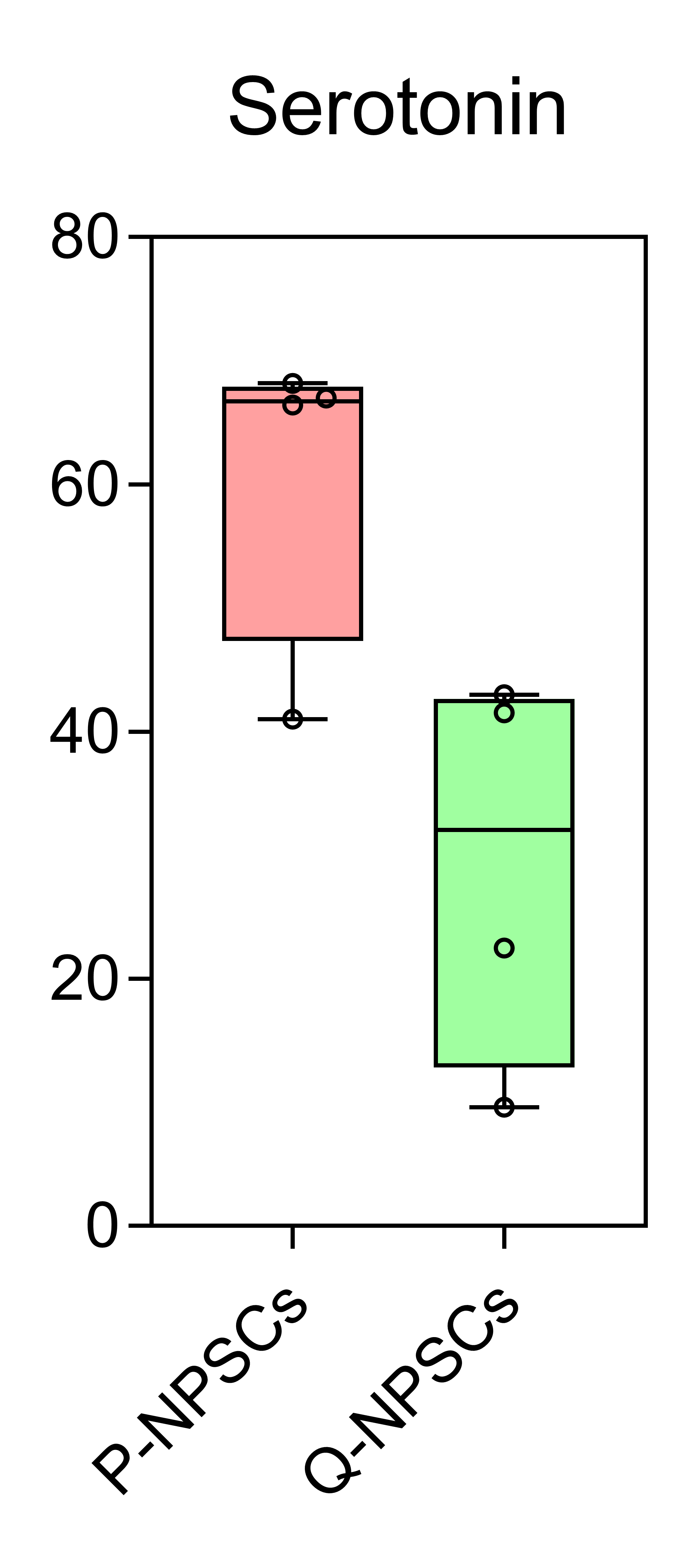

Supplement: Supplementary file 2 [file DataSheet1.zip › Supplementary File_ROC/neg_1997_Serotonin.tif]

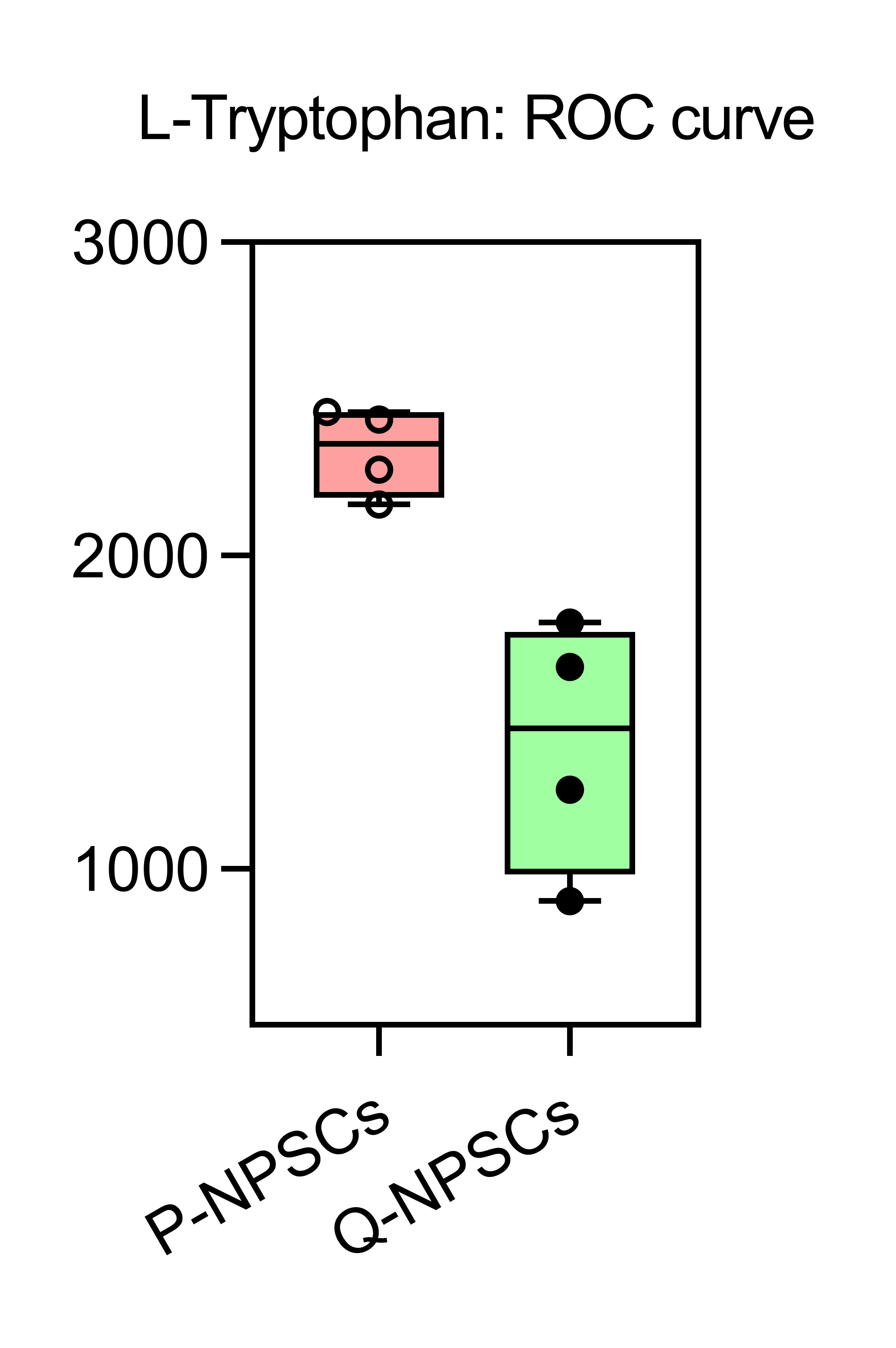

Supplement: Supplementary file 2 [file DataSheet1.zip › Supplementary File_ROC/neg_1998_L-Tryptophan ROC curve (1).tif]

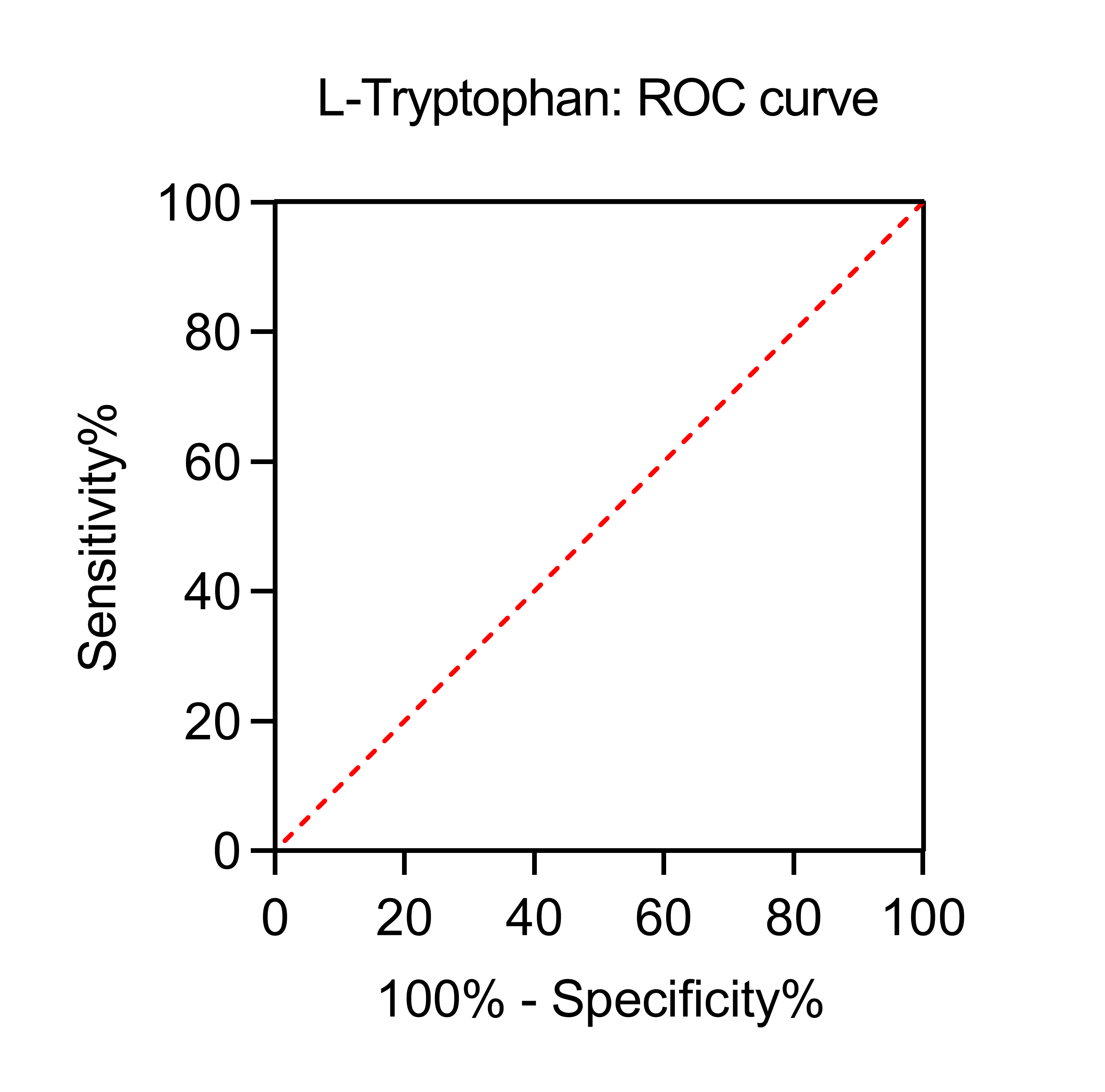

Supplement: Supplementary file 2 [file DataSheet1.zip › Supplementary File_ROC/neg_1998_L-Tryptophan ROC curve (2).tif]

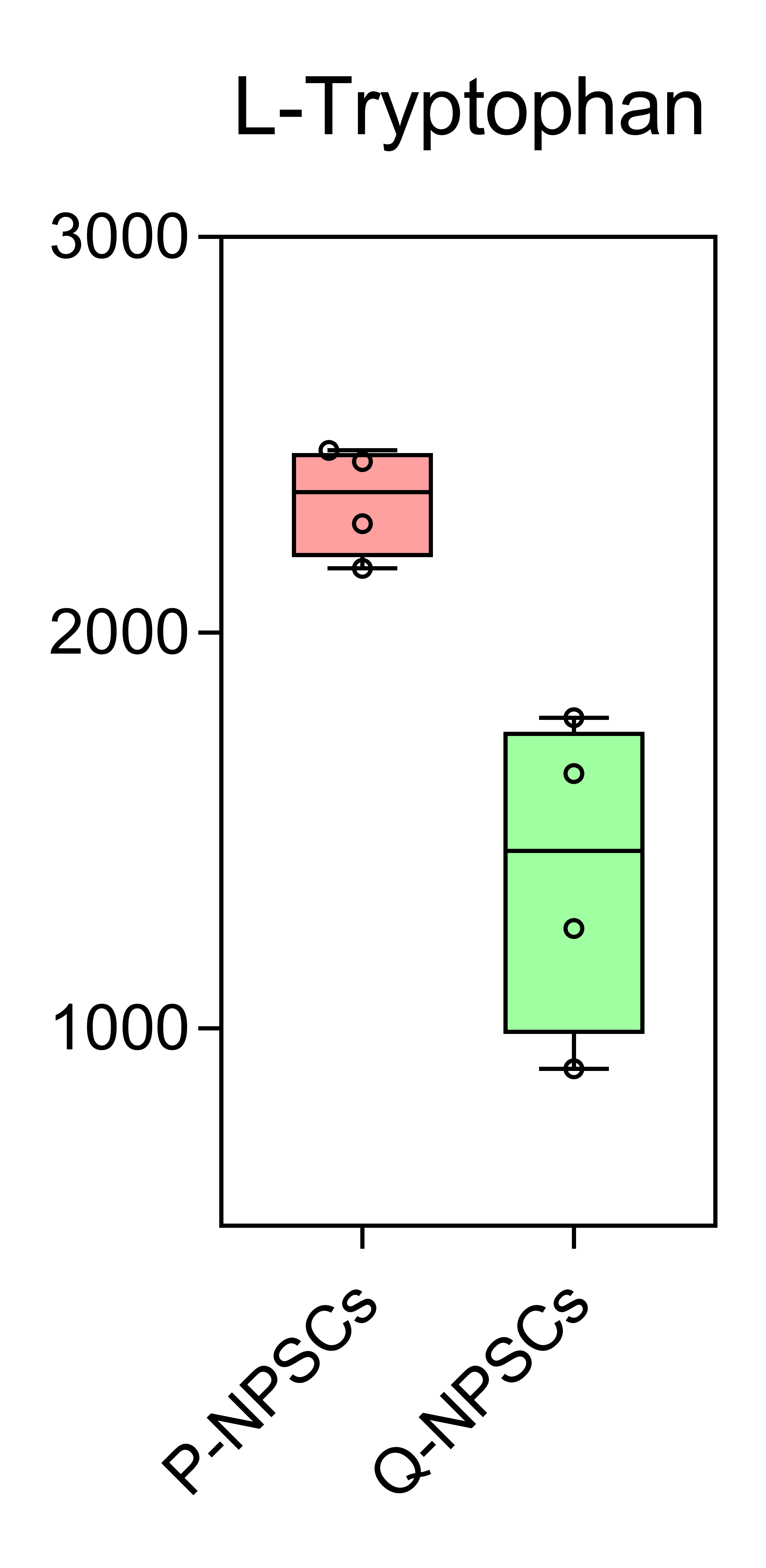

Supplement: Supplementary file 2 [file DataSheet1.zip › Supplementary File_ROC/neg_1998_L-Tryptophan.tif]

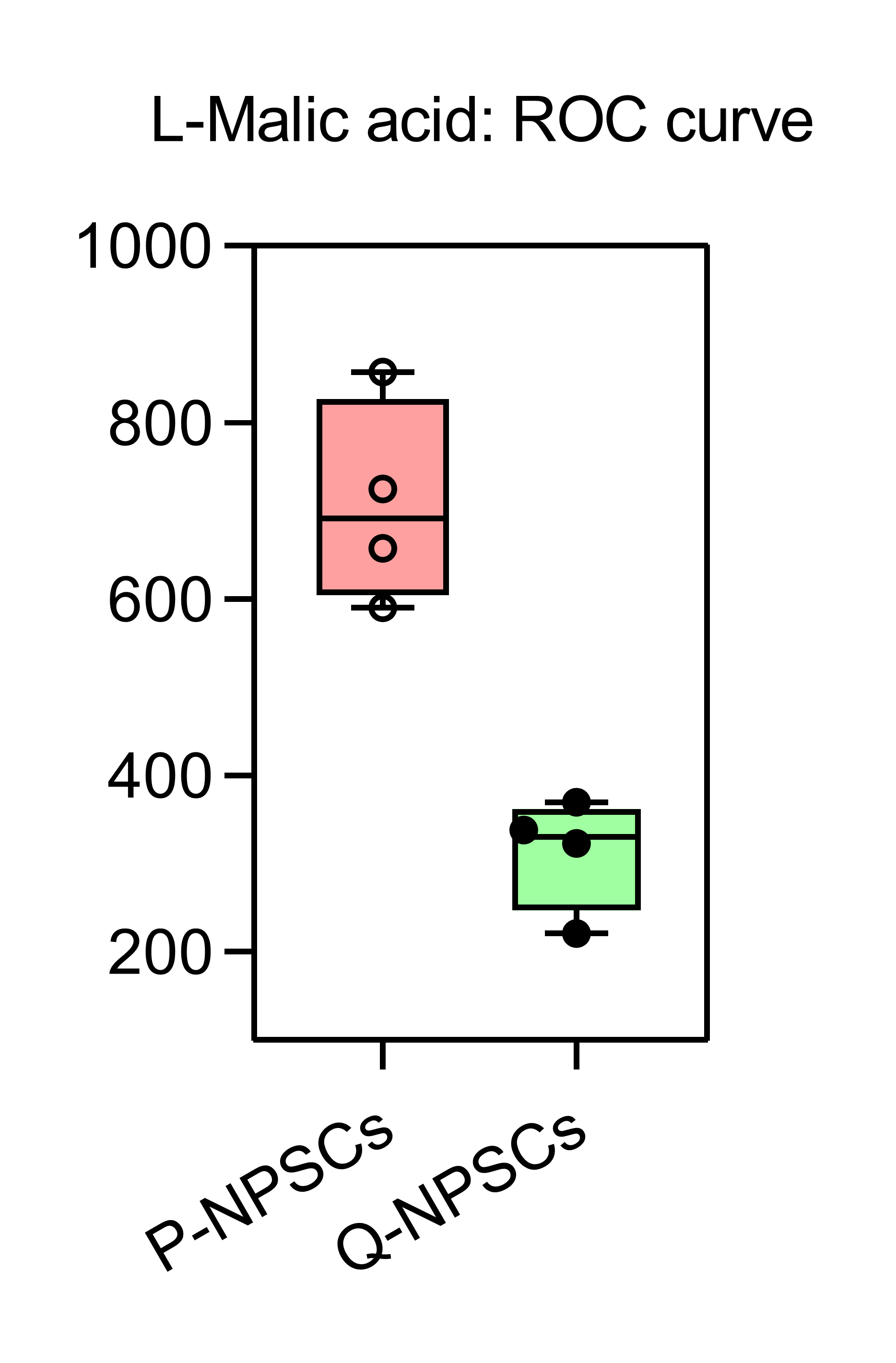

Supplement: Supplementary file 2 [file DataSheet1.zip › Supplementary File_ROC/neg_990_L-Malic acid ROC curve (1).tif]

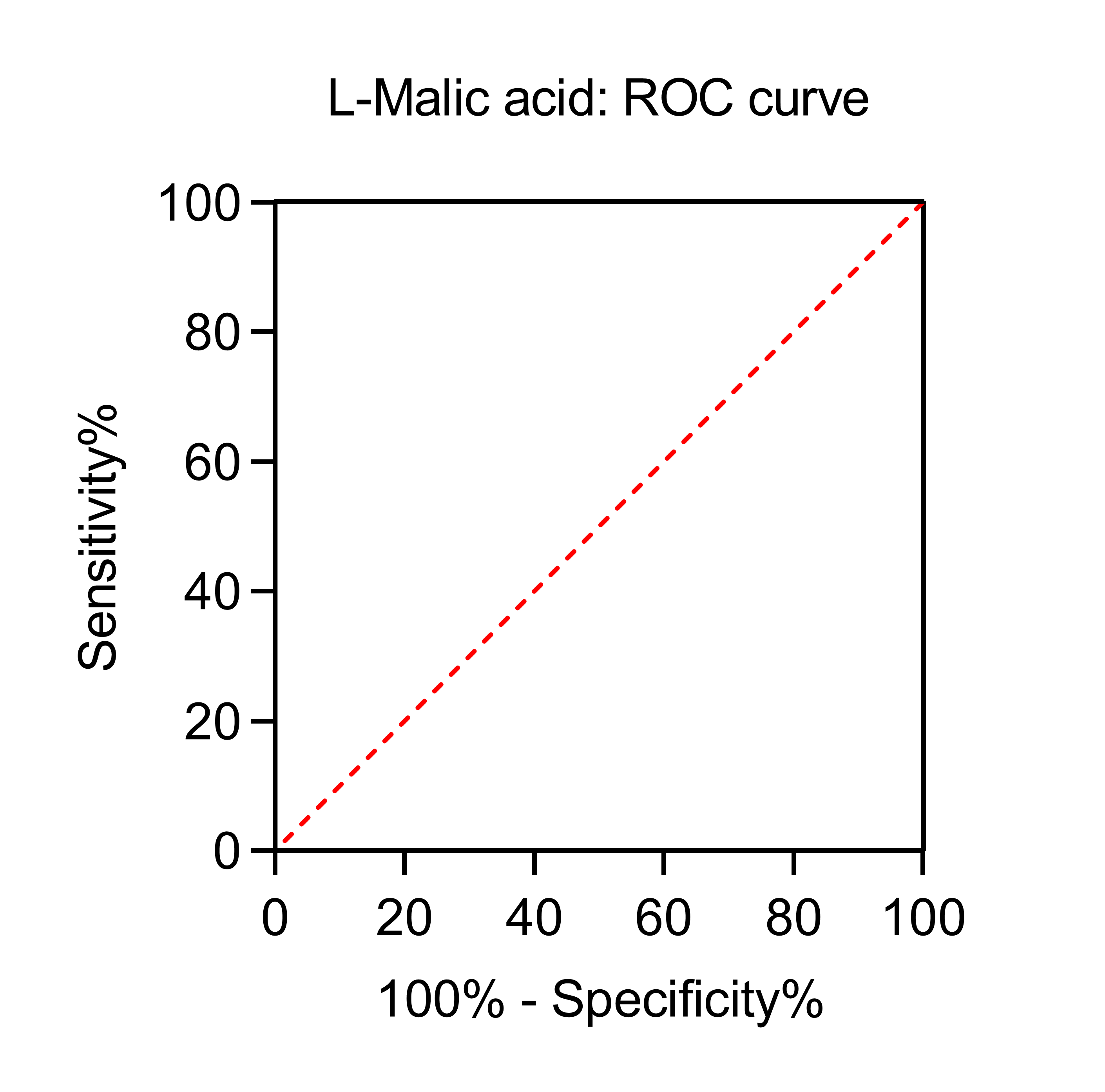

Supplement: Supplementary file 2 [file DataSheet1.zip › Supplementary File_ROC/neg_990_L-Malic acid ROC curve (2).tif]

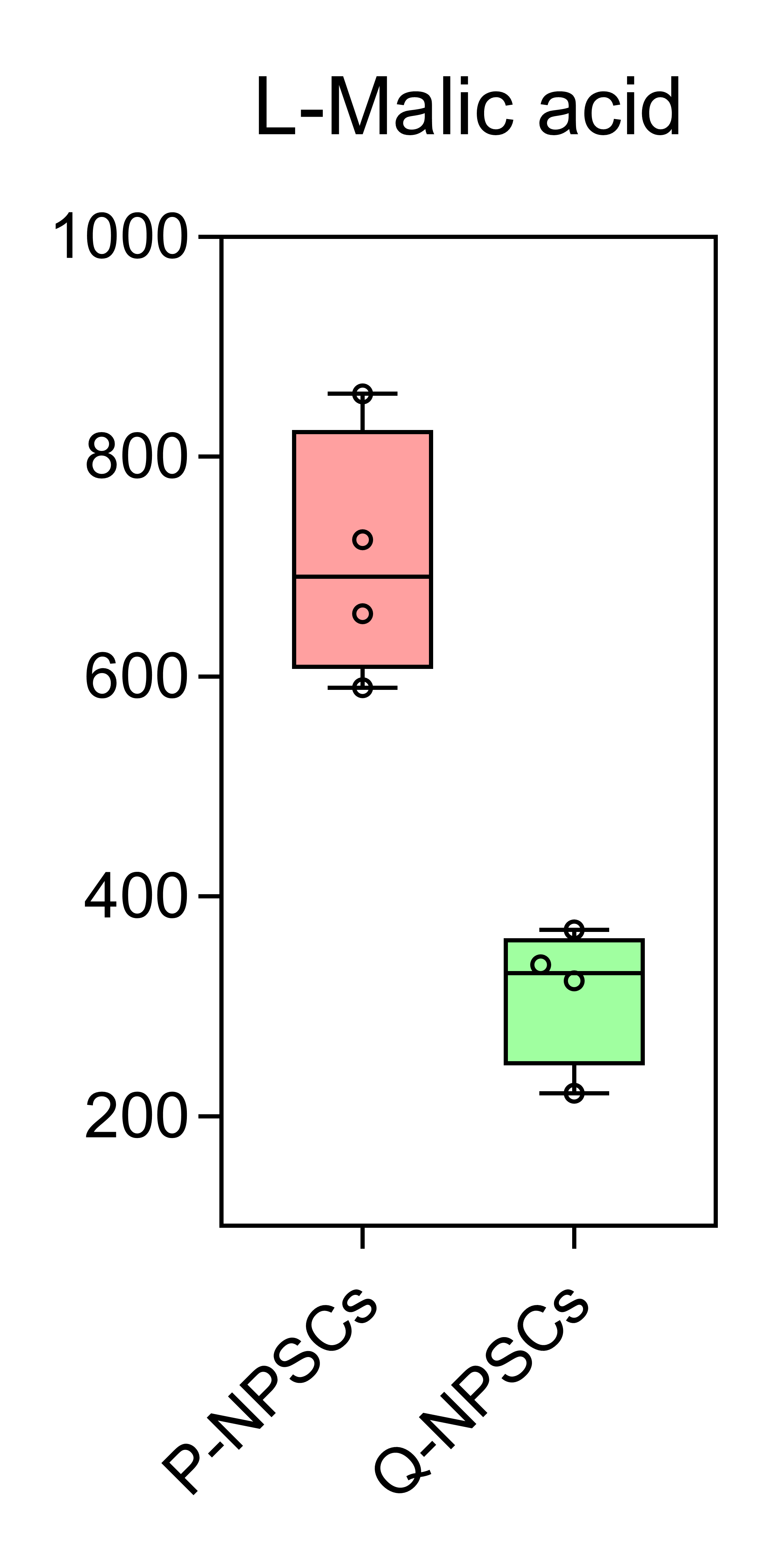

Supplement: Supplementary file 2 [file DataSheet1.zip › Supplementary File_ROC/neg_990_L-Malic acid.tif]
